# $\Gamma$-Limsup estimate for a nonlocal approximation of the Willmore functional

**DOI:** 10.1007/s00526-025-03039-w

**Published:** 2025-05-30

**Authors:** Hardy Chan, Mattia Freguglia, Marco Inversi

**Affiliations:** 1 Departement Mathematik Und Informatik, Universität Basel, 4051 Basel, Switzerland; 2 Scuola Normale Superiore, Piazza dei Cavalieri 7, 56126 Pisa, Italy

**Keywords:** 49J45, 26A33, 35R11

## Abstract

We propose a possible nonlocal approximation of the Willmore functional, in the sense of Gamma-convergence, based on the first variation of the fractional Allen–Cahn energies, and we prove the corresponding $\Gamma$-limsup estimate. Our analysis is based on the expansion of the fractional Laplacian in Fermi coordinates and fine estimates on the decay of higher order derivatives of the one-dimensional nonlocal optimal profile. This result is the nonlocal counterpart of that obtained by Bellettini and Paolini, where they proposed a phase-field approximation of the Willmore functional based on the first variation of the (local) Allen–Cahn energies.

## Introduction

In recent years, there has been a growing interest in geometric energies, such as the Area functional or the Willmore functional, the latter being defined by

$$\begin{aligned} \mathcal {W}(\Sigma , \Omega ) = \int _{\Sigma \cap \Omega } H_{\Sigma }^2(y) \, d \mathcal {H}^{d-1}(y), \end{aligned}$$

where $d \ge 2$ is an integer number, $\Omega \subset \mathbb {R}^d$ is an open set, $\Sigma \subset \mathbb {R}^d$ is a smooth hypersurface, $H_{\Sigma }(y)$ is the mean curvature of $\Sigma$ at the point *y* and $\mathcal {H}^{d-1}$ stands for the $(d-1)$-dimensional Hausdorff measure in $\mathbb {R}^d$. Among several questions related to this functional, a relevant problem consists in minimizing $\mathcal {W}$ within all the surfaces of a certain type, for instance with fixed genus [7, 34, 54]; or connected, closed, confined to a prescribed region and with fixed area [23, 24]; or with prescribed isoperimetric ratio [51]. Another problem that generates a lot of interest is the study of the geometric flow associated to $\mathcal {W}$, see among others [35–37, 55].

While both of these problems can be attacked directly, it could be useful to approximate $\mathcal {W}$ with *simpler* functionals, solve similar problems for the approximating functionals and then try to pass to the limit to obtain a solution to the original problems. Here, the notion of approximation considered is that of Gamma-convergence, see [21] for the definition and the fundamental properties, which is well suited for the convergence of the minima and the minimizers, and, under additional assumptions, also for the convergence of the flows, see [48, 52].

For the Area functional, this approach has been highly successful when considering the phase-transition regularization given by the Allen–Cahn energies, which are defined for any $\varepsilon > 0$ by

1.1 $$\begin{aligned} \mathcal {E}_{\varepsilon }(u, \Omega ) = \int _{\Omega } \bigg ( \frac{\varepsilon }{2} \left| \nabla u\right| ^2 + \frac{W(u)}{\varepsilon } \bigg ) \, dx, \end{aligned}$$

where $W :\mathbb {R}\rightarrow [0,+\infty )$ is a double-well potential, e.g. $W(s)=(1-s^2)^2$, which has wells at $s=\pm 1$ as we assume for convenience. From now on, in any statement concerning *W*, we will implicitly assume that it satisfies few structural assumptions, see Sect. 2.1 for the details.

In the foundational work [44] (see also [43]) Modica and Mortola proved that for any set $E \subset \mathbb {R}^d$ of finite perimeter $\textrm{Per}(E,\Omega )$ inside a Lipschitz domain $\Omega$ it holds

1.2 $$\begin{aligned} \Gamma (L^1(\Omega ))-\lim _{\varepsilon \rightarrow 0^+} \mathcal {E}_{\varepsilon }(\chi _E, \Omega ) = \sigma \,\textrm{Per}(E,\Omega ), \end{aligned}$$

where $\chi _E:= \mathbbm {1}_E - \mathbbm {1}_{E^c}$ denotes the difference between the characteristic functions of *E* and $E^c$, while $\sigma$ is a positive constant depending on the potential *W*. For further details about the notation, see Sect. 2.1.

The convergence of (constrained) critical points of $\mathcal {E}_\varepsilon$ to “generalized” critical points of the Area functional (also when the ambient space is a closed manifold) has been studied by many authors, see for instance [31, 38, 43, 57, 59, 60] and the more recent results in [8, 9, 16, 17, 27, 29, 40, 41]. Moreover, the convergence of the rescaled $L^2$-gradient flows of $\mathcal {E}_\varepsilon$ to Brakke’s motion by mean curvature has been established in [32].

Now, it is well-known that the mean curvature represents the first variation of the Perimeter. Therefore, it is reasonable to consider a suitable $L^2$-norm of the first variation of $\mathcal {E}_{\varepsilon }$ to build an approximation for $\mathcal {W}$. Indeed, starting from a conjecture of De Giorgi stated in [20] (see also [10] for more about this conjecture), Bellettini and Paolini proposed to consider in [12] the following approximation of the Willmore functional:

1.3 $$\begin{aligned} \mathcal {W}_{\varepsilon }(u,\Omega ) = \frac{1}{\varepsilon } \int _{\Omega } \bigg ( \varepsilon \Delta u - \frac{W'(u)}{\varepsilon } \bigg )^2 \, dx. \end{aligned}$$

In addition, for any open set $E \subset \mathbb {R}^d$ with $\partial E \in C^2$, they proved the $\Gamma$-$\limsup$ estimate:

1.4 $$\begin{aligned} \Gamma (L^1(\mathbb {R}^d))-\limsup _{\varepsilon \rightarrow 0^+} \big ( \mathcal {E}_{\varepsilon } + \mathcal {W}_{\varepsilon } \big )(\chi _E, \mathbb {R}^d) \le \sigma \textrm{Per}(E,\mathbb {R}^d) + \sigma \mathcal {W}(\partial E, \mathbb {R}^d). \end{aligned}$$

To this end, they exhibited a family of functions converging to $\chi _E$ in $L^1$ for which the limit of the values of $\mathcal {E}_{\varepsilon } + \mathcal {W}_{\varepsilon }$ evaluated on this family equals the right-hand side of (1.4). They considered a slight modification of the rescaled transition profiles

1.5 $$\begin{aligned} u_\varepsilon (x):=q_0\bigg (\frac{\textrm{dist}_{\partial E}(x)}{\varepsilon }\bigg ), \end{aligned}$$

where $q_0$ is the one-dimensional optimal profile (i.e. the unique, up to translations, monotone increasing solution of the Euler–Lagrange equation of (1.1) for $d=1, \Omega =\mathbb {R}$ and $\varepsilon =1$) and $\textrm{dist}_{\partial E}$ is the signed distance function from the boundary of *E*. The $\Gamma$-$\liminf$ estimate completing (1.4) turns out to be more delicate, and it was proved by Röger and Schätzle in [47] when $d \in \{ 2, 3\}$ and by Nagase and Tonegawa in [45] for $d=2$ with different techniques, while being still open in higher dimension.

The aim of the present paper is to prove a nonlocal counterpart of the estimate (1.4) exploiting the approximation of the (local) Perimeter given by the fractional Allen–Cahn energies. Given $s \in \left( {1}/{2},1\right)$ and $\varepsilon > 0$, the *s*-fractional Allen–Cahn energy is defined by

$$\begin{aligned}  &   \mathcal {F}_{s,\varepsilon }(u, \Omega ) = \varepsilon ^{2s-1} \frac{\gamma _{d,s}}{4} \iint _{\mathbb {R}^d \times \mathbb {R}^d \setminus (\Omega ^c \times \Omega ^c)} \frac{\left| u(x)-u(y)\right| ^2}{\left| x-y\right| ^{d+2s}} \, dx \, dy + \int _{\Omega } \frac{W(u(x))}{\varepsilon } \, dx, \end{aligned}$$

where $\gamma _{d,s}$ is given by (2.2). The starting point of our analysis is the work by Savin and Valdinoci [49] where, among other results, they established a nonlocal version of (1.2). Specifically, they proved that for any set $E \subset \mathbb {R}^d$ of finite perimeter $\textrm{Per}(E,\Omega )$ inside a Lipschitz domain $\Omega$ it holds

1.6 $$\begin{aligned} \Gamma (L^1_{\textrm{loc}}(\mathbb {R}^d))-\lim _{\varepsilon \rightarrow 0^+} \mathcal {F}_{s,\varepsilon }(\chi _E, \Omega ) = c_{\star } \textrm{Per}(E,\Omega ), \end{aligned}$$

where $c_{\star }$ is a positive constant depending on *s*, *d*, *W* and the functionals $\mathcal {F}_{s,\varepsilon }$ are thought to be defined on those functions in $L^{\infty }(\mathbb {R}^d)$ that take values between the zeros of *W* (see [18] for similar results on more general functionals of nonlocal type). This result holds also for $s={1}/{2}$, but with a different scaling in $\varepsilon$, that is with an extra $\left| \log (\varepsilon )\right| ^{-1}$ factor in front of $\mathcal {F}_{s,\varepsilon }$. We mention also [2] for related results in the case $d=1$ and $s= {1}/{2}$. Similarly to the local case, the estimate from above in the previous theorem is achieved by considering functions of the form of (1.5), at least when the set *E* meets the boundary of $\Omega$ transversely, and where $q_0$ is replaced by the nonlocal one-dimensional optimal profile *w*. In particular, $w :\mathbb {R}\rightarrow (-1,1)$ is defined as the unique, up to translations, monotone increasing solution of the fractional Allen–Cahn equation

1.7 $$\begin{aligned} (-\partial _{zz})^s w + W'(w) = 0, \end{aligned}$$

where $(-\partial _{zz})^s$ is the fractional Laplacian of order 2*s* in dimension one. We refer to Sect. 2.4 for a collection of some well-known properties of *w*. More in general, the first variation of $\mathcal {F}_{s,\varepsilon }$ is represented by $\varepsilon ^{2s-1}(-\Delta )^s u + \varepsilon ^{-1} W'(u)$, where $(-\Delta )^s$ denotes the *s*-fractional Laplacian (see Sect. 2.3 for precise definitions). At this point, it is natural to consider the following nonlocal version of the functional defined by (1.3), that is

1.8 $$\begin{aligned} \mathcal {G}_{s,\varepsilon }(u, \Omega ) = \frac{1}{\varepsilon } \int _{\Omega } \bigg ( \varepsilon ^{2s-1} (-\Delta )^s u + \frac{W'(u)}{\varepsilon } \bigg )^2 \, dx, \end{aligned}$$

if $u \in L^{\infty }(\mathbb {R}^d) \cap C^2(\Omega )$, and $\mathcal {G}_{s,\varepsilon }(u, \Omega ):= +\infty$ otherwise in $L^1_{\textrm{loc}}(\mathbb {R}^d)$. Here, the nonlocal behaviour of the functional is encoded in the fractional Laplacian. We are ready to state our main result.

### Theorem 1.1

Let $s \in \left( {3}/{4},1\right)$. Then, for any bounded open set $E \subset \mathbb {R}^d$ with $\partial E \in C^2$ it holds

1.9 $$\begin{aligned} \Gamma (L^1_{\textrm{loc}}(\mathbb {R}^d))-\limsup _{\varepsilon \rightarrow 0^+} \big (\mathcal {F}_{s,\varepsilon } + \mathcal {G}_{s,\varepsilon }\big )(\chi _E,\Omega ) \le c_{\star } \textrm{Per}(E, \Omega ) + \kappa _{\star } \mathcal {W}(\partial E, \Omega ), \end{aligned}$$

where $\Omega \subset \mathbb {R}^d$ is any bounded open set with $\partial \Omega \in C^1$, $c_{\star }$ is the constant in (1.6) and $\kappa _{\star }$ is a positive constant depending only on *s* and *W*. In the case $s={3}/{4}$ the same conclusion holds if in the definition of $\mathcal {G}_{s,\varepsilon }$ we add an extra $\left| \log (\varepsilon )\right| ^{-1}$ factor in front of the integral.

We recall the definition of the $\Gamma$-$\limsup$, more precisely

$$\begin{aligned}  &   \Gamma (L^1_{\textrm{loc}}(\mathbb {R}^d))-\limsup _{\varepsilon \rightarrow 0^+} \big (\mathcal {F}_{s,\varepsilon } + \mathcal {G}_{s,\varepsilon }\big )(\chi _E,\Omega ) \\  &   = \inf \left\{ \limsup _{\varepsilon \rightarrow 0^+} (\mathcal {F}_{s,\varepsilon }+\mathcal {G}_{s,\varepsilon })(u_\varepsilon ,\Omega ) :u_\varepsilon \rightarrow \chi _E \text { in }L^1_{\text {loc}}(\mathbb {R}^d) \right\} . \end{aligned}$$

The constant $\kappa _\star$ is defined by (6.1). We mention that without the regularity assumption on $\Omega$ an analogous of (1.9) still holds, that is with ${\overline{\Omega }}$ on the right-hand side (see Remark 6.2). Before discussing the strategy of the proof, some comments are in order.

- We point out that we consider the sum of $\mathcal {F}_{s,\varepsilon }$ and $\mathcal {G}_{s,\varepsilon }$ in order to rule out the behaviour of certain trivial sequences that are uniformly bounded with respect to the functionals $\{ \mathcal {G}_{s,\varepsilon } \}$ but unbounded for the functionals $\{ \mathcal {F}_{s,\varepsilon } \}$. In particular, the ultimate goal is to show that there is a relationship between the limit of $\mathcal {G}_{s,\varepsilon }(u_\varepsilon )$ and the limit of $\mathcal {F}_{s,\varepsilon }(u_\varepsilon )$ when both are uniformly bounded in $\varepsilon > 0$. Since $\mathcal {F}_{s,\varepsilon }$ and $\mathcal {G}_{s,\varepsilon }$ are non-negative functionals, it is reasonable to consider their sum. An example of a trivial sequence that we want to exclude from our analysis is the following. We know from Rolle’s Theorem that there exists $c_0 \in (-1,1)$ such that $W'(c_0)=0$. For any $\varepsilon > 0$, we consider $u_\varepsilon \equiv c_0$, then it holds that $\mathcal {G}_{s,\varepsilon }(u_\varepsilon ) = 0$. On the other hand, since $W(c_0) > 0$, we have
  $$\begin{aligned} \lim _{\varepsilon \rightarrow 0^+} \mathcal {F}_{s,\varepsilon }(u_\varepsilon ) = \lim _{\varepsilon \rightarrow 0^+} \frac{W(c_0)}{\varepsilon } \mathcal {L}^d(\Omega ) = +\infty . \end{aligned}$$
- The need of a different scaling for $s={3}/{4}$ mildly suggests that the previous result might no longer be true when $s \in \left[ {1}/{2},{3}/{4}\right)$. Maybe, for these values of the parameter *s* the limit of the functionals $\mathcal {F}_{s,\varepsilon }+\mathcal {G}_{s,\varepsilon }$ (with a different scaling in $\varepsilon$ for $\mathcal {G}_{s,\varepsilon }$) could be equal to the sum of the (local) Perimeter and a combination of the Willmore functional and a nonlocal quantity possibly depending on the nonlocal mean curvature (for the definition see, for example, [1] or [25, Section 6]). We refer to Sect. 6 for further comments.
- On the other hand, when $s \in \left( 0,{1}/{2}\right)$ the situation is fairly different. Already for the functionals $\mathcal {F}_{s,\varepsilon }$ it is not longer true that they approximate the classical (local) Perimeter. In [49], Savin and Valdinoci showed that the rescaled functionals $\varepsilon ^{1-2s} \mathcal {F}_{s,\varepsilon }$ approximate the 2*s*-fractional Perimeter and that any family of minimizers with equibounded energy converges to a minimizer of the 2*s*-fractional Perimeter in $\Omega$. In [42], the previous convergence was extended to more general families $\{ u_{\varepsilon } \}$ of critical points of $\varepsilon ^{1-2s} \mathcal {F}_{s,\varepsilon }$ having equibounded energy. In particular, the authors proved that $u_{\varepsilon }$ converges in a quite strong sense to the characteristic function of a set $E_{*}$ such that $\partial E_{*} \cap \Omega$ is a stationary 2*s*-fractional minimal surface in $\Omega$. Roughly speaking, $\partial E_{*}$ has vanishing 2*s*-fractional mean curvature on $\Omega$. Moreover, they obtained compactness results also for family of functions $\{ u_\varepsilon \}$ with equibounded energy and a uniform Sobolev bound on their first variations. These results are the nonlocal analogies, in the range $s \in \left( 0,{1}/{2}\right)$, of those in [31, 58] in the local case.
- In the case $s \in \left[ {3}/{4},1\right)$, it would be interesting to know if a full Gamma-convergence result holds, namely, if the functionals $\mathcal {F}_{s,\varepsilon } + \mathcal {G}_{s,\varepsilon }$ Gamma-converge to the right-hand side of (1.9), at least in small dimensions and within the class of $C^2$ sets, where the corresponding result in the local case is known to be true [45, 47]. More in general, understanding the compactness properties of families of functions having $\mathcal {F}_{s,\varepsilon } + \mathcal {G}_{s,\varepsilon }$ equibounded would be of interest. However, up to our knowledge the situation is not clear even for families $\{ u_\varepsilon \}$ of critical points of $\mathcal {F}_{s,\varepsilon }$ having equibounded energy. It is reasonable to expect a similar behaviour to the local case, where the energies of the critical points concentrate towards a “generalized” critical point of the Perimeter, we refer again to [31]. In order to prove Theorem 1.1 we need to exhibit, for any set $E \subset \mathbb {R}^d$ with $\partial E$ of class $C^2$, a family of functions $\{ u_{\varepsilon } \} \subset L^{\infty }(\mathbb {R}^d) \cap C^2(\Omega )$ converging to $\chi _E$ in $L^1_{\textrm{loc}}(\mathbb {R}^d)$ such that
  1.10 $$\begin{aligned} \lim _{\varepsilon \rightarrow 0^+} \mathcal {F}_{s,\varepsilon }(u_{\varepsilon },\Omega ) = c_{\star } \textrm{Per}(E,\Omega ) \quad \text {and} \quad \lim _{\varepsilon \rightarrow 0^+} \mathcal {G}_{s,\varepsilon }(u_{\varepsilon },\Omega ) = \kappa _{\star } \mathcal {W}(\partial E, \Omega ). \end{aligned}$$
  If *E* is a smooth set with ${\overline{E}} \subset \Omega$, then a natural candidate for $u_{\varepsilon }(x)$ would be $w_\varepsilon (\textrm{dist}_{\partial E}(x))$, where $w_\varepsilon (z) = w\left( {z}/{\varepsilon }\right)$ and *w* is the nonlocal one-dimensional optimal profile (see Sect. 2.4). Indeed, as mentioned earlier, this family of functions is a recovery sequence for the $\Gamma$-limit of $\mathcal {F}_{s,\varepsilon }$, meaning that the first identity in (1.10) holds true when considering this family of functions. On the other hand, while the signed distance function $\textrm{dist}_{\partial E}$ is globally 1-Lipschitz, it is smooth only near the boundary of *E*, and therefore $\mathcal {G}_{s,\varepsilon }$ has infinite value at $w_\varepsilon ( \textrm{dist}_{\partial E}(x))$. To overcome this issue, we introduce a suitable extension $\beta _{\partial E} \in C^2(\mathbb {R}^d)$ of $\textrm{dist}_{\partial E}$ outside a fixed small tubular neighbourhood of $\partial E$ (see Sect. 2.6). Accordingly, we consider the family of functions defined by $u_{\varepsilon }(x):=w_\varepsilon (\beta _{\partial E}(x))$. We point out here that the first identity in (1.10) remains true also when considering this family of functions, this follows by the same argument used in [49]. After doing so, our strategy to prove the second identity in (1.10) is similar to the one adopted in [12]. We split $\mathcal {G}_{s,\varepsilon }(u_\varepsilon , \Omega )$ into the sum of two contributions, the first one being the integral in a small tubular neighbourhood of $\partial E$ and the second one being the contribution coming from the complement of this tubular neighbourhood. However, the analysis of these two quantities is technically more involved than in the local case. We also remark that it is also quite different from the approach used in [49] to obtain the $\Gamma$-limsup estimate for the functionals $\{ F_{s,\varepsilon } \}$ alone. Indeed, in our case we need a precise description of the fractional Laplacian of the function $u_\varepsilon$ near the interface $\partial E$, as well as an accurate asymptotic of the higher-order derivatives of the nonlocal one-dimensional optimal profile *w*. Both of these aspects were not necessary for the corresponding estimate in [49]. As mentioned shortly before, in order to estimate the contribution of $\mathcal {G}_{s,\varepsilon }(u_\varepsilon , \Omega )$ away from the interface, we require fine estimates on the decay of the higher order derivatives of *w*. While such estimates could be known by the experts, we were not able to find them explicitly stated in the literature. For the local one-dimensional optimal profile $q_0$ they are well-known (see for instance [11]). In particular, borrowing ideas from [5, 33] we deduce the following result.

### Theorem 1.2

Let $s \in (0,1)$ and $k \ge 2$. In addition to our structural assumptions, suppose that $W \in C^{k+1}(\mathbb {R})$. Letting *w* be the nonlocal one-dimensional optimal profile, then $w\in C^{k} (\mathbb {R})$ and there exists a positive constant $C = C(s,W,k)$ such that

1.11 $$\begin{aligned} \left| \partial _x^k w(x)\right| \le \frac{C}{1+ \left| x\right| ^{k+2s}}, \qquad \forall x \in \mathbb {R}. \end{aligned}$$

The estimate in the theorem above is coherent with the fact that $w'$ is asymptotic to $\left| x\right| ^{-1-2s}$, as proved in [13, Theorem 2.7]. See also Remark 3.2 for further discussions.

To deal with the contribution coming from a small tubular neighbourhood $\mathcal {U}_{\delta }$ of $\partial E$ of size $\delta$, it is convenient to use Fermi coordinates (see Sect. 2.7 for the precise definition). More precisely, we identify any $x \in \mathcal {U}_{\delta }$ with a couple (*y*, *z*), where $y \in \partial E$ is the point of minimum distance between *x* and $\partial E$ and $z=\textrm{dist}_{\partial E}(x)$. Using these coordinates the (local) Laplacian can be written as

1.12 $$\begin{aligned} \Delta = \partial _{zz} - H_z \partial _z + \Delta _{\Sigma _z}, \end{aligned}$$

where $H_z$ denotes the mean curvature (with respect to its outer normal) of the hypersurface $\Sigma _z:= \partial \{ x \in \mathcal {U}_{\delta } :\textrm{dist}_{\partial E}(x) > z \}$, which is diffeomorphic and parallel to $\partial E$ and $\Delta _{\Sigma _z}$ is the Laplace–Beltrami operator on $\Sigma _z$. One advantage of these coordinates is that whenever *u* is a function that depends only on the distance from the boundary of *E*, then the computation of its Laplacian considerably simplifies. In contrast, a similar expression to (1.12) for the fractional Laplacian has been derived only recently. Indeed, the first-named author studied Fermi coordinates in the context of the fractional Laplacian in his PhD Thesis and he introduced suitable expansions for the fractional Laplacian in [14, 15], in collaboration with Liu and Wei. Roughly speaking, if $s \in \left( {1}/{2},1\right)$, $u_\varepsilon$ and $w_\varepsilon$ are defined as above, then we have the following expansion

1.13 $$\begin{aligned} (-\Delta )^s u_{\varepsilon }(x) = (-\partial _{zz})^s w_{\varepsilon }(z) + H_{\partial E}(y) L_s[w_{\varepsilon }'](z) + \mathcal {R}_{\varepsilon }(y,z), \end{aligned}$$

where $L_{s}$ is a suitable operator evaluated at $w'_{\varepsilon }$ and $\mathcal {R}_{\varepsilon }(y,z)$ is an error term. In addition, the first term on the right-hand side in the previous expansion scales as $\varepsilon ^{-2s}$, the second one scales as $\varepsilon ^{1-2s}$, while the remainder term is uniformly bounded in $\varepsilon$. We refer to Sect. 4 for the precise statement and a detailed proof.

In conclusion, we briefly comment about the assumption $s \in \left[ {3}/{4},1\right)$. If we neglect the error term in (1.13), then from (1.7) and (1.8) we obtain the following asymptotic

1.14 $$\begin{aligned} \mathcal {G}_{s, \varepsilon }(u_\varepsilon , \mathcal {U}_{\delta })  &   \simeq \varepsilon ^{4s-3} \Vert {L_{s}[w_{\varepsilon }']} \Vert ^2_{L^2((-\delta , \delta ))} \mathcal {W}(\partial E, \mathcal {U}_{\delta })\nonumber \\  &   =\Vert {L_{s}[w']} \Vert ^2_{L^2\left( \left( -{\delta }/{\varepsilon }, {\delta }/{\varepsilon }\right) \right) } \mathcal {W}(\partial E, \mathcal {U}_{\delta }). \end{aligned}$$

Moreover, we will see in Sect. 5 that $L_{s}[w'] \in L^2(\mathbb {R})$ when $s \in ({3}/{4},1)$, while for $s={3}/{4}$ the norm in the right-hand side of (1.14) diverges logarithmically in $\varepsilon$. We refer to Sect. 6 for a more detailed description of the heuristic argument as well as the proof of Theorem 1.1.

The paper is organised as follows. In Sect. 2 we discuss some geometric lemmas and we deduce some useful decay properties of the nonlocal one-dimensional optimal profile *w*. In Sect. 3 we discuss the proof of Theorem 1.2. Section 4 is entirely devoted to the expansion of the fractional Laplacian in Fermi coordinates. In this section, we follow the presentation of [14], adapting some of their results to our framework. In Sect. 5 we show that the constant $\kappa _{\star }$ appearing in (1.9) is finite. In Sect. 6 we combine our results to conclude the proof of Theorem 1.1.

## Tools

In this section we collect some tools which will be needed. We start by introducing some notation that we keep throughout the manuscript.

### Notation

- We denote by $\Gamma ((\mathbb {X},d)) - \lim _{\varepsilon } F_\varepsilon$, $\Gamma ((\mathbb {X},d)) - \limsup _{\varepsilon } F_\varepsilon$ and $\Gamma ((\mathbb {X},d)) - \liminf _{\varepsilon } F_\varepsilon$ the $\Gamma$-limit, the $\Gamma$-limsup, and the $\Gamma$-liminf, as $\varepsilon \rightarrow 0$, of a family of functions $F_\varepsilon : \mathbb {X} \rightarrow \mathbb {R}\cup \{ +\infty \}$ with respect to the metric *d* on $\mathbb {X}$. For the definitions see for instance [19].
- We denote by *W* a double-well potential satisfying the following structural assumptions:

(*W*1): $W: \mathbb {R}\rightarrow [0, +\infty )$, *W* is even and $\{W(x) = 0 \} = \{\pm 1\}$;
(*W*2): $$W \in C^{3}(\mathbb {R})$$;
(*W*3): $$W''(\pm 1) = \lambda > 0$$;

- we denote by $\mathcal {W}$ the Willmore functional, $\text {Per}$ the Perimeter functional, $\mathcal {F}_{s,\varepsilon }$ the scaled nonlocal Allen–Cahn energy and $\mathcal {G}_{s,\varepsilon }$ the squared $L^2$-norm of its first variation (Sect. 1);
- unless otherwise specified, we denote by *w* the one-dimensional optimal profile (Sect. 2.4) and $w_\varepsilon (z) = w({z}/{\varepsilon })$ and $u_\varepsilon (x) = w_\varepsilon (\beta _{\Sigma }(x))$ (Definition 2.10);
- we denote by $[\cdot ]_{C^{k,\theta }}, \Vert {\cdot } \Vert _{C^{k,\theta }}$ the Hölder seminorms and norms (Sect. 2.2);
- we denote by ${\mathscr {F}}_d$ the Fourier transform in $\mathbb {R}^d$ (Sect. 2.3);
- we denote by $(-\Delta )^s$ the fractional Laplacian operator of power $s \in (0,1)$ (see (2.1));
- $\gamma _{d,s}$ is the constant given by (2.2);
- we denote by $P^{(s)}_d$ the fractional heat kernel of power *s* in $\mathbb {R}^d$ (Sect. 2.5);
- we denote by $G_{s,\lambda }$ the fundamental solution of $(-\Delta )^s + \lambda \textrm{Id}$ (Proposition 3.1);
- we denote by $B_\delta ^m = \{ x \in \mathbb {R}^m :\left| x\right| < \delta \}$ and $\mathcal {H}^m$ the *m*-dimensional Hausdorff measure;
- we denote by $\Omega \subset \mathbb {R}^d$ a bounded open set;
- we denote by $E \subset \mathbb {R}^d$ an open set of class $C^2$ as in Definition 2.11 and we set $\Sigma = \partial E$;
- we denote by $\chi _E = \mathbbm {1}_E - \mathbbm {1}_{E^c}$, where $\mathbbm {1}_E$ stands for the indicator function of the set *E*;
- we denote by $\textrm{dist}_{\Sigma }$ the signed distance from $\Sigma$ and $\beta _{\Sigma }$ the smoothed signed distance (Definition 2.10);
- for any $\ell >0$ we denote by $\Sigma _\ell = \textrm{dist}_{\Sigma }^{-1}((-\ell ,\ell ))$;
- we denote by $\pi _{\Sigma }: \Sigma _\ell \rightarrow \Sigma$ the projection onto $\Sigma$ (Lemma 2.9);
- given an open set $E \subset \mathbb {R}^d$, for any $x \in \partial E = \Sigma$ we denote by $T_x \Sigma , N_\Sigma (x), H_\Sigma (x)$ the tangent space to $\Sigma$ at *x*, the inner unit normal to $\Sigma$ at *x* and the scalar mean curvature of $\Sigma$ at *x* computed with respect to $-N_\Sigma (x)$, respectively;
- given an open set $E \subset \mathbb {R}^d$ and $x_0 \in \partial E = \Sigma$, we denote by $Y = \textrm{Id}\times g$ a principal parameterization around $x_0$, *N* the inner unit normal vector given by the parameterization *g*, $k_1, \dots , k_{d-1}$ the principal coordinates at $x_0$ and $\Phi (y,z) = Y(y) + z N(y)$ the Fermi coordinated around $x_0$ (Definition 2.11);
- we denote by $\left| (Y(y) - z_0 e_d)_\tau \right|$ the tangential distance (Definition 2.14);
- we denote by $\eta _{\varepsilon , \ell }$ the function in (5.1);
- we denote by $c_\star ,\mu _w, \kappa _\star$ the constants in (1.6), (5.3) and (6.1).

### Function spaces

Given $\Omega \subset \mathbb {R}^d$ open, $\theta \in (0,1]$, $k \in \mathbb {N}$ and $u: \Omega \rightarrow \mathbb {R}$, we set

$$\begin{aligned}  &   [u]_{C^\theta (\Omega )} = \sup _{x,y \in \Omega , x \ne y} \frac{\left| u(x)-u(y)\right| }{\left| x-y\right| ^\theta }, \quad \Vert {u} \Vert _{C^\theta (\Omega )} = \Vert {u} \Vert _{L^\infty (\Omega )} + [u]_{C^\theta (\Omega )},\\  &   [u]_{C^k(\Omega )} = \sum _{\left| \alpha \right| = k}\Vert {\partial ^\alpha u} \Vert _{L^\infty (\Omega )}, \quad \Vert {u} \Vert _{C^k(\Omega )} = \Vert {u} \Vert _{L^\infty (\Omega )} + \sum _{j=1}^k [u]_{C^j(\Omega )},\\  &   [u]_{C^{k,\theta }(\Omega )} = \sum _{\left| \alpha \right| = k} [\partial ^\alpha _x u]_{C^\theta (\Omega )}, \quad \Vert {u} \Vert _{C^{k,\theta }(\Omega )} = \Vert {u} \Vert _{C^k(\Omega )} + [u]_{C^{k,\theta }(\Omega )}. \end{aligned}$$

### The fractional Laplacian

Here we recall the definition and some basic properties of the fractional Laplacian. We refer to [22, 26] for an extensive discussion on the topic. Given $s \in (0,1)$ and a function $u :\mathbb {R}^d \rightarrow \mathbb {R}$ globally bounded and of class $C^2$, the fractional Laplacian and $(-\Delta )^s u$ is defined by

2.1 $$\begin{aligned} (-\Delta )^s u(x) = \gamma _{d,s} P.V. \int _{\mathbb {R}^d} \frac{u(x) -u(y)}{\left| x-y\right| ^{d+2s}}\, dy = \gamma _{d,s} \lim _{\nu \rightarrow 0} \int _{B_\nu (x)^c} \frac{u(x) -u(y)}{\left| x-y\right| ^{d+2s}}\, dy , \end{aligned}$$

where the constant $\gamma _{d,s}$ is defined for convenience by

2.2 $$\begin{aligned} \gamma _{d,s}:= s 2^{2s} \pi ^{-\frac{d}{2}} \frac{\Gamma \left( \frac{d+2s}{2} \right) }{\Gamma (1-s)}. \end{aligned}$$

It is easy to check that for globally bounded functions of class $C^2$ it holds that

$$\begin{aligned} (-\Delta )^s u(x) = \frac{\gamma _{d,s}}{2} \int _{\mathbb {R}^d} \frac{2 u(x) - u(x+y) - u(x-y)}{\left| y\right| ^{d+2s}} \, dy, \end{aligned}$$

We define the Fourier transform by

$$\begin{aligned} {\mathscr {F}}_d u(\xi ) = \int _{\mathbb {R}^d} u(x) e^{2 \pi i x \cdot \xi } \, dx, \end{aligned}$$

so that the inversion formula reads as $({\mathscr {F}}_d^{-1} u ) (x) = ({\mathscr {F}}_d u) (-x)$. With this convention, it can be checked that (see e.g. [26])

$$\begin{aligned} {\mathscr {F}}_d [(-\Delta )^s u] (\xi ) = \left| 2\pi \xi \right| ^{2s} {\mathscr {F}}_d u(\xi ), \end{aligned}$$

The definition of the fractional Laplacian extends to tempered distributions by duality. We state and prove the following elementary estimate.

#### Lemma 2.1

Given $s \in (0,1)$ and $\Omega ^{\prime } \subset \! \subset \Omega ^{\prime \prime } \subset \! \subset \Omega \subset \mathbb {R}^d$ open sets, then for any $u \in L^\infty (\mathbb {R}^d) \cap C^{2}(\Omega )$ it holds

$$\begin{aligned} \Vert {(-\Delta )^s u} \Vert _{L^\infty (\Omega ^{\prime })} \le C(d,s,\Omega ^{\prime }, \Omega ^{\prime \prime }) \left( [u]_{C^{2}(\Omega ^{\prime \prime })} + \Vert {u} \Vert _{L^\infty (\mathbb {R}^d)} \right) . \end{aligned}$$

#### Proof

Let $\delta >0$ be such that $B_{\delta }(x) \subset \Omega ^{\prime \prime }$ for any $x \in \Omega ^{\prime }$. Then, up to a multiplicative constant $C(d,s,\Omega ^{\prime }, \Omega ^{\prime \prime })>0$, we have

$$\begin{aligned} \left| (-\Delta )^s u(x)\right|&\lesssim \int _{B_{\delta }^c(x)} \frac{\left| u(x) -u(y)\right| }{\left| x-y\right| ^{d+2s}} \, dy +\lim _{\nu \rightarrow 0} \left| \int _{B_{\delta }(x) \setminus B_\nu (x)} \frac{u(x) - u(y) - \nabla u(x) \cdot (y-x) }{\left| x-y\right| ^{d+2s}}\, dy\right| \\  &\lesssim \Vert {u} \Vert _{L^\infty (\mathbb {R}^d) } \int _{B_\delta ^c(x)} \frac{1}{\left| x-y\right| ^{d+2s}} \, dy + [u]_{C^2(\Omega ^{\prime \prime })} \int _{B_{\delta }(x)} \frac{1}{\left| x-y\right| ^{d+2s-2}} \, dy \\  &\lesssim \Vert {u} \Vert _{L^\infty (\mathbb {R}^d)} + [u]_{C^2(\Omega ^{\prime \prime })}. \end{aligned}$$

$$\square $$

### The one-dimensional optimal profile

Throughout the whole manuscript, we denote by $w: \mathbb {R}\rightarrow (-1,1)$ the unique increasing solution to the fractional Allen–Cahn equation

2.3 $$\begin{aligned} {\left\{ \begin{array}{ll} (-\partial _{zz} )^s w + W'(w) = 0, \\ w(0)=0, \\ \lim _{z \rightarrow \pm \infty } w(z) = \pm 1. \end{array}\right. } \end{aligned}$$

For the sake of clarity, we collect some well-known results about the optimal profile that will be needed in our analysis.

#### Proposition 2.2

Fix $s \in (0,1)$ and let *W* be a double-well potential satisfying (*W*1), (*W*2), (*W*3). There exists a unique strictly increasing solution $w: \mathbb {R}\rightarrow (-1,1)$ to the problem (2.3) Moreover, *w* is odd, $w \in C^1(\mathbb {R})$ and there exists $C(s,W)>0$ such that

2.4 $$\begin{aligned} \left| w'(z) \right| \le \frac{C}{1+ \left| z\right| ^{1+2s}}, \qquad \forall z \in \mathbb {R}. \end{aligned}$$

We recall that *w* is built by minimization of the $\mathcal {F}_{s,1}$ when $\Omega = \mathbb {R}$. More precisely, *w* is the unique (up to translation) minimizer of $\mathcal {F}_{s,1}(\cdot , \mathbb {R})$ with respect to perturbation with compact support in $\mathbb {R}$, see [46, Theorem 2] for instance. Since *W* is even, it is readily checked that *w* is odd. The regularity of *w* is established by iterating the apriori estimates in [53, Proposition 2.8$-$2.9]. As we already mentioned, the decay estimate (2.4) is optimal, see [13, Theorem 2.7]. Integrating (2.4), we find that

2.5 $$\begin{aligned} \left| w(z) - \mathrm{sgn\,}(z)\right| \le \frac{C}{1+ \left| z\right| ^{2s}} \qquad \forall z \in \mathbb {R}. \end{aligned}$$

Lastly, we recall that $w'$ solves the fractional Allen–Cahn equation

$$\begin{aligned} (-\partial _{zz})^s w' + W''(w) w' =0. \end{aligned}$$

### The fractional heat kernel

We consider the solution to the fractional heat equation

$$\begin{aligned} {\left\{ \begin{array}{ll} \partial _t P^{(s)}_d + (-\Delta )^s P^{(s)}_d = 0 &  (t, x) \in (0, +\infty ) \times \mathbb {R}^d, \\ P^{(s)}_d(0) = \delta _0 &  x \in \mathbb {R}^d, \end{array}\right. } \end{aligned}$$

where the initial value is taken in the sense of distributions. More precisely, $P^{(s)}_d$ is defined via the Fourier transform.

#### Definition 2.3

Fix $s \in (0,1)$. For $(t, x) \in (0, +\infty ) \times \mathbb {R}^d$, we define the fractional heat kernel

$$\begin{aligned} P^{(s)}_d(t,x):= \int _{\mathbb {R}^d} \exp \left( - t \left| 2\pi \xi \right| ^{2s} \right) e^{2 \pi i x \cdot \xi } \, d \xi = {\mathscr {F}}_d^{-1} \left( \exp { \left( - t \left| 2\pi (\cdot )\right| ^{2s}\right) }\right) (x), \end{aligned}$$

where ${\mathscr {F}}_d$ is the Fourier transform.

We mention [26, Chapter 16] and the references therein for a presentation of the topic. By a change of variables, it is easy to check that

2.6 $$\begin{aligned} P^{(s)}_d (t,x) = t^{-\frac{d}{2s}} P^{(s)}_d \left( 1, x t^{-\frac{1}{2s}}\right) . \end{aligned}$$

In analogy with the classical heat kernel, $P^{(s)}_d(t,x) >0$ for any (*t*, *x*) (see [26, Proposition 16.3] ). Since $\exp (- t \left| \xi \right| ^{2\,s})$ is a rapidly decaying function, then $P^{(s)}_d(t,\cdot )$ is smooth. However, due to lack of differentiability of $\exp (- t \left| \xi \right| ^{2s})$ at 0 for $s \in (0,1)$, then $P^{(s)}_d$ is not a Schwarz function. In particular, $P^{(s)}_d(1, \cdot )$ enjoys a polynomial decay, whereas the classical heat kernel decays exponentially.

#### Proposition 2.4

([26, Proposition 16.5]) Let $s \in (0,1)$ and $P^{(s)}_d(1, \cdot )$ be as in Definition 2.3. For any $x \in \mathbb {R}^d$ it holds that

$$\begin{aligned} \frac{C_1(d,s)}{1 + \left| x\right| ^{d+2s}} \le P^{(s)}_d(1, x) \le \frac{C_2(d,s)}{1 +\left| x\right| ^{d+2s}}. \end{aligned}$$

By the properties of the Fourier transform, for any index $j = 1, \dots , d$ we have

$$\begin{aligned} \partial _j P^{(s)}_{d}(1,x) = 2 \pi i {\mathscr {F}}_d^{-1}\left( \xi _j \exp \left( - \left| 2 \pi \xi \right| ^{2s} \right) \right) (x). \end{aligned}$$

Using the Bochner relation (see e.g. [56, Section 3.2]), we have

2.7 $$\begin{aligned} {\mathscr {F}}_d (\xi _j \exp (-\left| \cdot \right| ^{2s}))(x) = i x_j {\mathscr {F}}_{d+2} (\exp (-\left| \cdot \right| ^{2s})) ({\tilde{x}}), \end{aligned}$$

where we set ${\tilde{x}} = (x, 0,0) \in \mathbb {R}^d \times \mathbb {R}\times \mathbb {R}$. Hence, combining (2.7) and Proposition 2.4 we obtain the following result.

#### Proposition 2.5

Let $s \in (0,1)$ and $P^{(s)}_1(1, \cdot )$ be as in Definition 2.3. For any $k \in \mathbb {N}$ it holds

2.8 $$\begin{aligned} \left| \partial _x^k P^{(s)}_1(1,x)\right| \le \frac{C(k,s)}{1+ \left| x\right| ^{k+1+2s}} \qquad \forall x \in \mathbb {R}. \end{aligned}$$

#### Proof

We prove by induction that

2.9 $$\begin{aligned} \partial ^k_x P^{(s)}_1 (1, x) = \sum _{i= \lceil \frac{k}{2} \rceil }^k c_{i,k} x^{2i-k} P^{(s)}_{1+2i} (1, {\tilde{x}}), \qquad \forall x \in \mathbb {R} \end{aligned}$$

where $$c_{i,k} $$ are real-valued coefficients, $$\lceil \cdot \rceil $$ is the upper integer part and we denote by

$$\begin{aligned} P^{(s)}_{d}(1, {\tilde{x}}) = P^{(s)}_{d}(1, (x, 0, \cdots , 0)) \qquad \forall x \in \mathbb {R}. \end{aligned}$$

Then, (2.8) follows immediately by (2.9) and Proposition 2.4. To check the validity of (2.9), for $k=1$ by (2.7) we have

$$\begin{aligned} \partial _x P^{(s)}_1(1, x) = {\tilde{c}}_{1,1} x P^{(s)}_3(1, {\tilde{x}}). \end{aligned}$$

Assume that *k* is even, being the case of *k* odd similar. Then, deriving (2.9) we find explicit constants ${\tilde{c}}_{i,k}$ such that

$$\begin{aligned} \partial ^{k+1}_x P^{(s)}_1(1, x)&= \sum _{i= \lceil \frac{k}{2}\rceil +1 }^k c_{i,k} (2i-k) x^{2i-k-1} P^{(s)}_{1+2i}(1, {\tilde{x}}) + \sum _{i=\lceil \frac{k}{2}\rceil }^k {\tilde{c}}_{i,k} c_{i,k} x^{2(i+1)-k-1} P^{(s)}_{1+2(i+1)} (1, {\tilde{x}}) \\  &= \sum _{i= \lceil \frac{k+1}{2}\rceil }^k c^1_{i,k+1} x^{2i-(k+1)} P^{(s)}_{1+2i}(1, {\tilde{x}}) + \sum _{i=\lceil \frac{k}{2}\rceil +1 }^{k+1} c_{i,k+1}^2 x^{2i-(k+1)} P^{(s)}_{1+2i} (1, {\tilde{x}}). \end{aligned}$$

$$\square $$

### Distance function

Throughout this note, we adopt the following notation. Given an open set *E*, we denote by $\Sigma = \partial E$ and we let $\textrm{dist}_{\Sigma }$ be the signed distance from $\Sigma$, i.e.

$$\begin{aligned} \textrm{dist}_{\Sigma } (x) = {\left\{ \begin{array}{ll} \inf \{ \left| x-y\right| :y \in \Sigma \} &  \text { if } x \in E, \\ - \inf \{ \left| x-y\right| :y \in \Sigma \} &  \text { if } x \in E^c. \end{array}\right. } \end{aligned}$$

It is well known that the distance function is 1-Lipschitz continuous and $\left| \nabla \textrm{dist}_{\Sigma }(y)\right| =1$ at any point of differentiability. We refer to [3] and [28, Section 14.6] for an overview of the properties of the distance function.

#### Definition 2.6

Let $k \ge 2$. We say that $E \subset \mathbb {R}^d$ is an open set of class $C^k$ ($\partial E \in C^k$ in short) if for any $x_0 \in \Sigma = \partial E$ there exist $\delta > 0$ and a map $g :B_{\delta }^{d-1}\rightarrow \mathbb {R}$ of class $C^k$ such that, up to an affine isometry of the ambient space, it holds $x_0 =0$ and

2.10 $$\begin{aligned}&T_{x_0} \Sigma = \textrm{Span}(e_1,\dots ,e_{d-1}), \end{aligned}$$

2.11 $$\begin{aligned}&\Sigma \cap \big (B_\delta ^{d-1} \times (-\delta , \delta ) \big ) = \big \{(y, g(y)) :y \in B_\delta ^{d-1} \big \}, \end{aligned}$$

2.12 $$\begin{aligned}&E \cap \big (B_\delta ^{d-1} \times (-\delta , \delta ) \big ) = \big \{(y, t) :y \in B_\delta ^{d-1}, t > g(y) \big \}. \end{aligned}$$

Moreover, we say that the map $Y:= \textrm{Id} \times g :B^{d-1}_\delta \rightarrow \mathbb {R}^d$ is a principal parameterization of $\Sigma$ around $x_0$ if *g* satisfies

2.13 $$\begin{aligned} \nabla ^2 g(0) = \textrm{diag}(k_1, \dots , k_{d-1}), \end{aligned}$$

where $k_1, \dots , k_{d-1}$ denote the principal curvatures of $\Sigma$ at $x_0$ computed with respect to the outer unit normal.

#### Remark 2.7

Given $E \subset \mathbb {R}^d$ an open set of class $C^k$, then for any $x_0 \in \Sigma$ there exists a principal parameterization around $x_0$ according to Definition 2.6. Indeed, if *g* satisfies (2.10)–(2.12), then we find a linear isometry of $O: \mathbb {R}^{d-1} \rightarrow \mathbb {R}^{d-1}$ such that $g \circ O$ satisfies also (2.13).

#### Remark 2.8

In the framework of Definition 2.6, then $\Sigma$ is locally the graph of $C^2$ function $g :B^{d-1}_{\delta } \rightarrow \mathbb {R}$ and we have the following well-known formulas:

2.14 $$\begin{aligned}  &   T_{(y,g(y))} \Sigma = \textrm{Span}\big ( (e_1, \partial _1 g(y)),\dots ,(e_{d-1}, \partial _{d-1} g(y)) \big ), \nonumber \\  &   N(y) = \frac{(-\nabla g(y), 1)}{\sqrt{1+\left| \nabla g(y)\right| ^2}},\nonumber \\  &   H_{\Sigma }(y,g(y)) = \textrm{div} \left( \frac{\nabla g}{\sqrt{ 1 + \left| \nabla g\right| ^2}} \right) = \frac{\Delta g(y)}{\sqrt{ 1 + \left| \nabla g(y)\right| ^2}} - \frac{\nabla ^2 g(y)[\nabla g(y), \nabla g(y)]}{(1+\left| \nabla g(y)\right| ^2)^{3/2}},\nonumber \\ \end{aligned}$$

where *N*(*y*) denotes the inner unit normal to $\Sigma$ at the point (*y*, *g*(*y*)) and $H_{\Sigma }(y,g(y))$ is the scalar mean curvature of $\Sigma$ at the point (*y*, *g*(*y*)) computed with respect to $-N(y)$. In addition, if the function *g* satisfies

$$\begin{aligned} g(0)=0, \qquad \nabla g(0) = 0, \qquad \nabla ^2 g(0) = \textrm{diag}(k_1, \dots , k_{d-1}), \end{aligned}$$

then the previous formulas at $0 \in \Sigma$ simplify to

$$\begin{aligned} T_{0} \Sigma = \mathbb {R}^{d-1} \times \{ 0 \}, \qquad N(0) = (0,1), \qquad H_{\Sigma }(0) = \Delta g(0) = \sum _{i=1}^{d-1} k_i. \end{aligned}$$

We recall some well-known properties of the signed distance function from an hypersurface (see for instance [3, 28, Lemma 14.16], [43, Lemma 3]).

#### Lemma 2.9

Let $E \subset \mathbb {R}^d$ be a bounded open set of class $C^k$ for some $k \ge 2$. Then, there exists $\delta >0$ depending only on $\Sigma$ with the following properties.

(i) For any $x \in \Sigma _{\delta }$ there exists a unique point $\pi _{\Sigma }(x) \in \Sigma$ of minimal distance between *x* and $\Sigma$. Moreover, the map $\pi _{\Sigma } :\Sigma _\delta \rightarrow \Sigma$ is of class $C^{k-1}$.
(ii) For any $x \in \Sigma _\delta$ it holds that
  $$\begin{aligned} \nabla \textrm{dist}_{\Sigma }(x) = N(\pi _{\Sigma }(x)) , \end{aligned}$$
  where *N* is the inner unit normal to $\Sigma$. In particular, $\textrm{dist}_{\Sigma } \in C^{k}(\Sigma _\delta )$.

We consider a smooth version of the signed distance, which will play a key role in our analysis.

#### Definition 2.10

Let *E* be a bounded open set of class $C^2$ and let $0< \delta < {1}/{5}$ be such that $5\delta$ satisfies the properties of Lemma 2.9. We denote by $\beta _{\Sigma }: \mathbb {R}^d \rightarrow [-1,1]$ any function of class $C^2(\mathbb {R}^d)$ such that $\left| \beta _{\Sigma } (x)\right| \in [4\delta , 1]$ for any $x \in \Sigma _{5\delta } {\setminus } \Sigma _{4\delta }$ and

$$\begin{aligned} \beta _{\Sigma }(x) = {\left\{ \begin{array}{ll} \textrm{dist}_{\Sigma }(x) &  x \in \Sigma _{4\delta }, \\ \mathrm{sgn\,}(\textrm{dist}_{\Sigma }(x)) &  x \in \mathbb {R}^d \setminus \Sigma _{5\delta }. \end{array}\right. } \end{aligned}$$

### Uniform Fermi coordinates

We are interested in sets possessing parameterization with uniform bounds. Following [28, Section 14.6], we introduce a useful notation.

#### Definition 2.11

Let $E \subset \mathbb {R}^d$ be an open set. We say that $\Sigma = \partial E$ is of $(\delta ,C,k)$-type if *E* is of class $C^k$ and there exist positive constants $\delta$, *C* such that for any $x_0 \in \Sigma$ there exists a principal parameterization $Y=\textrm{Id} \times g :B_{\delta }^{d-1} \rightarrow \mathbb {R}^d$ around $x_0$ as in Definition 2.6 such that

2.15 $$\begin{aligned} \Vert {g} \Vert _{C^k\big (B_{\delta }^{d-1}\big )} \le C. \end{aligned}$$

Moreover, letting *N* be the inner unit normal defined by (2.14), we define the Fermi coordinates $\Phi : B_{\delta }^{d-1} \times (-\delta , \delta ) \rightarrow \mathbb {R}^{d}$ around $x_0$ by setting

$$\begin{aligned} \Phi (y,z):= Y(y) + z N(y). \end{aligned}$$

#### Remark 2.12

It is clear that if *E* is an open set of class $C^k$ and with compact boundary, then there exist positive constants $\delta _0$, $C_0$ such that $\Sigma$ is of $(\delta , C, k)$-type according to Definition 2.11 for any $\delta \le \delta _0$ and for any $C \ge C_0$. Since the proof follows by a straightforward compactness argument, we leave the details to the reader.

#### Remark 2.13

Let $E \subset \mathbb {R}^d$ be an open set of $(\delta , C, k)$-type for some $C, \delta >0$ and $k \ge 3$. With the notation of Definition 2.11, for any $x_0 \in \Sigma$ the map *g* can be expanded near $y=0$ as follows

2.16 $$\begin{aligned} g(y)&= \frac{1}{2} \sum _{i=1}^{d-1} k_i y_i^2 + O(\left| y\right| ^3), \end{aligned}$$

2.17 $$\begin{aligned} \partial _i g(y)&= k_i y_i +O(\left| y\right| ^2), \end{aligned}$$

2.18 $$\begin{aligned} \partial _{ij} g(y)&= \delta _{ij} k_i + O(\left| y\right| ). \end{aligned}$$

The reminders satisfy uniform estimates with respect to $x_0 \in \Sigma$ thanks to (2.15).

In view of proving Theorem 4.1, we need to compute integrals involving suitable powers of the Euclidean distance in small domains contained in the tubular neighbourhood of the boundary of a smooth set. Since it is natural to use Fermi coordinates as local charts of the tubular neighbourhood, it is convenient to write the Euclidean distance in terms of the Fermi coordinates.

#### Definition 2.14

Let $E \subset \mathbb {R}^d$ be an open set of $(\delta , C, k)$-type for some $C, \delta >0$ and $k \ge 3$, $x_0 \in \Sigma _\delta$, $x_0' = \pi _\Sigma (x_0)$ and $z_0 = \textrm{dist}_\Sigma (x_0)$. Let $\Phi$ be Fermi coordinates around $x_0'$. For any $y \in B_\delta ^{d-1}$, we define the (squared) tangential distance from $x_0$ by setting

2.19 $$\begin{aligned} \left| (Y(y) - z_0 e_d)_\tau \right| ^2:= \left| Y(y) - z_0 e_d\right| ^2 - \left| (Y(y) - z_0 e_d)\cdot N(y)\right| ^2. \end{aligned}$$

We refer to Remark 2.16 for a geometric interpretation of the tangential distance. We recall some Taylor expansions that will be useful later on, when we integrate on domains contained in the tubular neighbourhood using Fermi coordinates.

#### Lemma 2.15

Let $E \subset \mathbb {R}^d$ be an open set such that $\Sigma = \partial E$ is of $(\delta , C, 3)$-type according to Definition 2.11, for some $\delta , C >0$. Let $x_0 \in \Sigma _\delta$, let $x_0' = \pi _\Sigma (x_0)$ and let $z_0 = \textrm{dist}_\Sigma (x_0)$. Let $\Phi$ be Fermi coordinates around $x_0'$ and $\left| (Y(y)-z_0 e_d)_\tau \right|$ be the tangential distance given by Definition 2.14. Then, the following estimates hold true:

2.20 $$\begin{aligned}  &   Y(y) \cdot N(y) = - \frac{1}{2} \sum _{i=1}^{d-1} k_i y_i^2 + O(\left| y\right| ^3), \end{aligned}$$

2.21 $$\begin{aligned}  &   1 - N_d(y) = O(\left| y\right| ^2), \end{aligned}$$

2.22 $$\begin{aligned}  &   \nabla \Phi (0,z) = \textrm{diag}(1- z k_1, \dots , 1- z k_{d-1}, 1), \end{aligned}$$

2.23 $$\begin{aligned}  &   \det ( \nabla \Phi (y,z)) = \prod _{i=1}^{d-1}(1- z k_i) + O(\left| y\right| \left| z\right| ) + O(\left| y\right| ^2), \end{aligned}$$

2.24 $$\begin{aligned}  &   \left| (Y(y) - z_0 e_d)_\tau \right| ^2 = \sum _{1=1}^{d-1} y_i^2(1-k_i z_0)^2 + O(\left| z_0\right| \left| y\right| ^3) + O(\left| y\right| ^4). \end{aligned}$$

The error terms in (2.20)–(2.24) satisfy uniform bounds with respect to $x_0 \in \Sigma _\delta \cap E$.

#### Remark 2.16

With the notation of Definition 2.14, we have that $x_0 = \Phi (0, z_0)$. By completing the squares with respect to *z*, it is easy to check that

$$\begin{aligned} \left| \Phi (y,z) -\Phi (0,z_0)\right|&= \left| z-z_0 + Y(y)\cdot N(y) + z_0(1-N_d(y))\right| ^2 + \left| (Y(y) - z_0 e_d)_{\tau }\right| ^2. \end{aligned}$$

Moreover, by Lemma 2.15, we infer that

$$\begin{aligned} \left| z- z_0 + Y(y)\cdot N(y) + z_0(1- N_d(y))\right| = \left| z- z_0\right| + O(\left| y\right| ^2), \end{aligned}$$

which is the size of the “normal” component of $\Phi (y, z)- \Phi (0, z_0)$. By (2.24), $\left| (Y(y) - z_0 e_d)_{\tau }\right|$ is proportional to the size of the “tangential” variable stretched by the curvature of $\Sigma$ at $\pi _\Sigma (x_0)$.

#### Proof of Lemma 2.15

The proof is a direct computation and we include it for the reader’s convenience. By (2.16), (2.17), we have

$$\begin{aligned} Y(y) \cdot N(y)&= \frac{g(y) - \nabla g(y) \cdot y}{\sqrt{1 + \left| \nabla g(y)\right| ^2}} = \left( - \frac{1}{2} \sum _{i=1}^{d-1} y_i^2 k_i + O(\left| y\right| ^3) \right) \left( 1 + O(\left| y\right| ^2) \right) , \end{aligned}$$

thus proving (2.20). Similarly, we check (2.21), that is

$$\begin{aligned} 1- N_d(y) = 1- \frac{1}{\sqrt{1 + \left| \nabla g(y)\right| ^2 }} = O(\left| \nabla g(y)\right| ^2) = O(\left| y\right| ^2 ). \end{aligned}$$

In order to check (2.22) and (2.23), computing explicitly $\nabla \Phi (y,z)$, we have

$$\begin{aligned} \nabla \Phi (y,z) = \begin{pmatrix} 1+ z \partial _1 N_1 &  z \partial _2 N_1 &  \cdots &  z \partial _{d-1} N_1 &  N_1 \\ z \partial _1 N_2 &  1+ z \partial _2 N_2 &  \cdots &  z \partial _{d-1} N_2 &  N_2 \\ \vdots &  \vdots &  \ddots &  \vdots &  \vdots \\ z \partial _1 N_{d-1} &  z \partial _2 N_{d-1} &  \cdots &  1+ z \partial _{d-1} N_{d-1} &  N_{d-1} \\ \partial _1 g + z \partial _1 N_d &  \partial _2 g + z \partial _{2} N_{d} &  \cdots &  \partial _{d-1}g + z \partial _{d-1} N_d &  N_d \end{pmatrix}, \end{aligned}$$

Then, using (2.17), (2.18), for $i, j = 1, \dots , d-1$, we compute

$$\begin{aligned} \partial _i N_j = \frac{\partial _{ik} g \partial _i g \partial _k g - \partial _{ij} g (1 + \left| \nabla g\right| ^2)}{(1+ \left| \nabla g\right| ^2)^{3/2}} = -\delta _{ij} k_i + O(\left| y\right| ),\\ \partial _i g + z \partial _i N_d = \left( 1- \frac{z}{(1+ \left| \nabla g\right| ^2)^{3/2}} \right) \partial _i g = O(\left| y\right| ).\\ N_i = \frac{-\partial _i g}{\sqrt{1 + \left| \nabla g\right| ^2}} = O(\left| y\right| ), \ \ \ N_d = 1+ O(\left| y\right| ^2) \end{aligned}$$

where *g*, *N* are always computed at *y*. Hence, we obtain that

$$\begin{aligned} \nabla \Phi (y,z) = \begin{pmatrix} 1 - z k_1 + O(\left| y\right| \left| z\right| ) &  \cdots &  O(\left| y\right| \left| z\right| ) &  O(\left| y\right| ) \\ O(\left| y\right| \left| z\right| ) &  \cdots &  O(\left| y\right| \left| z\right| ) &  O(\left| y\right| ) \\ \vdots &  \ddots &  \vdots &  \vdots \\ O(\left| y\right| \left| z\right| ) &  \cdots &  1 - z k_{d-1} + O(\left| y\right| \left| z\right| ) &  O(\left| y\right| ) \\ O(\left| y\right| ) &  \cdots &  O(\left| y\right| ) &  1+ O(\left| y\right| ^2) \end{pmatrix}. \end{aligned}$$

Evaluating at (0, *z*), then (2.22) is proved. To compute the determinant, expanding with respect to the last row, it results that

$$\begin{aligned} \det (\nabla \Phi (y,z)) = \prod _{i=1}^{d-1} (1- z k_i + O(\left| y\right| \left| z\right| )) + O(\left| y\right| ^2) + O(\left| y\right| \left| z\right| ), \end{aligned}$$

thus proving (2.23). To conclude, we check the validity of (2.24). By (2.19), (2.16), (2.17) it holds

$$\begin{aligned}&\left| (Y(y) - z_0 e_d )_\tau \right| ^2 = \sum _{i=1}^{d-1} y_i^2 + (g(y) - z_0)^2 - \frac{1}{1 + \left| \nabla g(y)\right| ^2} \left( - \sum _{i=1}^{d-1} y_i \partial _i g(y) + (g(y) - z_0) \right) ^2\\&\quad = \left| y\right| ^2 + (g(y)-z_0)^2 - (1- \left| \nabla g(y)\right| ^2 + O(\left| y\right| ^4)) \left( - 2g(y) + O(\left| y\right| ^3) + g(y)-z_0 \right) ^2\\&\quad = \left| y\right| ^2 + (g(y)-z_0)^2 - (g(y)+z_0 + O(\left| y\right| ^3))^2 + \left| \nabla g(y)\right| ^2 (g(y)+z_0 + O(\left| y\right| ^3))^2 + O(\left| y\right| ^4)\\&\quad = \left| y\right| ^2 + (g(y)-z_0)^2 - (g(y)+z_0)^2 + \sum _{i=1}^{d-1} y_i^2 k_i ^2 (g(y)+z_0 + O(\left| y\right| ^3))^2 + O(\left| z_0\right| \left| y\right| ^3)\\&\quad = \left| y\right| ^2 - 4g(y) z_0 + \sum _{i=1}^{d-1}y_i^2 k_i ^2 z_0^2 + O(\left| z_0\right| \left| y\right| ^3) + O(\left| y\right| ^4)\\&\quad = \left| y\right| ^2 - 2 \sum _{i=1}^{d-1} y_i^2 k_i z_0 + \sum _{i=1}^{d-1}y_i^2 k_i ^2 z_0^2 + O(\left| z_0\right| \left| y\right| ^3) + O(\left| y\right| ^4), \end{aligned}$$

thus proving (2.24).

The following lemma is needed to justify the change of variables in the proof of Theorem 4.1.

#### Lemma 2.17

Under the assumptions of Lemma 2.15, there exists a constant $\Lambda _0\ge 1$ depending on $\Sigma$ such that for any $\Lambda \ge \Lambda _0$ for any $x_0 \in \Sigma _{{\delta }/{10\Lambda }}$ it holds that

2.25 $$\begin{aligned} {\mathscr {B}}_{{\delta }/{\Lambda }}^{d-1}(z_0):= \left\{ y \in \mathbb {R}^{d-1} :\left| (\textrm{Id}- z_0 \nabla ^2 g(0)) y\right| < {\delta }/{\Lambda } \right\} \subset B_{{\delta }/{2} }^{d-1}, \end{aligned}$$

where as before $z_0 = \textrm{dist}_{\Sigma }(x_0)$. In addition, for any $y \in {\mathscr {B}}_{{\delta }/{\Lambda } }^{d-1}(z_0)$ it holds that

2.26 $$\begin{aligned} {\mathscr {I}}_{{\delta }/{\Lambda } }(y,z_0):= \left\{ z \in \mathbb {R}:\left| z-z_0 + Y(y)\cdot N(y) + z_0(1-N_d(y))\right| < {\delta }/{\Lambda } \right\} \subset \left( -{\delta }/{2}, {\delta }/{2} \right) . \end{aligned}$$

Then, denoting by

2.27 $$\begin{aligned} \mathcal {B}_{{\delta }/{\Lambda }} (z_0):= \left\{ (y,z) \in {\mathscr {B}}_{{\delta }/{\Lambda }}^{d-1}(z_0) \times \mathbb {R}:z \in {\mathscr {I}}_{{\delta }/{\Lambda }}(y, z_0) \right\} , \end{aligned}$$

it holds that $\mathcal {B}_{{\delta }/{\Lambda }}(z_0) \subset B_{{\delta }/{2}}^{d-1}\times \left( -{\delta }/{2}, {\delta }/{2} \right)$. Finally, we have that $B_{{\delta }/{10 \Lambda }} (x_0) \subset \Phi (\mathcal {B}_{{\delta }/{\Lambda }}(z_0))$.

#### Proof

Let $\Lambda \ge 1$ be a constant, which will be fixed shortly. We remark that $\left| k_i \right| \le C$ for any $i=1, \dots , d-1$ (see (2.15)). We know that $\left| z_0\right| \le {\delta }/{10 \Lambda }$, therefore for any $y \in {\mathscr {B}}_{{\delta }/{\Lambda }}^{d-1}(z_0)$, by the triangular inequality, it is clear that

2.28 $$\begin{aligned} \left| y\right| \le \frac{\delta }{\Lambda } \left( 1- \frac{C \delta }{10 \Lambda } \right) ^{-1}. \end{aligned}$$

Hence, (2.25) is satisfied provided that

2.29 $$\begin{aligned} \frac{\delta }{\Lambda } \left( 1- \frac{C \delta }{10 \Lambda } \right) ^{-1} \le \frac{\delta }{2}. \end{aligned}$$

From Lemma 2.15, there exists a constant ${\bar{C}} > 0$ depending on $\Sigma$ such that for any $\Lambda \ge 1$ we have

2.30 $$\begin{aligned} \left| Y(y) \cdot N(y) + z_0( 1- N_d(y) )\right| \le {\bar{C}} \left| y\right| ^2 \qquad \forall y \in {\mathscr {B}}^{d-1}_{{\delta }/{\Lambda }}(z_0). \end{aligned}$$

Thus, given $y \in {\mathscr {B}}^{d-1}_{{\delta }/{\Lambda }}(z_0)$ and $z \in {\mathscr {I}}_{{\delta }/{\Lambda }}(y, z_0)$, by the triangular inequality, (2.28) and (2.30) it holds that

$$\begin{aligned} \left| z\right| \le \frac{\delta }{\Lambda } + \frac{\delta }{10 \Lambda } + {\bar{C}} \frac{\delta ^2}{\Lambda ^2} \left( 1- \frac{C \delta }{10 \Lambda } \right) ^{-2}. \end{aligned}$$

Hence, we infer that (2.26) is satisfied provided that

2.31 $$\begin{aligned} \frac{\delta }{\Lambda } + \frac{\delta }{10 \Lambda } + {\bar{C}} \frac{\delta ^2}{\Lambda ^2} \left( 1- \frac{C \delta }{10 \Lambda } \right) ^{-2} \le \frac{\delta }{2}. \end{aligned}$$

To summarize, if $\Lambda$ satisfies (2.29) and (2.31), then $\mathcal {B}_{{\delta }/{\Lambda }}(z_0) \subset B_{{\delta }/{2}}^{d-1}\times \left( -{\delta }/{2}, {\delta }/{2}\right)$. More precisely, using again the triangular inequality, (2.28) and (2.30), it can be checked in the same way that $B^{d-1}_{{\delta }/{2\Lambda }} \times \left( z_0 - {\delta }/{2\Lambda }, z_0 + {\delta }/{2\Lambda } \right) \subset \mathcal {B}_{{\delta }/{\Lambda }}(z_0)$ provided that the following condition is satisfied:

2.32 $$\begin{aligned} \max \left\{ \frac{\delta }{2\Lambda }\left( 1+ \frac{C \delta }{10 \Lambda } \right) , \frac{\delta }{2\Lambda } + {\bar{C}} \frac{\delta ^2}{4\Lambda ^2} \right\} \le \frac{\delta }{\Lambda }. \end{aligned}$$

Lastly, to prove that $B_{{\delta }/{10 \Lambda }} (x_0) \subset \Phi (\mathcal {B}_{{\delta }/{\Lambda }}(z_0))$ for $\Lambda$ sufficiently large, for any $t \in [0,1]$ we define the map ${\widehat{\Phi }}_t :B^{d-1}_{{\delta }/{2\Lambda }} \times \left( z_0 - {\delta }/{2\Lambda }, z_0 + {\delta }/{2\Lambda } \right) \rightarrow \mathbb {R}^d$ as follows

$$\begin{aligned} {\widehat{\Phi }}_t(y,z):= t (\Phi (y,z) - x_0) + (1-t) (y,z-z_0). \end{aligned}$$

Hence, using (2.22) we shall write

$$\begin{aligned} {\widehat{\Phi }}_t(y,z) = (y, z-z_0) - t z_0 (k_1 y_1, \dots , k_{d-1} y_{d-1}, 0) + O(\left| y\right| ^2 + \left| z-z_0\right| ^2). \end{aligned}$$

The previous formula implies that if $\Lambda$ is sufficiently large, then

2.33 $$\begin{aligned} |{\widehat{\Phi }}_t(y,z)| > {\delta }/{10 \Lambda }, \qquad \forall t \in [0,1], \forall (y,z) \in \partial \left( B_{{\delta }/{2 \Lambda }}^{d-1} \times (z_0 - {\delta }/{ 2 \Lambda }, z_0 + {\delta }/{2 \Lambda }) \right) .\nonumber \\ \end{aligned}$$

Therefore, for every $p \in B_{{\delta }/{ 10 \Lambda }}(0)$, the standard properties of the degree imply that

$$\begin{aligned} \textrm{deg}\Big ({\widehat{\Phi }}_1,p,B_{{\delta }/{2 \Lambda }}^{d-1} \times (z_0 - {\delta }/{ 2 \Lambda }, z_0 + {\delta }/{2 \Lambda })\Big )\\ = \textrm{deg}\Big ({\widehat{\Phi }}_0,p,B_{{\delta }/{2 \Lambda }}^{d-1} \times (z_0 - {\delta }/{ 2 \Lambda }, z_0 + {\delta }/{2 \Lambda })\Big ) = 1,\nonumber \\ \end{aligned}$$

and $B_{{\delta }/{10 \Lambda }} (x_0) \subset \Phi (\mathcal {B}_{{\delta }/{\Lambda }}(z_0))$. In particular, (2.29), (2.31)–(2.33) are satisfied if $\Lambda$ is large enough. $\square$

### Approximation of sets

The proof of Theorem 1.1 is more direct when the set *E* is smooth and intersects the boundary of $\Omega$ transversely (in a measure theoretic sense). To handle the general case, we approximate *E* with smooth bounded open sets such that both the Perimeter and the Willmore energy of the approximating sets in $\Omega$ converge to those of *E*. When $\Omega = \mathbb {R}^d$ there are several ways to construct such an approximation, see e.g. [6] and the references therein. In the following lemma we show that the same conclusion holds when $\Omega$ is a bounded open set of class $C^1$. Moreover, the approximating sets that we consider intersect the boundary of $\Omega$ transversely.

#### Lemma 2.18

Let $E \subset \mathbb {R}^d$ be a bounded open set with $\partial E \in C^2$. For any bounded open set $\Omega \subset \mathbb {R}^d$ with $\partial \Omega \in C^1$, there exists a sequence $\{ E_j \}_{j \in \mathbb {N}}$ of smooth bounded open sets of $\mathbb {R}^d$ such that

(A1): $$\displaystyle \lim _{j \rightarrow \infty } \left| E_j \Delta E\right| = 0$$,
(A2): $$\displaystyle \lim _{j \rightarrow \infty } \textrm{Per}(E_j,\Omega ) = \textrm{Per}(E,\Omega )$$,
(A3): $$\displaystyle \lim _{j \rightarrow \infty } \mathcal {W}(\partial E_j, \Omega ) = \mathcal {W}(\partial E, \Omega )$$,
(A4): $$\displaystyle \lim _{j \rightarrow \infty } \mathcal {H}^{d-1}(\partial E_j \cap \partial \Omega ) = 0$$,
(A5): $$\displaystyle \sup _{j \in \mathbb {N}} \Vert {H_{\partial E_j}} \Vert _{C^0(\partial E_j)} \le \Vert {H_{\partial E}} \Vert _{C^0(\partial E)} +1.$$

#### Proof

To begin, we show that there exists a sequence of sets of class $C^2$ with the above properties. Since $\partial \Omega$ is of class $C^1$, then $N_{\partial \Omega } \in C(\partial \Omega ; \mathbb {S}^{d-1})$, where $N_{\partial \Omega }$ denotes the inner unit normal to $\Omega$. Hence, we find a smooth vector field $X \in C^{\infty }_c(\mathbb {R}^d; \mathbb {R}^d)$ such that

2.34 $$\begin{aligned} \langle X(x), N_{\partial \Omega }(x) \rangle \le - \frac{1}{2}, \qquad \forall x \in \partial \Omega . \end{aligned}$$

For any $t >0$, we define $f_t(x):=x+tX(x)$. In particular, since *X* has compact support, there exists $t_0 > 0$ such that $f_t$ is a diffeomorphism of class $C^\infty$ for any $t \in (0,t_0]$. We consider the set

$$\begin{aligned} E_t:=f_t(E), \end{aligned}$$

and we claim that there exists a sequence of positive real numbers $\{ t_j \}_{j \in \mathbb {N}}$ converging to zero for which the corresponding sets $E_j:=E_{t_j}$ fulfill (*A*1), (*A*2), (*A*3), (*A*4) and (*A*5).

Letting $g_t:= f_t^{-1}$, we prove that for *t* small enough $g_t$ pushes $\Omega$ inside, that is there exists $t_1 \in (0,t_0]$ such that $g_t(\Omega ) \subset \Omega$ for any $t \in (0,t_1]$. Suppose by contradiction that there exist a sequence of positive real numbers $\{ t_n \}_{n \in \mathbb {N}}$ converging to zero and a sequence of points $\{ y_n \}_{n \in \mathbb {N}} \subset \Omega$ such that $x_n:=g_{t_n}(y_n) \in \mathbb {R}^{d} \setminus \Omega$. Since $\Omega$ is bounded, we can assume that $y_n$ converges to a limit point $z_{\infty } \in {\overline{\Omega }}$. On the other hand, $g_t$ converges uniformly to the identity map as $t \rightarrow 0$, therefore $x_n$ converges to the same limit point $z_{\infty }$. Since $x_n \in \mathbb {R}^{d} \setminus \Omega$ for any $n \in \mathbb {N}$, we have that $z_{\infty } \in \partial \Omega$. Using a local chart near $z_{\infty }$, we reduce to the case $z_\infty = 0$ and $\Omega = \{ x \in \mathbb {R}^d :x_d < 0 \}$. Hence, it is clear that $(y_n - x_n)/t_n$ is pointing inside $\Omega$. On the other hand, by (2.34) and the definition of $f_t$, we have

$$\begin{aligned} \lim _{n \rightarrow \infty } \Big \langle \frac{y_n-x_n}{t_n}, N_{\partial \Omega }(z_{\infty }) \Big \rangle  &   = \lim _{n \rightarrow \infty } \Big \langle \frac{f_{t_n}(x_n)-x_n}{t_n}, N_{\partial \Omega }(z_{\infty }) \Big \rangle \\  &   = \langle X(z_{\infty }), N_{\partial \Omega }(z_{\infty }) \rangle \le - \frac{1}{2}, \end{aligned}$$

which is a contradiction because $N_{\partial \Omega }(z_{\infty })$ is pointing inside $\Omega$.

Proof of (*A*1). For any $t \in (0,t_0]$, we have that $\partial E_t = f_t(\partial E) \in C^2$ since $f_t$ is a smooth diffeomorphism and $\partial E \in C^2$. Moreover, for any $x \in \mathbb {R}^d \setminus \partial E$, it holds that $\mathbbm {1}_{E_t}(x) \rightarrow \mathbbm {1}_{E}(x)$ as $t\rightarrow 0$. Indeed, for any $x \in \mathbb {R}^d, t \in (0,t_0]$ we have that

$$\begin{aligned} \mathbbm {1}_{f_t(E)}(x) = \mathbbm {1}_{f_t(E)} (f_t(g_t(x)) = \mathbbm {1}_{E}(g_t(x)). \end{aligned}$$

Since $g_t$ converges to the identity map uniformly as $t \rightarrow 0$ and *E* is open, for any $x \in E$ then $g_t(x) \in E$ we have that $g_t(x) \in E$ for *t* small enough. The same argument works for $x \in \mathbb {R}^d \setminus {\overline{E}}$, thus proving (*A*1).

Proof of (*A*2). Since the Perimeter is lower semicontinuous with respect to the $L^1$-convergence of sets (see e.g. [39, Proposition 12.15]), we have

$$\begin{aligned} \liminf _{t \rightarrow 0} \textrm{Per}(E_t, \Omega ) \ge \textrm{Per}(E, \Omega ). \end{aligned}$$

We prove the opposite inequality. We set for convenience $\mu _{E_t}:=\mathcal {H}^{d-1} \llcorner \partial E_t$. It is known (see e.g [39, (17.6), (17.29), (17.30)]) that

2.35 $$\begin{aligned} (g_t)_{\#} \mu _{E_t} = p_t \mu _E, \quad \text {with} \quad \Vert {p_t} \Vert _{C^0(\partial E)} = 1 + O(t). \end{aligned}$$

Combining this fact with the property that $\Omega \subset g_t^{-1}(\Omega )$ we obtain

$$\begin{aligned} \mu _{E_t} (\Omega ) \le \mu _{E_t} (g_t^{-1}(\Omega )) = (g_t)_{\#} \mu _{E_t} (\Omega ) = \mu _{E} (\Omega ) + O(t). \end{aligned}$$

Then, (*A*2) follows by taking the limsup as *t* goes to zero in the previous inequality.

Proof of  (*A*3) and (*A*5). It was proved by Schätzle (see [50]) that the Willmore functional is lower semicontinuous with respect to the $L^1$-convergence of $C^2$ sets, that is

2.36 $$\begin{aligned} \liminf _{t \rightarrow 0} \mathcal {W}(\partial E_t, \Omega ) \ge \mathcal {W}(\partial E, \Omega ). \end{aligned}$$

In our case, there are simpler and more direct ways to show the lower semicontinuity property above. For example, taking into account (*A*2), then (2.36) follows by an application of Reshetnyak’s continuity theorem, see [4, Remark 2] and also [38, Lemma 2] for a particular case. At this point, we claim that

2.37 $$\begin{aligned} H_{\partial E_t}(y) = H_{\partial E}(g_t(y)) + O(t), \qquad \forall y \in \partial E_t, \end{aligned}$$

where the reminder term is uniform with respect to $y \in \partial E_t$. Thus, the sets $\{E_t\}_{t \in (0, t_1] }$ satisfy (*A*5) provided that $t_1$ is small enough. By (2.35), (2.37) and since $\Omega \subset g_t^{-1}(\Omega )$, we have

$$\begin{aligned} \mathcal {W}(\partial E_t, \Omega ) \le \mathcal {W}(\partial E_t, \Omega ) + O(t) \end{aligned}$$

and the conclusion follows taking the limsup as *t* goes to zero in the inequality above. To check (2.37), let $\psi \in C^2(\mathbb {R}^d)$ such that $E = \{ x :\psi (x) > 0 \}$ and $\nabla \psi (x) \ne 0$ for any $x \in \partial E$. It is well known that

2.38 $$\begin{aligned} N_{\partial E} = \frac{\nabla \psi }{\left| \nabla \psi \right| } \quad \hbox { on}\ \{\psi = 0\}, \end{aligned}$$

2.39 $$\begin{aligned} H_{\partial E} = - \textrm{div} \bigg ( \frac{\nabla \psi }{\left| \nabla \psi \right| } \bigg ) = - \frac{\Delta \psi }{\left| \nabla \psi \right| } + \frac{\nabla ^2 \psi [\nabla \psi , \nabla \psi ]}{\left| \nabla \psi \right| ^3} \quad \hbox { on}\ \{\psi = 0\}, \end{aligned}$$

where $N_{\partial E}$ denotes the inner unit normal to *E*. In particular, the right-hand sides in the previous identities do not depend on the particular choice of $\psi$. For any $t \in (0,t_1]$, we define $\psi _t \in C^2(\mathbb {R}^d)$ by $\psi _t:= \psi \circ g_t$. It is clear that $E_t = \{ y :\psi _t(y) > 0 \}$ and that $\nabla \psi _t (y) \ne 0$ for any $y \in \partial E_t$. We set $G_t(y):= \nabla g_t(y)$ and we denote by $G_t^*(y)$ its transpose matrix. A direct computation shows

2.40 $$\begin{aligned} \nabla \psi _t(y) = G_t^*(y) \nabla \psi (g_t(y)), \end{aligned}$$

2.41 $$\begin{aligned} \nabla ^2 \psi _t(g_t(y)) = G_t^*(y) \nabla ^2 \psi (g_t(y)) G_t(y) + \langle \nabla \psi (g_t(y)), \nabla ^2 g_t (y) \rangle , \end{aligned}$$

where $\nabla ^2 g_t$ is the vector valued Hessian of $g_t$. Combining (2.39)–(2.41) and the fact that $\Vert {g_t - \textrm{Id}} \Vert _{C^2(\mathbb {R}^d)} \rightarrow 0$ as $t \rightarrow 0$ we deduce (2.37).

Proof of (*A*4). For any $t_2 \in (0,t_1]$, we define the set

$$\begin{aligned} \mathcal {N}(t_2):=\{ t \in (0,t_2] :\mathcal {H}^{d-1}(\partial E_t \cap \partial \Omega ) > 0 \}, \end{aligned}$$

and we claim that there exists $t_2 \in (0,t_1]$ such that $\mathcal {N}(t_2)$ is at most countable. If this is the case, then we find a sequence of positive real numbers $\{ t_j \}_{j \in \mathbb {N}}$ converging to zero for which the corresponding sets satisfy a stronger property than (*A*4), namely $\mathcal {H}^{d-1}(\partial E_{t_j} \cap \partial \Omega ) = 0$.

We write $\partial E_t \cap \partial \Omega = A_t \cup B_t$, where

$$\begin{aligned} A_t&:= \{ z \in \partial E_t \cap \partial \Omega :T_z \partial E_t = T_z \partial \Omega \} = \{ z \in \partial E_t \cap \partial \Omega :\left| \langle N_{\partial E_t}(z) , N_{\partial \Omega }(z) \rangle \right| = 1 \}, \\ B_t&:= \{ z \in \partial E_t \cap \partial \Omega :T_z \partial E_t \ne T_z \partial \Omega \} = \{ z \in \partial E_t \cap \partial \Omega :\left| \langle N_{\partial E_t}(z) , N_{\partial \Omega }(z) \rangle \right| < 1 \}, \end{aligned}$$

where $N_{\partial E_t}$ denotes the inner unit normal to $E_t$. It is well known (see e.g. [30, Section 1, Theorem 3.3]) that the transverse intersection of two submanifolds of codimensions $k_1$ and $k_2$ is either empty or a submanifold of codimension $k_1+k_2$. Therefore, for any $t \in (0,t_1]$, $B_t$ is either empty or a $(d-2)$-dimensional submanifold of $\mathbb {R}^d$. In both cases, we have that $\mathcal {H}^{d-1}(B_t)=0$. We prove that there exists $t_2 \in (0,t_1]$ such that for any $t,s \in (0, t_2]$ with $t \ne s$ we have $A_t \cap A_s = \emptyset$. Suppose by contradiction that there exist two sequences of positive real numbers $0< s_n < t_n$, with $t_n \rightarrow 0$ as $n \rightarrow \infty$, and two sequences of points $\{ x_n \}, \{ y_n \} \subset \partial E$ such that $f_{t_n}(x_n) = f_{s_n}(y_n) \in \partial \Omega$. Since $\partial E$ is compact, up to subsequences, we may assume that $x_n \rightarrow z_{\infty } \in \partial E$. Since $x_n = f_{t_n}(x_n) - t_n X(x_n)$, it follows that $z_n:=f_{t_n}(x_n) \rightarrow z_{\infty }$ and we infer that $z_{\infty } \in \partial \Omega$. Moreover, we have

$$\begin{aligned} \left| x_n - y_n\right| = \left| t_n X(x_n) - s_n X(y_n)\right| \le 2 t_n \max \{ \left| X(x)\right| :x \in \partial E \}, \end{aligned}$$

therefore $y_n \rightarrow z_\infty$. We claim that $z_{\infty } \in A_0$. Since $z_n=f_{t_n}(x_n) \in A_{t_n}$ for any $n \in \mathbb {N}$, we have

2.42 $$\begin{aligned} \left| \langle N_{\partial E_t}(z_n), N_{\partial \Omega }(z_n) \rangle \right| = 1. \end{aligned}$$

On the other hand, from (2.38) and (2.40) we have

$$\begin{aligned} N_{\partial E_t}(z) = \frac{G_t^*(z)N_{\partial E}(g_t(z))}{\left| G_t^*(z)N_{\partial E}(g_t(z))\right| }, \qquad \forall z \in \partial E_t. \end{aligned}$$

Therefore, we have $N_{\partial E_t}(z_n) \rightarrow N_{\partial E}(z_{\infty })$ as $n \rightarrow \infty$, since $z_n \rightarrow z_{\infty }$ and $g_t$ converges in $C^1$ to the identity map as $t \rightarrow 0$. Then, by (2.42) we derive

$$\begin{aligned} \left| \langle N_{\partial E}(z_{\infty }), N_{\partial \Omega }(z_{\infty }) \rangle \right| = \lim _{n \rightarrow \infty } \left| \langle N_{\partial E_t}(z_n), N_{\partial \Omega }(z_n) \rangle \right| = 1. \end{aligned}$$

This proves that $z_{\infty } \in A_0 = \{ z \in \partial E \cap \partial \Omega :\left| \langle N_{\partial E}(z), N_{\partial \Omega }(z) \rangle \right| = 1 \}$.

At this point, we claim that the map $F :\partial E \times \mathbb {R}\rightarrow \mathbb {R}^d$ defined as $F(x,t):=f_t(x)$ is a local diffeomorphism around $(z_{\infty },0)$. If this is the case, then we find a contradiction since, for *n* large, $F(x_n, t_n)=F(y_n,s_n)$ implies that $(x_n, t_n)=(y_n,s_n)$, but $s_n < t_n$. To prove the claim we have to check that the differential of *F* at the point $(z_{\infty },0)$ is surjective. It is not difficult to see that the image of the differential at $(z_\infty , 0)$ is given by

$$\begin{aligned} V:=T_{z_{\infty }} \partial E \oplus \textrm{Span} (X(z_{\infty })). \end{aligned}$$

Now, $V=\mathbb {R}^d$ if and only if $\langle X(z_{\infty }), N_{\partial E}(z_{\infty }) \rangle \ne 0$. Since $z_{\infty } \in A_0$ we have $\left| \langle X(z_{\infty }), N_{\partial E}(z_{\infty }) \rangle \right| = \left| \langle X(z_{\infty }), N_{\partial \Omega }(z_{\infty }) \rangle \right|$ and the latter is different from zero because of (2.34).

Building smooth sets. To summarize, we have constructed a sequence $\{ E_j \}_{j \in \mathbb {N}}$ of bounded open sets of class $C^2$ satisfying (*A*1), (*A*2), (*A*3), (*A*5) and the additional property $\mathcal {H}^{d-1}(\partial E_j \cap \partial \Omega ) = 0$ for any $j \in \mathbb {N}$. To conclude, we want to pass from $C^2$ to $C^\infty$ sets. For any $j \in \mathbb {N}$, there exists a sequence of smooth bounded open sets $E_{k,j}$ converging to $E_j$ in $L^1(\mathbb {R}^d)$, as $k \rightarrow \infty$, and such that

2.43 $$\begin{aligned} \lim _{k \rightarrow \infty } \textrm{Per}(E_{k,j}, \mathbb {R}^d) = \textrm{Per}(E_j, \mathbb {R}^d) \quad \text {and} \quad \lim _{k \rightarrow \infty } \mathcal {W}(\partial E_{k,j}, \mathbb {R}^d) = \mathcal {W}(\partial E_j, \mathbb {R}^d). \end{aligned}$$

Moreover, the sequence $\{E_{j,k}\}_{k\in \mathbb {N}}$ can be chosen such that (*A*5) is satisfied, with $H_{\partial E_j}$ at the right-hand side. We refer e.g. to [6] for a rigorous proof of this fact and more general results about the approximation by smooth sets on the whole Euclidean space. By $\mathcal {H}^{d-1}(\partial E_j \cap \partial \Omega ) = 0$, (2.43) and the lower semicontinuity of the Perimeter and the Willmore functional on $\Omega$ and on $\mathbb {R}^d \setminus {\overline{\Omega }}$, we infer

2.44 $$\begin{aligned} \lim _{k \rightarrow \infty } \textrm{Per}(E_{k,j}, \Omega ) = \textrm{Per}(E_j, \Omega ) \quad \text {and} \quad \lim _{k \rightarrow \infty } \mathcal {W}(\partial E_{k,j}, \Omega ) = \mathcal {W}(\partial E_j, \Omega ). \end{aligned}$$

Moreover, by the upper semicontinuity of the evaluation on closed sets with respect to the weak convergence of measures we have

2.45 $$\begin{aligned} \limsup _{k \rightarrow \infty } \mathcal {H}^{d-1}(\partial E_{k,j} \cap \partial \Omega ) \le \mathcal {H}^{d-1}(\partial E_j \cap \partial \Omega ) = 0. \end{aligned}$$

The conclusion follows from (2.44), (2.45) and a diagonal argument. $\square$

## On the decay of optimal profile

In this section, we discuss the proof of Theorem 1.2. Since $w'$ solves the fractional Allen–Cahn equation, then for any $\lambda > 0$ we have that

$$\begin{aligned} ((-\Delta )^s + \lambda ) w' = (\lambda - W''(w)) w', \end{aligned}$$

The proof of Theorem 1.2 relies on the decay properties of the fundamental solution $G_{s,\lambda }$ of the operator $\lambda + (-\Delta )^s$, whose symbol is $\lambda + \left| 2\pi \xi \right| ^{2s}$. Since $G_{s,\lambda }$ is formally defined by

$$\begin{aligned} G_{s,\lambda }(x) = {\mathscr {F}}_1^{-1} \left( \left( \lambda + \left| 2 \pi \xi \right| ^{2s} \right) ^{-1}\right) (x) = \int _{\mathbb {R}} \frac{e^{2\pi ix \xi }}{ \lambda + \left| 2\pi \xi \right| ^{2s} } \, d\xi , \end{aligned}$$

and for any $\xi \in \mathbb {R}$ we have

$$\begin{aligned} \frac{1}{ \lambda + \left| 2\pi \xi \right| ^{2s} } = \int _0^{+\infty } e^{-\lambda t} \exp \left( - t \left| 2 \pi \xi \right| ^{2s} \right) \, dt, \end{aligned}$$

then $G_{s,\lambda }$ formally satisfies

$$\begin{aligned} G_{s,\lambda }(x) = \int _{\mathbb {R}} e^{2\pi i x \xi } \int _0^{+\infty } e^{-\lambda t}\exp \left( - t \left| 2\pi \xi \right| ^{2s}\right) \, dt \, d \xi = \int _{0}^{+\infty } e^{-\lambda t} P^{(s)}_1 (t, x) \, dt. \end{aligned}$$

The above computation can be made rigorous.

### Proposition 3.1

Fix $s \in (0,1)$ and $\lambda >0$. For any $x \in \mathbb {R}$, set

3.1 $$\begin{aligned} G_{s,\lambda }(x): = \int _{0}^{+\infty } e^{-\lambda t} P^{(s)}_1(t,x) \, dt. \end{aligned}$$

Then, $G_{s,\lambda } \in L^1(\mathbb {R})$ and it holds that

3.2 $$\begin{aligned} {\mathscr {F}}_1(G_{s,\lambda })(\xi ) = \frac{1}{ \lambda + \left| 2\pi \xi \right| ^{2s} } \qquad \forall \xi \in \mathbb {R}. \end{aligned}$$

Moreover, $G_{s,\lambda } \in C^{\infty }(\mathbb {R}{\setminus } \{0\})$ and for any $k \ge 0$ it holds that

3.3 $$\begin{aligned} \left| \partial _x^k G_{s,\lambda } (x)\right| \le C(s,\lambda , k) \left| x\right| ^{-k-1-2s} \qquad \forall x\ne 0. \end{aligned}$$

### Proof

To begin, since $P^{(s)}_1(t, x) >0$ for any $(t,x) \in (0, +\infty ) \times \mathbb {R}$, we notice that $G_{s,\lambda }(x)$ is always well defined with values in $[0, +\infty ]$. Then, by (2.6) and Fubini’s theorem, it results that

$$\begin{aligned} \int _{\mathbb {R}} G_{s,\lambda }(x)&= \int _0^{+\infty } \int _{\mathbb {R}} e^{-\lambda t} t^{-\frac{1}{2s}} P^{(s)}_1\left( 1, t^{-\frac{1}{2s}}x\right) \, dx \, dt \\&= \int _0^{+\infty } e^{-\lambda t} \int _{\mathbb {R}} P^{(s)}_{1} (1, y)\, dy \, dt < +\infty . \end{aligned}$$

From now on, we neglect constants $C(k,s,\lambda )>0$. Moreover, by Proposition 2.4, we have that

$$\begin{aligned} G_{s,\lambda }(x) \lesssim \int _{0}^{+\infty } e^{-\lambda t} t^{-\frac{1}{2s}} \frac{1}{1+ \left| x\right| ^{2s+1} t^{-\frac{2s+1}{2s}} }\, dt \lesssim \left| x\right| ^{-1-2s}\int _{0}^{+\infty } e^{-\lambda t} t\, dt \lesssim \left| x\right| ^{-1-2s}, \end{aligned}$$

thus proving (3.3) for $k = 0$. To compute the Fourier transform, by Fubini’s theorem and the inversion formula in $L^2$, we have that

$$\begin{aligned} {\mathscr {F}}_1( G_{s,\lambda })(\xi )&= \int _0^{+\infty } e^{-\lambda t} {\mathscr {F}}_1 \left( P_1^{(s)}(t, \cdot )\right) (\xi ) \, dt \\  &= \int _{0}^{+\infty } e^{-\lambda t} \exp \left( - \left| 2\pi \xi \right| ^{2s} t \right) \, dt = \frac{1}{\lambda + \left| 2 \pi \xi \right| ^{2s} }, \end{aligned}$$

thus proving (3.2). To prove that $G_{s,\lambda }$ is smooth away from the origin, we check that

3.4 $$\begin{aligned} \int _0^{+\infty } e^{-\lambda t} \left| \partial _x^k P^{(s)}_1(t,x)\right| \, dt \lesssim \left| x\right| ^{-k-1-2s} \qquad \forall k\in \mathbb {N}\qquad \forall x \ne 0. \end{aligned}$$

Indeed, by (2.6) and Proposition 2.5 we have

$$\begin{aligned} \int _0^{+\infty } e^{-\lambda t} \left| \partial _x^k P^{(s)}_1(t,x)\right| \, dt&= \int _0^{+\infty } e^{-\lambda t} t^{-\frac{k+1}{2s}} \left| \partial _x^k P^{(s)}_1(1, t^{-\frac{1}{2s}} x) \right| \, dt \\  &\lesssim \int _0^{+\infty } e^{-\lambda t} t^{-\frac{k+1}{2s}} \frac{1}{1+ t^{-\frac{k+1+2s}{2s}} \left| x\right| ^{-k-1-2s}} \, dt \\  &\lesssim \left| x\right| ^{-k-1-2s} \int _0^{+\infty } e^{-\lambda t} t \, dt, \end{aligned}$$

thus proving (3.4). Then, it is easy to check that $G_{s,\lambda }$ is smooth away from the origin and it holds

$$\begin{aligned} \partial ^k_x G_{s,\lambda }(x) = \int _0^{+\infty } e^{-\lambda t} \partial _x^k P^{(1)}_s(t,x) \, dt. \end{aligned}$$

Thus, (3.3) follows by (3.4). $\square$

The proof of Theorem 1.2 follows from Proposition 3.1.

### Proof of Theorem 1.2

We perform the proof by induction. We start with the case $k=2$. We neglect constants $C(k,s, W)>0$. Since $w'$ solves (3.5), iterating the estimates in [53, Proposition 2.8$-$2.9], it is readily checked that $w \in W^{2, \infty }(\mathbb {R})$ with $\Vert {w} \Vert _{W^{2,\infty }(\mathbb {R})} \lesssim 1$. Letting $\lambda = W''(\pm 1)$, since $w' \in L^\infty (\mathbb {R})$ solves

3.5 $$\begin{aligned} (\lambda + (-\Delta )^s) w' = (\lambda - W''(w)) w', \end{aligned}$$

then by (3.2) we have

3.6 $$\begin{aligned} w'(x) = \int _{\mathbb {R}} G_{s,\lambda }(x-z) \Psi (z) w'(z) \, dz, \end{aligned}$$

where $G_{s,\lambda }$ is given by (3.1) and we set $\Psi (z) = \lambda - W''(w(z))$. Since $G_{s,\lambda }$ is smooth away from 0 (see Proposition 3.1), for any $x > 1$ we have that

$$\begin{aligned} w''(x)&= \int _{\left| x-z\right| \ge \frac{x}{2}} G_{s,\lambda } (x-z) \partial _z [ \Psi (z) w'(z) ] \, dz + \int _{\left| x-z\right| < \frac{x}{2}} G_{s,\lambda } (x-z) \partial _z [ \Psi (z) w'(z) ] \, dz \\  &= \int _{|x-z|\ge \frac{x}{2}} \partial _x G_{s,\lambda } (x-z) \Psi (z) w'(z)\,dz -\Bigg [ G_{s,\lambda }(x-z)\Psi (z) w'(z) \Bigg ]_{\frac{x}{2}}^{\frac{3x}{2}} \\  &\qquad +\int _{\frac{x}{2}}^{\frac{3x}{2}} G_{s,\lambda } (x-z) \left[ -W'''(w(z))(w'(z))^2 + (\lambda -W''(w(z)))w''(z) \right] \, dz. \end{aligned}$$

By the decay properties of $G_{s,\lambda }, \partial _x G_{s,\lambda }$ (see Proposition 3.1) and $w \in W^{2,\infty }(\mathbb {R})$, we infer that

$$\begin{aligned} \left| w''(x)\right| \lesssim \left| x\right| ^{-2-2s} + \left| x\right| ^{-2s} \Vert {w''} \Vert _{L^\infty ([{x}/{2},{3x}/{2}])}. \end{aligned}$$

Then, letting $h > 1+ {1}/{s}$ be an integer and iterating *h* times the above estimate, for $x> 2^{h+1}$ we find that

$$\begin{aligned} \left| w''(x)\right| \lesssim \left| x\right| ^{-2-2s} + \left| x\right| ^{-2s h} \Vert {w''} \Vert _{L^\infty ([2^{-h}x,2^{h}x])} \lesssim \left| x\right| ^{-2-2s} \left( 1 + \Vert {w''} \Vert _{L^\infty ([1, +\infty ))}\right) , \end{aligned}$$

thus proving (1.11) for $x > 2^{h+1}$. The estimate for $x \le -2^{h+1}$ is analogous, since $w''$ is odd, and the case $x \in [-2^{h+1},2^{h+1}]$ is trivial, since $w'' \in L^\infty (\mathbb {R})$. Then, (1.11) is proved for $k=2$.

Fix $k \ge 2$ and assume that $W \in C^{k+2}(\mathbb {R})$, $w \in C^{k}(\mathbb {R})$ and (1.11) is proved for any derivative of order smaller than or equal to *k*. We prove that $w\in C^{k+1}(\mathbb {R})$ and (1.11) holds for $\partial _x^{k+1} w$. Differentiating *k* times (1.7), we find that

$$\begin{aligned} (-\Delta )^s \partial _x^{k} w = -\partial _x^{k} W'(w), \end{aligned}$$

where the right-hand side satisfies $\Vert {\partial _x^{k} W'(w) } \Vert _{L^\infty (\mathbb {R})} \lesssim 1$. Hence, iterating [53, Proposition 2.8$-$2.9], it is readily checked that $\partial _x^k w \in C^{1}(\mathbb {R})$ and it holds $\Vert {\partial _x^{k+1} w} \Vert _{L^\infty (\mathbb {R})} \lesssim 1$. Then, since $G_{s,\lambda }$ is smooth away from the origin (see Proposition 3.1), by (3.6) and integrating by parts *k* times, for $x>1$ we find that

$$\begin{aligned} \partial _x^{k+1} w(x)&= \int _{\left| x-z\right| \ge \frac{x}{2}} G_{s,\lambda }(x-z) \partial _z^k[\Psi (z) w'(z)] \, dz \\&+ \int _{\left| x-z\right|< \frac{x}{2}} G_{s,\lambda }(x-z) \partial _z^k[\Psi (z) w'(z)] \, dz \\  &= \int _{\left| x-z\right| \ge \frac{x}{2}} \partial _x^k G_s(x-z) \Psi (z) w'(z)\, dz \\&- \sum _{i=0}^{k-1} \bigg [ \partial _x^{i} G_{s,\lambda }(x-z) \partial ^{k-1-i}_z[\Psi (z) w'(z)] \bigg ]_{z=\frac{x}{2}}^{z= \frac{3x}{2}} \\  &\qquad + \int _{\left| x-z\right| < \frac{x}{2}} G_{s,\lambda }(x-z) \partial _z^k[\Psi (z) w'(z)] \, dz = A+ \sum _{i=0}^{k-1} B_i + C. \end{aligned}$$

We estimate separately each term. To begin, by Proposition 3.1, we have that

$$\begin{aligned} \left| A\right| \lesssim \left| x\right| ^{-k-1-2s} \Vert {\Psi w'} \Vert _{L^1(\mathbb {R})} \lesssim \left| x\right| ^{-k-1-2s}. \end{aligned}$$

For any $j = 0, \dots , k$, by the chain rule and since (1.11) holds up to the order *k*, it is easy to estimate

3.7 $$\begin{aligned} \left| \partial _z^j \Psi (z)\right| \lesssim \left| z\right| ^{-j-2s}. \end{aligned}$$

Then, fix an index $i = 0, \dots , k-1$. By Leibniz rule, we have that

3.8 $$\begin{aligned} \left| \partial ^i_z [\Psi (z) w'(z)]\right|&\le \sum _{j=0}^i \left( {\begin{array}{c}i\\ j\end{array}}\right) \left| \partial _z^j\Psi (z)\right| \left| \partial _z^{i+1-j} w(z)\right| \lesssim \left| z\right| ^{-i-1-4s}. \end{aligned}$$

Therefore, by Proposition 3.1 and (3.8), we infer that

$$\begin{aligned} \sum _{i=0}^{k-1} \left| B_i\right| \lesssim \left| x\right| ^{-k-1-6s}. \end{aligned}$$

Lastly, since $G_{s,\lambda } \in L^1(\mathbb {R})$, using (1.11) up to order *k* and by (3.7), we estimate

$$\begin{aligned} \left| C\right|&\lesssim \sum _{j=1}^{k-1} \Vert {(\partial _z^j \Psi ) ( \partial _z^{k-j+1} w ) } \Vert _{L^\infty ([{x}/{2}, {3x}/{2}])} + \Vert { \Psi \partial _z^{k+1} w } \Vert _{L^\infty ([{x}/{2}, {3x}/{2}])} \\  &\lesssim \left| x\right| ^{-k-1-4s} + \left| x\right| ^{-2s} \Vert { \partial _z^{k+1} w } \Vert _{L^\infty ([{x}/{2}, {3x}/{2}])}. \end{aligned}$$

To summarize, for $x>1$ we have that

$$\begin{aligned} \left| \partial _x^{k+1} w(x)\right| \lesssim \left| x\right| ^{-k-1-2s} + \left| x\right| ^{-2s} \Vert { \partial _z^{k+1} w } \Vert _{L^\infty ([{x}/{2}, {3x}/{2}])}. \end{aligned}$$

Then, letting $h > 1+ {(k+1)}/{2s}$ be an integer and iterating *h* times the above estimate, for $x> 2^{h+1}$ we find that

$$\begin{aligned} \left| \partial _x^{k+1 }w(x)\right| \lesssim \left| x\right| ^{-k-1-2s} + \left| x\right| ^{-2s h} \Vert {\partial _x^{k+1 }w} \Vert _{L^\infty ([2^{-h}x,2^{h}x])}\\ \lesssim \left| x\right| ^{-k-1-2s} \left( 1 + \Vert {\partial _x^{k+1 }w} \Vert _{L^\infty ([1, +\infty ))}\right) , \end{aligned}$$

thus proving (1.11) for $x > 2^{h+1}$ for the derivative of order $k+1$. The estimate for $x \le -2^{h+1}$ is analogous, since $\partial _x^{k+1} w$ is odd or even, and the case $x \in [-2^{h+1},2^{h+1}]$ is trivial, since $\partial _x^{k+1} w$ is uniformly bounded. Then, the proof is concluded. $\square$

### Remark 3.2

Using [53, Proposition 2.8$-$2.9] as in the proof of Theorem 1.2, assuming $W \in C^{k,\alpha }_{\textrm{loc}}$ with $\alpha + 2\,s >1$ would still suffice to prove that $w \in C^{k, \beta }(\mathbb {R})$ for some $\beta >0$. However, the main purpose of Theorem 1.2 is to study the decay rate of the $\partial _x^k w$. Since an integration by part is needed in our argument to prove (1.11), we have to assume $W \in C^{k+1}$.

As a corollary, we obtain the following result.

### Corollary 3.3

Fix $s \in (0,1)$. Let *E* be a bounded open set of class $C^2$ according to Definition 2.6 and let $\delta >0$ be given by Lemma 2.9. Let $u_\varepsilon$ be defined by (4.1). Then, for any $\Lambda \ge 1$ it holds

$$\begin{aligned} \sup _{\varepsilon \in (0,1)} \Vert {(-\Delta )^s u_\varepsilon } \Vert _{L^\infty (\mathbb {R}^d \setminus \Sigma _{{\delta }/{\Lambda }})} \le C(d,s,W,\delta , \Lambda ). \end{aligned}$$

### Proof

We neglect multiplicative constants $C(d,s,\delta ,W, \Lambda )>0$. By Lemma 2.1, we have

$$\begin{aligned} \sup _{\varepsilon \in (0,1)} \Vert {(-\Delta )^s u_\varepsilon } \Vert _{L^\infty (\mathbb {R}^d \setminus \Sigma _{{\delta }/{\Lambda }})} \lesssim \Vert {u_\varepsilon } \Vert _{L^\infty (\mathbb {R}^d)} + \Vert {u_\varepsilon } \Vert _{C^2( \mathbb {R}^d \setminus \Sigma _{{\delta }/{2\Lambda }} )}. \end{aligned}$$

Since $\Vert {u_\varepsilon } \Vert _{L^\infty (\mathbb {R}^d)} = 1$ for any $\varepsilon$, it remains to estimate $u_\varepsilon$ in $C^2(\mathbb {R}^d \setminus \Sigma _{{\delta }/{2\Lambda }} )$. Recalling that $\beta _{\Sigma } \in C^2(\mathbb {R}^d)$ and $\left| \beta _\Sigma (x)\right| \ge {\delta }/{2\Lambda }$ for any $x \in \mathbb {R}^d \setminus \Sigma _{{\delta }/{2\Lambda }}$ (see Definition 2.10), we need to estimate $w_\varepsilon (t) = w\left( {t}/{\varepsilon }\right)$ in $C^2( \{ \left| t\right| > {\delta }/{2\Lambda } \})$ for $\varepsilon \in (0,1)$. Indeed, by Theorem 1.2 we have

$$\begin{aligned} \left| w'_\varepsilon (t)\right| + \left| w''_{\varepsilon }(t)\right| = \left| \frac{1}{\varepsilon } w'\left( \frac{t}{\varepsilon } \right) \right| + \left| \frac{1}{\varepsilon ^2} w''\left( \frac{t}{\varepsilon }\right) \right| \lesssim \varepsilon ^{2s} \qquad \forall \left| t\right| \ge \frac{\delta }{2\Lambda } \qquad \forall \varepsilon \in (0,1). \end{aligned}$$

$$\square $$

## Expansion of the fractional Laplacian around the boundary

As we explained in the introduction, the expansion of the fractional Laplacian in Fermi coordinates for the function defined by (4.1) is crucial for proving our main result. In this section, we provide a complete proof of this expansion. We emphasize that our approach closely follows some computations from [14, Section 3], which deal with a three-dimensional setting. However, we adapt these computations to our framework, carefully keeping track of constants and error terms.

### Theorem 4.1

Let *W* be a double-well potential satisfying (*W*1), (*W*2), (*W*3). Let $w: \mathbb {R}\rightarrow (-1,1)$ be the one-dimensional optimal profile and for any $\varepsilon >0$ let us set $w_\varepsilon (z) = w \left( {z}/{\varepsilon }\right)$. Assume that $s \in \left( {1}/{2},1\right)$. Let *E* be a bounded open set of class $C^3$ and let $\Sigma = \partial E$. Let $\delta >0$ be such that $\beta _\Sigma$ is well defined according to Definition 2.10 and let us set

4.1 $$\begin{aligned} u_\varepsilon (x) = w_\varepsilon (\beta _\Sigma (x)). \end{aligned}$$

There exists $\Lambda _0, C \ge 1$ depending on $\Sigma$ with the following property. For any $\Lambda \ge \Lambda _0$ for any $\varepsilon \in (0,1)$ there exists $\mathcal {R}_{\varepsilon ,\Lambda }: \Sigma _{{\delta }/{10 \Lambda }} \rightarrow \mathbb {R}$ such that for any $x_0 \in \Sigma _{{\delta }/{10 \Lambda }}$ it holds

4.2 $$\begin{aligned} (-\Delta )^s u_\varepsilon (x_0)&= (-\partial _{zz})^s w_\varepsilon (z_0) + \frac{\gamma _{1,s}}{2} \frac{H_{\Sigma }(x_0')}{(2s-1)} \int _{-{\delta }/{\Lambda }}^{{\delta }/{\Lambda }} \frac{w_\varepsilon '(z_0+{\bar{z}})}{\left| {\bar{z}}\right| ^{2s-1}} \, d{\bar{z}} + \mathcal {R}_{\varepsilon , \Lambda }(x_0), \end{aligned}$$

where we set $z_0 = \textrm{dist}_{\Sigma }(x_0)$ and $x_0' = \pi _{\Sigma } (x_0)$. Moreover, it holds that

4.3 $$\begin{aligned} \Vert {\mathcal {R}_{\varepsilon ,\Lambda }} \Vert _{L^\infty (\Sigma _{{\delta }/{10 \Lambda }})} \le C \Lambda ^{2s}. \end{aligned}$$

### Proof

For the reader convenience, we split the proof in several steps. To begin, we summarize the strategy adopted. Let $\Lambda _0$ be the geometric constant given by Lemma 2.17 and fix $\Lambda \ge \Lambda _0$. Then, we take $x_0 \in \Sigma _{{\delta }/{10 \Lambda }}$ and we denote by $x_0' = \pi _{\Sigma }(x_0), z_0 = \textrm{dist}_\Sigma (x_0)$. Then, with the same notation as Lemma 2.17 we take Fermi coordinates around $x_0'$, i.e. $\Phi : B_\delta ^{d-1} \times (-\delta , \delta ) \rightarrow \mathbb {R}^d$. We recall that $x_0 = \Phi (0, z_0)$ with respect to this coordinate system. For convenience, we set $\mathcal {T}_{{\delta }/{\Lambda }}(x_0): = \Phi (\mathcal {B}_{{\delta }/{\Lambda }}(z_0))$, where $\mathcal {B}_{{\delta }/{\Lambda }}(z_0) \subset B_\delta ^{d-1} \times (-\delta , \delta )$ is defined by (2.27). Since the fractional Laplacian is a singular integral (see (2.1)) and $\mathcal {T}_{{\delta }/{\Lambda }}(x_0)$ is an open set around $x_0$ (see Lemma 2.17), we analyse separately the contribution in $\mathcal {T}_{{\delta }/{\Lambda }}(x_0)$ and in $\mathbb {R}^d \setminus \mathcal {T}_{{\delta }/{\Lambda }}(x_0)$. More precisely, we have

$$\begin{aligned} (-\Delta )^s u_\varepsilon (x_0)&= \gamma _{d,s} \int _{\mathbb {R}^d \setminus \mathcal {T}_{{\delta }/{\Lambda }}(x_0)} \frac{u_\varepsilon (x_0) - u_\varepsilon (x)}{\left| x-x_0\right| ^{d+2s}} \, dx \\&+ \lim _{\nu \rightarrow 0} \gamma _{d,s} \int _{\mathcal {T}_{{\delta }/{\Lambda }}(x_0) \setminus B_\nu (x_0)} \frac{u_\varepsilon (x_0) - u_\varepsilon (x)}{\left| x-x_0\right| ^{d+2s}} \, dx. \end{aligned}$$

Unless otherwise specified, the reminders involved in the following computations satisfy bounds depending on $\Sigma$. In particular, they are independent on the choice of the point $x_0$. This fact follows essentially by Remark 2.12, Lemmas 2.15 and 2.17.

Step 1: Estimating the outer contribution. Up to an implicit constant depending only on $d, \delta , \Sigma$, by Lemma 2.17 we estimate the integral in $\mathbb {R}^d \setminus \mathcal {T}_{{\delta }/{\Lambda }}(x_0)$ as follows

4.4 $$\begin{aligned} \left| \int _{\mathbb {R}^d \setminus \mathcal {T}_{{\delta }/{\Lambda }}(x_0)} \frac{u_\varepsilon (x_0)-u_\varepsilon (x)}{\left| x-x_0\right| ^{d+2s}} \, dx \right|&\lesssim \int _{\mathbb {R}^d \setminus B_{{\delta }/{10 \Lambda }}(x_0) } \frac{1}{\left| x-x_0\right| ^{d+2s}} \, dx \lesssim \Lambda ^{2s}. \end{aligned}$$

Step 2: Rewriting the inner contribution. The estimate of the singular term is much more delicate and it requires a careful analysis. To ease the notation, given $\nu >0$, we set

4.5 $$\begin{aligned} \Delta ^s_{\nu } u_\varepsilon (x_0):= \int _{\mathcal {T}_{{\delta }/{\Lambda }} (x_0) \setminus B_\nu (x_0)} \frac{u_\varepsilon (x) - u_\varepsilon (x_0)}{\left| x-x_0\right| ^{d+2s}} \, dx. \end{aligned}$$

We changed sign to avoid many negative terms in the computations. We aim to write the integral in (4.5) as an integral with respect to the variables *z*, *y*. Furthermore, writing the Euclidean distance as in Remark 2.16, by definition of $\mathcal {T}_{{\delta }/{\Lambda } } (x_0)$ and recalling that $\Phi$ is a diffeomorphism by Lemma 2.17, we have

$$\begin{aligned} \mathcal {T}_{{\delta }/{\Lambda }}(x_0) \setminus B_{\nu }(x_0) = \Phi (\mathcal {U}^1_{\nu }), \end{aligned}$$

where we set

$$\begin{aligned} \mathcal {U}^1_{\nu }:= \left\{ (y,z) \in \mathcal {B}_{{\delta }/{\Lambda }} (z_0) :\begin{aligned} \left| z-z_0 + Y(y)\cdot N(y) + z_0(1-N_d(y))\right| ^2 + \left| (Y(y) - z_0 e_d)_\tau \right| ^2 \ge \nu ^2 \end{aligned} \right\} . \end{aligned}$$

Here $\mathcal {B}_{{\delta }/{\Lambda }}(z_0)$ is defined by (2.27). Since $\beta _\Sigma$ is the proper distance from the boundary (see Definition 2.10 and Lemma 2.17) in $\Phi (\mathcal {U}^1_{\nu })$, changing variables in (4.5) and recalling that $u_\varepsilon$ is given by (4.1), we have that

$$\begin{aligned} \Delta ^s_{\nu } u_\varepsilon (x_0) = \int _{\mathcal {U}^1_{\nu }} \frac{(w_\varepsilon (z)-w_\varepsilon (z_0)) \left| \det (\nabla \Phi (y,z))\right| }{\left( \left| z-z_0 + Y(y)\cdot N(y) + z_0(1-N_d(y))\right| ^2 + \left| (Y(y) - z_0 e_d)_\tau \right| ^2\right) ^{\frac{d+2s}{2}}} \, dz \, dy. \end{aligned}$$

Next, with the notation of Lemma 2.17, for any $y\in {\mathscr {B}}_{{\delta }/{\Lambda }}^{d-1}(z_0)$, we set

4.6 $$\begin{aligned} {\bar{z}}(y,z) = z-z_0 + Y(y)\cdot N(y) + z_0 (1- N_d(y)). \end{aligned}$$

Therefore, changing again variables, we have that

$$\begin{aligned} \Delta ^s_{\nu } u_\varepsilon (x_0) = \int _{\mathcal {U}^2_{\nu }} \frac{(w_\varepsilon (z(y, {\bar{z}}))-w_\varepsilon (z_0)) \left| \det (\nabla \Phi (y,z(y, {\bar{z}})))\right| }{\left( \left| {\bar{z}}\right| ^2 + \left| (Y(y) - z_0 e_d)_\tau \right| ^2\right) ^{\frac{d+2s}{2}}} \, d{\bar{z}} \, dy, \end{aligned}$$

where we set

$$\begin{aligned} \mathcal {U}^2_{\nu }:= \left\{ (y,{\bar{z}}) \in {\mathscr {B}}_{{\delta }/{\Lambda }}^{d-1}(z_0) \times \left( -{\delta }/{\Lambda }, {\delta }/{\Lambda }\right) :\left| {\bar{z}}\right| ^2 + \left| (Y(y) - z_0 e_d)_\tau \right| ^2 \ge \nu ^2 \right\} . \end{aligned}$$

Hence, setting

4.7 $$\begin{aligned} {\bar{y}}(y) = (\textrm{Id} - \nabla ^2 g(0) z_0) y, \end{aligned}$$

and changing again variables, we have that

4.8 $$\begin{aligned} \Delta ^s_{\nu } u_\varepsilon (x_0) = \int _{\mathcal {U}^3_{\nu }} \frac{(w_\varepsilon (z(y({\bar{y}}), {\bar{z}}))-w_\varepsilon (z_0)) }{\left( \left| {\bar{z}}\right| ^2 + \left| (Y(y({\bar{y}})) - z_0 e_d)_\tau \right| ^2\right) ^{\frac{d+2s}{2}}} \frac{\left| \det (\nabla \Phi (y({\bar{y}}),z(y({\bar{y}}), {\bar{z}})))\right| }{\left| \det (\textrm{Id} - z_0 \nabla ^2 g (0))\right| } \, d{\bar{z}} \, d{\bar{y}}, \end{aligned}$$

where we set

4.9 $$\begin{aligned} \mathcal {U}^3_{\nu }:= \left\{ ({\bar{y}},{\bar{z}}) \in B_{{\delta }/{\Lambda }}^{d-1} \times \left( -{\delta }/{\Lambda }, {\delta }/{\Lambda } \right) :\left| {\bar{z}}\right| ^2 + \left| (Y(y({\bar{y}})) - z_0 e_d)_\tau \right| ^2 \ge \nu ^2 \right\} . \end{aligned}$$

Step 3: Computing the leading order terms. In order to estimate the integral in (4.8), we compute the leading orders of the terms involved. By Definition 2.14, (4.7) and (2.24), we have

4.10 $$\begin{aligned} \left| (Y(y({\bar{y}})) - z_0 e_d)_\tau \right| ^2 = \left| {\bar{y}}\right| ^2 + O(\left| z_0\right| \left| {\bar{y}}\right| ^3) + O(\left| {\bar{y}}\right| ^4). \end{aligned}$$

By Lemma 2.15 and (4.7), it is clear that

4.11 $$\begin{aligned} Y(y({\bar{y}}))\cdot N(y({\bar{y}})) = -\frac{1}{2} \sum _{i=1}^{d-1} {\bar{y}}_i^2 k_i + O( \left| z_0\right| \left| {\bar{y}}\right| ^2 ), \end{aligned}$$

4.12 $$\begin{aligned} 1- N_d(y({\bar{y}})) = O(\left| {\bar{y}}\right| ^2), \end{aligned}$$

Thus, by (4.6), (4.7), (4.11) and (4.12), we infer that

4.13 $$\begin{aligned} z(y, {\bar{z}}) = z_0 + {\bar{z}} + \frac{1}{2} \sum _{i=1}^{d-1} y_i^2 k_i + O(\left| y\right| ^3) + O( \left| z_0\right| \left| y\right| ^2). \end{aligned}$$

4.14 $$\begin{aligned} z(y({\bar{y}}), {\bar{z}}) = z_0 + {\bar{z}} + f(z_0, {\bar{y}}), \end{aligned}$$

where we set

4.15 $$\begin{aligned} f(z_0, {\bar{y}}) = \sum _{i=1}^{d-1} \frac{1}{2} k_i {\bar{y}}_i^2 + O( \left| z_0\right| \left| {\bar{y}}\right| ^2) + O( \left| {\bar{y}}\right| ^3). \end{aligned}$$

By Lemma 2.15, (4.13) and (4.6), we have that

4.16 $$\begin{aligned}&\det \nabla \Phi (y, z(y, {\bar{z}})) \nonumber \\&= \prod _{i=1}^{d-1} (1- ({\bar{z}}+z_0 + O(\left| y\right| ^2)) k_i ) + O((\left| {\bar{z}}\right| + \left| z_0\right| + \left| y\right| ^2 ) \left| y\right| ) + O(\left| y\right| ^2) \nonumber \\  &= \prod _{i=1}^{d-1} (1- k_i (z_0 + {\bar{z}})) + O(\left| z_0\right| \left| y\right| ) + O(\left| {\bar{z}}\right| \left| y\right| ) + O (\left| y\right| ^2) . \end{aligned}$$

By (4.7) and (4.16), we have that

4.17 $$\begin{aligned}&\frac{\det \nabla \Phi (y({\bar{y}}), z(y({\bar{y}}) , {\bar{z}}))}{\det (\textrm{Id} - z_0 \nabla ^2 g (0))} \nonumber \\&= \left( \prod _{i=1}^{d-1} (1- z_0 k_i - {\bar{z}} k_i ) + O((\left| z_0\right| + \left| {\bar{z}}\right| )\left| {\bar{y}}\right| + \left| {\bar{y}}\right| ^2) \right) \left( \prod _{i=1}^{d-1} (1- z_0 k_i )\right) ^{-1} \nonumber \\  &= \prod _{i=1}^{d-1} \left( 1- {\bar{z}} \frac{k_i }{ 1- z_0 k_i } \right) + O((\left| z_0\right| + \left| {\bar{z}}\right| )\left| {\bar{y}}\right| + \left| {\bar{y}}\right| ^2) \nonumber \\  &= 1- {\bar{z}} H_{\Sigma }(x_0') + O(\left| z_0\right| \left| {\bar{y}}\right| ) + O(\left| {\bar{y}}\right| ^2) + O ( \left| {\bar{z}}\right| ^2). \end{aligned}$$

From now on, we denote by

$$\begin{aligned} \rho ^2 = \left| {\bar{z}}\right| ^2 + \left| {\bar{y}}\right| ^2. \end{aligned}$$

Hence, by (4.10), (4.14), (4.15), (4.17), we write (4.8) as follows

4.18 $$\begin{aligned} \Delta ^s_{\nu } u_\varepsilon (x_0) = \int _{\mathcal {U}^3_{\nu }} \frac{\left[ w_\varepsilon ( z_0 + {\bar{z}} + f(z_0, {\bar{y}}))-w_\varepsilon (z_0)\right] \left[ 1- {\bar{z}} H_{\Sigma }(x_0') + O(\left| z_0\right| \left| {\bar{y}}\right| ) + O( \rho ^2) \right] }{\left( \rho ^2 + O(\left| z_0\right| \left| {\bar{y}}\right| ^3) + O (\left| {\bar{y}}\right| ^4) ) \right) ^{\frac{d+2s}{2}}} \, d{\bar{z}} \, d{\bar{y}}. \end{aligned}$$

By standard manipulations, we write

$$\begin{aligned}&\frac{1- {\bar{z}} H_\Sigma (x_0') + O(\left| z_0\right| \left| {\bar{y}}\right| ) + O(\rho ^2) }{\left( \rho ^2 + O(\left| z_0\right| \left| {\bar{y}}\right| ^3) + O (\left| {\bar{y}}\right| ^4) ) \right) ^{\frac{d+2s}{2}}}\\&= \frac{1- {\bar{z}} H_\Sigma (x_0') + O(\left| z_0\right| \left| {\bar{y}} \right| ) + O(\rho ^2) }{ \rho ^{d+2s} } \left( 1 + O(\left| z_0\right| \left| {\bar{y}}\right| ) + O(\left| {\bar{y}}\right| ^2) \right) \\&= \frac{1- {\bar{z}} H_\Sigma (x_0') + O(\left| z_0\right| \left| {\bar{y}} \right| ) + O(\rho ^2) }{ \rho ^{d+2s} }. \end{aligned}$$

Hence, by (4.18) we write

$$\begin{aligned} \Delta ^s_{\nu } u_\varepsilon (x_0) = \int _{\mathcal {U}^3_{\nu }} \frac{ g_\varepsilon ({\bar{y}}, {\bar{z}}, z_0) }{\rho ^{d+2s}} \, d{\bar{z}} \, d{\bar{y}}, \end{aligned}$$

where we denote by

4.19 $$\begin{aligned} g_\varepsilon ({\bar{y}}, {\bar{z}}, z_0):= \left[ w_\varepsilon ( z_0 + {\bar{z}} + f(z_0, {\bar{y}}))-w_\varepsilon (z_0)\right] \left[ 1- {\bar{z}} H_{\Sigma }(x_0') + O(\left| z_0\right| \left| {\bar{y}}\right| ) + O(\rho ^2) \right] \nonumber \\ \end{aligned}$$

We claim that

4.20 $$\begin{aligned} \lim _{\nu \rightarrow 0} \int _{\mathcal {U}^3_{\nu }} \frac{g_\varepsilon ({\bar{y}}, {\bar{z}}, z_0)}{\rho ^{d+2s}} \, d{\bar{z}} \, d{\bar{y}} = \lim _{\nu \rightarrow 0} \int _{\mathcal {C}_{\nu }} \frac{g_\varepsilon ({\bar{y}}, {\bar{z}}, z_0)}{\rho ^{d+2s}} \, d{\bar{z}} \, d{\bar{y}}, \nonumber \\ \end{aligned}$$

where $\mathcal {C}_\nu$ is the complement of a ball in a cylinder

4.21 $$\begin{aligned} \mathcal {C}_{\nu }:= \left\{ ({\bar{y}},{\bar{z}}) \in B_{{\delta }/{\Lambda }}^{d-1} \times \left( -{\delta }/{\Lambda }, {\delta }/{\Lambda }\right) :\rho \ge \nu \right\} . \end{aligned}$$

To begin, we estimate the symmetric difference between $\mathcal {U}_\nu ^3$ and $\mathcal {C}_\nu$. By (4.9), (4.10) and (4.21), if $({\bar{y}}, {\bar{z}}) \in \mathcal {U}_\nu ^3 \setminus \mathcal {C}_\nu$ we have $\nu ^2 - O(\left| {\bar{y}}\right| ^3) \le \rho ^2 \le \nu ^2$. Since $\left| {\bar{y}}\right| \le \rho \le \nu$, we find a purely geometric constant ${\bar{c}}>0$ such that $\rho ^2 \in ( \nu ^2- {\bar{c}} \nu ^{3}, \nu ^2 )$. Similarly, if $({\bar{y}}, {\bar{z}}) \in \mathcal {C}_\nu \setminus \mathcal {U}_\nu ^3$, we have that $\rho ^2 \in ( \nu ^2, \nu ^2 + {\bar{c}} \nu ^{3})$. To summarize, we have that

$$\begin{aligned} \mathcal {U}^3_{\nu } \Delta \mathcal {C}_{\nu } \subset \{ \nu - {\bar{c}} \nu ^2 \le \rho \le \nu + {\bar{c}} \nu ^2 \}. \end{aligned}$$

Since $w_\varepsilon$ is Lipschitz, *f* satisfies (4.15) and $s \in (0,1)$, we have

$$\begin{aligned} \lim _{\nu \rightarrow 0} \left| \int _{\mathcal {U}^3_{\nu }} \frac{g_\varepsilon ({\bar{y}}, {\bar{z}}, z_0) }{\rho ^{d+2s}} \, d {\bar{y}}\, d{\bar{z}} - \int _{\mathcal {C}_\nu } \frac{g_\varepsilon ({\bar{y}}, {\bar{z}}, z_0) }{\rho ^{d+2s}} \, d {\bar{y}}\, d{\bar{z}} \right|&\lesssim \lim _{\nu \rightarrow 0} \int _{\nu - {\bar{c}} \nu ^2}^{\nu + {\bar{c}} \nu ^2} \frac{\rho + \rho ^2 }{\rho ^{d+2s}} \rho ^{d-1} \, d \rho = 0. \end{aligned}$$

Here the implicit constant depends on $\varepsilon$, but it is independent of $\nu$. Hence, (4.20) is proved.

Step 4: Collecting the estimates. To summarize, by (4.20) and letting $f,g_\varepsilon$ be as in (4.15), (4.19), we have that

4.22 $$\begin{aligned} \lim _{\nu \rightarrow 0} \Delta _\nu ^s u_\varepsilon (x_0)&= \lim _{\nu \rightarrow 0} \int _{\mathcal {C}_\nu } \frac{w_\varepsilon (z_0 + {\bar{z}}) - w_\varepsilon (z_0) }{\rho ^{d+2s}} (1- {\bar{z}} H_{\Sigma }(x_0')) \, d {\bar{z}} \, d {\bar{y}} \nonumber \\&\quad + \int _{\mathcal {C}_\nu } \frac{w_\varepsilon '(z_0 + {\bar{z}}) f(z_0, {\bar{y}})}{\rho ^{d+2s}} (1- {\bar{z}} H_{\Sigma }(x_0')) \, d{\bar{z}}\, d {\bar{y}}\nonumber \\&\quad + \int _{\mathcal {C}_\nu } \frac{w_\varepsilon (z_0 + {\bar{z}} + f(z_0, {\bar{y}}) ) - w_\varepsilon (z_0+ {\bar{z}}) - w_\varepsilon '(z_0+{\bar{z}}) f(z_0, {\bar{y}}) }{\rho ^{d+2s}} (1- {\bar{z}} H_{\Sigma }(x_0'))\, d{\bar{z}}\, d {\bar{y}}\nonumber \\&\quad + O \left( \int _{\mathcal {C}_\nu } \frac{\left| w_\varepsilon (z_0+ {\bar{z}} + f(z_0, {\bar{y}}) )- w_\varepsilon (z_0) \right| }{\rho ^{d+2s}} ( \left| z_0\right| \left| {\bar{y}}\right| + \rho ^2 ) \, d{\bar{z}} \, d {\bar{y}} \right) .\nonumber \\&= \lim _{\nu \rightarrow 0} I_1^\nu + I_2^\nu + I_3^\nu + O(I_4^\nu ). \end{aligned}$$

To conclude the proof, we estimate separately the four terms. For the reader’s convenience, we postpone these computations to Sect. 4.1. We summarize the contribution of each term:

(i) $I_1^\nu$ gives the fractional Laplacian of *w* and a contribution of the principal curvatures;
(ii) $I_2^\nu$ gives the remaining part of the term involving the principal curvatures;
(iii) $I_3^\nu$ is the nonlinear error;
(iv) $I_4^\nu$ is the error when expanding the ambient distance and the Jacobian.

To conclude, by (4.4), (4.22), Lemmas 4.3–4.6, we infer that

$$\begin{aligned} \lim _{\nu \rightarrow 0} \gamma _{d,s} \Delta ^s_\nu u_\varepsilon (x_0) = -(-\Delta )^s w_ \varepsilon (x_0) - \frac{\gamma _{1,s}}{2} \frac{H_{\Sigma } (x_0')}{2s-1} \int _{-{\delta }/{\Lambda }}^{{\delta }/{\Lambda }} \frac{w_\varepsilon '(z_0+{\bar{z}})}{\left| {\bar{z}}\right| ^{2s-1}} \, d{\bar{z}} + \mathcal {R}_{\Lambda , \varepsilon }(x_0), \end{aligned}$$

where $\mathcal {R}_{\Lambda ,\varepsilon }: \Sigma _{{\delta }/{10 \Lambda }} \rightarrow \mathbb {R}$ is a bounded function satisfying (4.3). Then, the proof is concluded. $\square$

### Estimates of the four integrals

We estimate the terms $I_1^\nu , I_2^\nu , I_3^\nu , I_4^\nu$ in (4.22). Throughout this section, we implicitly assume $\varepsilon \in (0,1)$ and $\Lambda \ge \Lambda _0$, where $\Lambda _0$ is given by Lemma 2.17. We neglect constants $C(d,s,W,\delta , \Sigma )>0$, whereas it is crucial to keep the dependence of $\varepsilon , \Lambda$ explicit We recall that *w* is the optimal profile and $w_\varepsilon (z) = w \left( {z}/{\varepsilon }\right)$. We need a preliminary lemma.

#### Lemma 4.2

Fix $s \in ({1}/{2},1)$. For any $z_0 \in \mathbb {R}, \ell , \varepsilon >0$ it holds

4.23 $$\begin{aligned}  &   \int _{-\ell }^\ell \frac{w_\varepsilon (z_0+z)-w_\varepsilon (z_0)}{\left| z\right| ^{2s+1}} z \, dz \nonumber \\  &   \quad = \frac{\ell ^{1-2s}}{1-2s} (w_\varepsilon (\ell +z_0) - w_\varepsilon (z_0-\ell )) + \frac{1}{2s-1} \int _{-\ell }^\ell \frac{w_\varepsilon '(z_0+z)}{\left| z\right| ^{2s-1}} \, dz. \end{aligned}$$

#### Proof

We have that

$$\begin{aligned} \int _{-\ell }^\ell \frac{w_\varepsilon (z_0+z) - w_\varepsilon (z_0)}{\left| z\right| ^{2s+1}} z \, dz = \left( \int _0^\ell + \int _{-\ell }^0 \right) \frac{w_\varepsilon (z_0+z) - w_\varepsilon (z_0)}{\left| z\right| ^{2s+1}} z \, dz = I+II. \end{aligned}$$

Then, carefully integrating by parts *I*, we get that

$$\begin{aligned} I&= \lim _{\nu \rightarrow 0} \int _{\nu }^\ell \frac{w_\varepsilon (z_0+z)-w_\varepsilon (z_0)}{z^{2s}} \, dz \\  &= \lim _{\nu \rightarrow 0} \left[ \frac{z^{1-2s}}{1-2s} (w_\varepsilon (z_0+z)-w_\varepsilon (z_0)) \right] _{z=\nu }^{z=\ell } + \frac{1}{2s-1} \int _{\nu }^\ell \frac{w_\varepsilon '(z_0+z)}{z^{2s-1}} \, dz \\  &= \lim _{\nu \rightarrow 0} \frac{\ell ^{1-2s}}{1-2s} (w_\varepsilon (\ell +z_0)-w_\varepsilon (z_0)) - \frac{\nu ^{1-2s}}{1-2s} (w_\varepsilon (\nu +z_0) - w_\varepsilon (z_0)) \\&+ \frac{1}{2s-1} \int _{\nu }^\ell \frac{w_\varepsilon '(z_0+z)}{z^{2s-1}} \, dz. \end{aligned}$$

Since $w_\varepsilon$ is Lipschitz continuous and $s \in (0,1)$, we have that

$$\begin{aligned} \lim _{\nu \rightarrow 0} \left| \frac{\nu ^{1-2s }}{1-2s} (w_\varepsilon (\nu +z_0) - w_\varepsilon (z_0))\right| \lesssim \lim _{\nu \rightarrow 0} \nu ^{2-2s} = 0. \end{aligned}$$

Recalling that $w_\varepsilon$ is increasing, by monotone convergence, we have that

$$\begin{aligned}&I -\frac{\ell ^{1-2s}}{1-2s} (w_\varepsilon (\ell +z_0)-w_\varepsilon (z_0)) \\&= \lim _{\nu \rightarrow 0} \frac{1}{2s-1} \int _{\nu }^\ell \frac{w_\varepsilon '(z_0+z)}{z^{2s-1}}\, dz \\&\quad = \frac{1}{2s-1} \int _0^{\ell } \frac{w_\varepsilon '(z_0+z)}{z^{2s-1}} \, dz. \end{aligned}$$

Similarly, it is easy to check that

$$\begin{aligned} II = \frac{\ell ^{1-2s}}{1-2s} (w_\varepsilon (z_0) - w_\varepsilon (z_0-\ell )) + \frac{1}{2s-1} \int _{-\ell }^0 \frac{w_\varepsilon '(z_0+z)}{\left| z\right| ^{2s-1}} \, dz. \end{aligned}$$

$$\square $$

#### Lemma 4.3

Let $I_1^\nu$ be given by (4.22). It holds

$$\begin{aligned} \sup _{x_0 \in \Sigma _{{\delta }/{10 \Lambda }}} \left| \lim _{\nu \rightarrow 0} I_1^\nu + \frac{1}{\gamma _{d,s}}(-\Delta )^s w_\varepsilon (z_0) + \frac{\gamma _{1,s}}{\gamma _{d,s}} \cdot \frac{H_{\Sigma }(x_0') }{(2s-1)} \int _{-{\delta }/{\Lambda }}^{{\delta }/{\Lambda }} \frac{w_\varepsilon '(z_0+{\bar{z}})}{\left| {\bar{z}}\right| ^{2s-1}} \, d{\bar{z}} \right| \lesssim \Lambda ^{2s}. \end{aligned}$$

#### Proof

For simplicity, we denote by $\ell = {\delta }/{\Lambda }$. Using the second order difference to get rid of the principal value, we split

$$\begin{aligned} \lim _{\nu \rightarrow 0} I_1^\nu&= \int _{B_\ell ^{d-1}} \int _{-\ell }^\ell \frac{w_\varepsilon (z_0 + {\bar{z}}) + w_\varepsilon (z_0-{\bar{z}}) -2 w_\varepsilon (z_0) }{2 \rho ^{d+2s}} \, d {\bar{z}} \, d {\bar{y}} \\  &\qquad - H_{\Sigma }(x_0') \int _{B_\ell ^{d-1}} \int _{-\ell }^\ell \frac{w_\varepsilon (z_0 + {\bar{z}}) - w_\varepsilon (z_0)}{\rho ^{d+2s}} {\bar{z}} \, d {\bar{z}} \, d {\bar{y}} \\  &= I_{1,1} + I_{1,2}. \end{aligned}$$

Estimate of $I_{1,1}$. By Lemma 6.4 and recalling that $\left| w_\varepsilon \right| \le 1$, we have

$$\begin{aligned} I_{1,1}&= \frac{\gamma _{1,s}}{\gamma _{d,s}} \int _{-\ell }^{\ell } \frac{w_\varepsilon (z_0 +{\bar{z}}) + w_\varepsilon (z_0 - {\bar{z}}) - 2 w_\varepsilon (z_0)}{2 \left| {\bar{z}}\right| ^{1+2s}} \, d{\bar{z}} + O\left( \ell ^{-2s}\right) \\&= - \frac{1}{\gamma _{d,s}} (-\Delta )^s w_\varepsilon (z_0) + O\left( \ell ^{-2s}\right) . \end{aligned}$$

Estimate of $I_{1,2}$. Similarly, we have

$$\begin{aligned} I_{1,2}&= -H_{\Sigma }(x_0') \int _{-\ell }^{\ell } \left( \frac{\gamma _{1,s}}{\gamma _{d,s}} \frac{w_\varepsilon (z_0 + {\bar{z}})- w_\varepsilon (z_0)}{\left| {\bar{z}}\right| ^{1+2s}} + O\left( \ell ^{-1-2s} \right) \right) {\bar{z}} \, d {\bar{z}} \\  &= - H_{\Sigma } (x_0') \frac{\gamma _{1,s}}{\gamma _{d,s}} \int _{-\ell }^{\ell } \frac{w_\varepsilon (z_0 + {\bar{z}})- w_\varepsilon (z_0)}{\left| {\bar{z}}\right| ^{1+2s}} {\bar{z}} \, d {\bar{z}} + O\left( \ell ^{1-2s} \right) . \end{aligned}$$

Then, using (4.23) and recalling that $\left| w_\varepsilon \right| \le 1$, we conclude

$$\begin{aligned} \left| I_{1,2} + H_{\Sigma }(x_0') \frac{\gamma _{1,s}}{(2s-1) \gamma _{d,s}} \int _{-\ell }^\ell \frac{w_\varepsilon '(z_0+{\bar{z}})}{\left| {\bar{z}}\right| ^{2s-1}} \, d{\bar{z}}\right| \lesssim \ell ^{1-2s}. \end{aligned}$$

$$\square $$

#### Lemma 4.4

Let $I_2^\nu$ be defined by (4.22). It holds

$$\begin{aligned} \sup _{x_0 \in \Sigma _{{\delta }/{10 \Lambda }}} \left| \lim _{\nu \rightarrow 0} I_2^\nu - \frac{H_{\Sigma }(x_0') }{2 (2s-1) } \cdot \frac{\gamma _{1,s}}{\gamma _{d,s}} \int _{-{\delta }/{\Lambda }}^{{\delta }/{\Lambda }} \frac{w'_\varepsilon (z_0+{\bar{z}})}{\left| {\bar{z}}\right| ^{2s-1}} \, d {\bar{z}} \right| \lesssim \Lambda ^{2s-1}. \end{aligned}$$

#### Proof

For simplicity, we denote by $\ell = {\delta }/{\Lambda }$. Then, we split

$$\begin{aligned} \lim _{\nu \rightarrow 0} I_2^\nu&= \int _{B_\ell ^{d-1}} \int _{-\ell }^\ell \frac{w_\varepsilon '(z_0+ {\bar{z}}) \sum _{i=1}^{d-1} k_i {\bar{y}}_i^2 }{2 \rho ^{d+2s}} (1- {\bar{z}} H_{\Sigma }(x_0')) \, d {\bar{z}}\, d {\bar{y}} \\  &\quad + O \left( \left| z_0\right| \int _{B_\ell ^{d-1}} \int _{-\ell }^\ell \frac{w'_\varepsilon (z_0 + {\bar{z}})}{\rho ^{d+2s}} \left| {\bar{y}}\right| ^2 \, d {\bar{z}} \, d {\bar{y}} \right) \\&+ O \left( \int _{B_\ell ^{d-1}} \int _{-\ell }^\ell \frac{w'_\varepsilon (z_0 + {\bar{z}})}{\rho ^{d+2s}} \left| {\bar{y}}\right| ^3 \, d {\bar{z}} \, d {\bar{y}} \right) \\  &= I_{2,1} + O\left( I_{2,2}\right) + O\left( I_{2,3}\right) \end{aligned}$$

and we estimate separately each term.

Estimate of $I_{2,1}$. By Lemma 6.4 and the fundamental theorem of calculus, we have that

$$\begin{aligned} I_{2,1}&= \frac{H_{\Sigma }(x_0')}{2(2s-1)} \int _{-\ell }^\ell w_\varepsilon '(z_0 + {\bar{z}})(1- {\bar{z}} H_{\Sigma }(x_0')) \left( \frac{\gamma _{1,s}}{\gamma _{d,s}} \cdot \frac{1}{\left| {\bar{z}}\right| ^{2s-1}} + O\left( \ell ^{1-2s}\right) \right) \, d{\bar{z}} \\  &= \frac{H_{\Sigma }(x_0') }{2 (2s-1) }\cdot \frac{\gamma _{1,s}}{\gamma _{d,s}} \int _{-\ell }^\ell \frac{w'_\varepsilon (z_0+{\bar{z}})}{\left| {\bar{z}}\right| ^{2s-1}} \, d {\bar{z}} + O\left( \ell ^{1-2s}\right) . \end{aligned}$$

Estimate of $I_{2,2}$. By Lemma 6.4 we compute

$$\begin{aligned} I_{2,2}&= \left| z_0\right| \int _{-\ell }^{\ell } w_\varepsilon '(z_0 + {\bar{z}}) \int _{B_\ell ^{d-1}} \frac{\left| {\bar{y}}\right| ^2}{ (\left| {\bar{z}}\right| ^2 + \left| {\bar{y}}\right| ^2)^{\frac{d+2s}{2}}}\, d {\bar{y}} \, d {\bar{z}} \\  &\lesssim \left| z_0\right| \int _{-\ell }^{\ell } \frac{ w_\varepsilon '(z_0 + {\bar{z}}) }{\left| {\bar{z}}\right| ^{2s-1}} \, d{\bar{z}} + \left| z_0\right| \ell ^{1-2s} \int _{-\ell }^{\ell } w_\varepsilon '(z_0 + {\bar{z}})\, d {\bar{z}} = A + B. \end{aligned}$$

By the fundamental theorem of calculus, we compute

$$\begin{aligned} B \lesssim \left| z_0\right| \ell ^{1-2s} \int _{\mathbb {R}} w_\varepsilon '(z_0 + {\bar{z}})\, d {\bar{z}} \lesssim \ell ^{2-2s}. \end{aligned}$$

To estimate *A*, we split

$$\begin{aligned} A = \left| z_0\right| \left( \int _{\left| {\bar{z}}\right| \le {\left| z_0\right| }/{2}} + \int _{{\left| z_0\right| }/{2} \le \left| {\bar{z}}\right| \le \ell } \right) \frac{w_\varepsilon '(z_0+ {\bar{z}})}{\left| {\bar{z}}\right| ^{2s-1}}\, d{\bar{z}} = A_1 + A_2. \end{aligned}$$

Then, by (2.4) we have

$$\begin{aligned} A_1&\lesssim \left| z_0\right| \sup _{\left| \zeta \right| \ge {\left| z_0\right| }/{2} } w_\varepsilon '(\zeta ) \int _{\left| {\bar{z}}\right| \le {\left| z_0\right| }/{2}} \frac{1}{\left| {\bar{z}}\right| ^{2s-1}} \, d {\bar{z}} \lesssim \frac{\left| z_0\right| }{\varepsilon } \frac{1}{1+ \left| z_0\right| ^{1+2s} \varepsilon ^{-1-2s}} \left| z_0\right| ^{2-2s} \lesssim \ell ^{2-2s}, \end{aligned}$$

since the function $t \mapsto \frac{t}{1+ \left| t\right| ^{1+2s}}$ is bounded in $\mathbb {R}$. To estimate $A_2$, we have

$$\begin{aligned} A_2 \lesssim \left| z_0\right| ^{2-2s} \int _{-\ell }^{\ell } w_\varepsilon '(z_0 + {\bar{z}})\, d {\bar{z}} \lesssim \ell ^{2-2s}. \end{aligned}$$

Estimate of $I_{2,3}$. Since $d+2s-3 < d-1$, we estimate

$$\begin{aligned} \left| I_{2,3}\right|&\lesssim \int _{-\ell }^\ell w_\varepsilon '(z_0 + {\bar{z}}) \int _{B_\ell ^{d-1}} \frac{1}{\left| {\bar{y}}\right| ^{d+2s-3}} \, d {\bar{y}} \lesssim \ell ^{2-2s} \int _{-\ell }^{\ell } w_\varepsilon '(z_0 + {\bar{z}}) \, d {\bar{z}} \lesssim \ell ^{2-2s}. \end{aligned}$$

$$\square $$

#### Lemma 4.5

Let $I_3^\nu$ be defined by (4.22). It holds that

$$\begin{aligned} \sup _{x_0 \in \Sigma _{{\delta }/{10 \Lambda }} } \limsup _{\nu \rightarrow 0} \left| I_3^\nu \right| \lesssim \varepsilon ^{2s} \Lambda ^{4s-2} + \Lambda ^{2s-2}. \end{aligned}$$

#### Proof

For simplicity, we denote by $\ell = {\delta }/{\Lambda }$. By Taylor’s expansion, we have

$$\begin{aligned} \lim _{\nu \rightarrow 0} I_3^\nu = \int _0^1 (1-t) \int _{B_{\ell }^{d-1}} \int _{-\ell }^{\ell } \frac{w_\varepsilon ''(z_0 + {\bar{z}} + t f(z_0, {\bar{y}}) ) f(z_0, {\bar{y}})^2 }{\rho ^{d+2s}} (1- {\bar{z}} H_{\Sigma }(x_0'))\, d{\bar{z}}\, d {\bar{y}} \end{aligned}$$

Hence, integrating by parts twice, we get that

$$\begin{aligned} \lim _{\nu \rightarrow 0} I_3^\nu&= \int _0^1 \int _{B_{\ell }^{d-1}} \bigg [ \left[ w'_\varepsilon (z_0 + {\bar{z}} + t f({\bar{y}}, z_0)) \frac{1- {\bar{z}} H_{\Sigma }(x_0')}{\rho ^{d+2s}} \right] _{{\bar{z}} = -\ell }^{{\bar{z}}= \ell } \\  &\qquad - \left[ w_{\varepsilon }(z_0+ {\bar{z}} + t f({\bar{y}}, z_0) ) \frac{d}{d {\bar{z}}} \left( \frac{1- {\bar{z}} H_{\Sigma }(x_0')}{\rho ^{d+2s}} \right) \right] _{{\bar{z}}=-\ell }^{{\bar{z}} = \ell } \\  &\qquad + \int _{-\ell }^{\ell } w_\varepsilon (z_0 + {\bar{z}} + t f({\bar{y}}, z_0)) \frac{d^2}{d {\bar{z}}^2} \left( \frac{1- {\bar{z}} H_{\Sigma }(x_0')}{\rho ^{d+2s}} \right) \, d {\bar{z}} \bigg ] f({\bar{y}}, z_0)^2 \, d {\bar{y}} \, dt \\  &= I_{3,1} + I_{3,2} + I_{3,3}. \end{aligned}$$

Estimate of $I_{3,1}$. To estimate $I_{3,1}$, by (2.4), we have

$$\begin{aligned} w'_\varepsilon \left( z_0 + \ell + t f({\bar{y}}, z_0)\right)&\lesssim \frac{ \varepsilon ^{-1}}{1+ \varepsilon ^{-1-2s} \left| z_0 + \ell + t f({\bar{y}}, z_0)\right| ^{1+2s} }\\&\lesssim \frac{ \varepsilon ^{2s} }{\left| z_0 + \ell + t f({\bar{y}}, z_0)\right| ^{1+2s}}. \end{aligned}$$

Moreover, by (4.15), it results that $\left| f({\bar{y}}, z_0)\right| \le C \ell ^2$ for any ${\bar{y}} \in B_{\ell }^{d-1}$. Here $C>0$ is a purely geometric constant. Recall that $\left| z_0\right| \le {\ell }/{10}$. Hence, we have that

$$\begin{aligned} \left| z_0 + \ell + t f({\bar{y}}, z_0)\right| \ge \ell - \frac{\ell }{10} - C \ell ^2 \ge \frac{\ell }{2}, \end{aligned}$$

provided that we take $\Lambda \ge \Lambda _0$, where $\Lambda _0$ in Lemma 2.17 is chosen large enough, depending on $\Sigma$. Thus, since $f({\bar{y}}, z_0)^2 = O (\left| {\bar{y}}\right| ^4)$ (see (4.15)), we estimate

$$\begin{aligned} \int _0^1 \int _{B_{\ell }^{d-1}}&\left[ w'_\varepsilon (z_0 + \ell + t f({\bar{y}}, z_0)) \frac{1- \ell H_{\Sigma }(x_0')}{( \ell ^2 + \left| {\bar{y}}\right| ^2 )^{\frac{d+2s}{2}}} \right] f({\bar{y}}, z_0)^2 \, d {\bar{y}} \, dt \lesssim \varepsilon ^{2s} \ell ^{2-4s}. \end{aligned}$$

Similarly, we estimate the evaluation in $-\ell$, thus proving that $\left| I_{3,1}\right| \lesssim \varepsilon ^{2s} \ell ^{2-4s}$.

Estimate of $I_{3,2}$. By direct computation, for any $({\bar{y}}, {\bar{z}}) \in B_{\ell }^{d-1} \times \left( -\ell ,\ell \right)$ we have

4.24 $$\begin{aligned}  &   \frac{d}{d {\bar{z}}} \left( \frac{1- {\bar{z}} H_{\Sigma }(x_0')}{\rho ^{d+2s}} \right) = -\frac{H_{\Sigma } (x_0') }{\rho ^{d+2s}} - (d+2s) \frac{{\bar{z}} - {\bar{z}}^2 H_{\Sigma }(x_0')}{\rho ^{d+2s+2}}, \end{aligned}$$

4.25 $$\begin{aligned}  &   \left| \frac{d^2}{d {\bar{z}}^2} \left( \frac{1- {\bar{z}} H_{\Sigma }(x_0')}{\rho ^{d+2s}} \right) \right| \lesssim \frac{1}{\rho ^{d+2s+2}}. \end{aligned}$$

Since $\left| w_\varepsilon \right| \le 1$, by (4.24) and $f({\bar{y}}, z_0)^2 = O (\left| {\bar{y}}\right| ^4)$ (see (4.15)), we estimate

$$\begin{aligned} \left| I_{3,2}\right| \lesssim \int _{B_\ell ^{d-1}} \frac{\left| {\bar{y}}\right| ^4}{ (\ell ^2 + \left| {\bar{y}}\right| ^2)^{\frac{d+2s}{2}} } \, d {\bar{y}} \lesssim \ell ^{3-2s} \end{aligned}$$

Estimate of $I_{3,3}$. Using (4.25), Lemma 6.4 and recalling that $f({\bar{y}}, z_0)^2 = O (\left| {\bar{y}}\right| ^4)$ (see (4.15)), $s \in \left( {1}/{2},1\right)$, it is readily checked that

$$\begin{aligned} \left| I_{3,3}\right|&\lesssim \int _{B_{\ell }^{d-1}} \int _{-\ell }^{\ell }\frac{\left| {\bar{y}}\right| ^4}{\rho ^{d+2s+2}} \, d{\bar{z}} \, d{\bar{y}} \lesssim \int _{-\ell }^{\ell } \left( \left| {\bar{z}}\right| ^{1-2s} + O\left( \ell ^{1-2s} \right) \right) \, d{\bar{z}} \lesssim \ell ^{2-2s}. \end{aligned}$$

$$\square $$

#### Lemma 4.6

Let $I_4^\nu$ be defined by (4.22). It holds that

$$\begin{aligned} \sup _{x_0 \in \Sigma _{{\delta }/{10 \Lambda }}} \limsup _{\nu \rightarrow 0} \left| I_4^\nu \right| \lesssim \Lambda ^{2s-2}. \end{aligned}$$

#### Proof

For simplicity, we denote by $\ell = {\delta }/{\Lambda }$. Then, we split

$$\begin{aligned} I_4&\le \left| z_0\right| \int _{B_\ell ^{d-1}} \int _{-\ell }^\ell \frac{\left| w_\varepsilon (z_0 + {\bar{z}} + f({\bar{y}}, z_0)) - w_\varepsilon (z_0+ {\bar{z}}) \right| }{\rho ^{d+2s-1}} \, d {\bar{z}} \, d {\bar{y}} \\  &\qquad + \left| z_0\right| \int _{B_\ell ^{d-1}} \int _{-\ell }^\ell \frac{\left| w_\varepsilon (z_0 + {\bar{z}}) - w_\varepsilon (z_0) \right| }{\rho ^{d+2s-1}} \, d {\bar{z}} \, d {\bar{y}} \\  &\qquad + \int _{B_\ell ^{d-1}} \int _{-\ell }^\ell \frac{\left| w_\varepsilon (z_0 + {\bar{z}} + f({\bar{y}}, z_0)) - w_\varepsilon (z_0) \right| }{\rho ^{d+2s-2}} \, d {\bar{z}} \, d {\bar{y}} \\  &= I_{4,1}+ I_{4,2}+ I_{4,3}. \end{aligned}$$

Estimate of $I_{4,1}$. Since $f({\bar{y}}, z_0) = O(\left| {\bar{y}}\right| ^2)$ (see (4.15)) and $\left| z_0\right| \le {\ell }/{10}$, by the fundamental theorem of calculus and integrating by parts, we have that

$$\begin{aligned}&I_{4,1} \le \ell \int _0^1 \int _{B_\ell ^{d-1}} \int _{-\ell }^\ell \frac{w'_\varepsilon (z_0 + {\bar{z}} + t f({\bar{y}}, z_0))}{\rho ^{d+2s-3}} \, d {\bar{z}} \, d {\bar{y}} \, dt\\&\quad = \ell \int _0^1 \int _{B_\ell ^{d-1}} \left( \left[ \frac{w_\varepsilon (z_0+ {\bar{z}} + t f({\bar{y}}, z_0))}{\rho ^{d+2s-3}} \right] _{{\bar{z}}= -\ell }^{{\bar{z}}= \ell } + (d+2s-3) \int _{-\ell }^\ell \frac{w_\varepsilon (z_0+ {\bar{z}} + t f({\bar{y}}, z_0)) {\bar{z}}}{\rho ^{d+2s-1}} \, d {\bar{z}} \right) \, d {\bar{y}} \, dt\\&\quad \lesssim \ell ^{3-2s} + \int _{B_\ell ^{d-1}} \int _{-\ell }^\ell \frac{1}{\rho ^{d+2s-2}} \, d {\bar{z}} \, d {\bar{y}} \lesssim \ell ^{2-2s}. \end{aligned}$$

Estimate of $I_{4,2}$. The estimate of $I_{4,2}$ is similar to that of $I_{2,2}$ in Lemma 4.4. By the fundamental theorem of calculus and recalling that $d+2s-1 > d-1$, we compute

$$\begin{aligned} \left| I_{4,2}\right|&\le \left| z_0\right| \int _0^1 \int _{-\ell }^\ell \left| {\bar{z}}\right| w_\varepsilon '(z_0 + t {\bar{z}}) \int _{B_\ell ^{d-1}} \frac{1}{(\left| {\bar{z}}\right| ^2 +\left| {\bar{y}}\right| ^2)^{\frac{d+2s-1}{2}}} \, d {\bar{y}} \, d {\bar{z}} \, dt \\  &= \left| z_0\right| \int _0^1 \int _{-\ell }^\ell \left| {\bar{z}}\right| ^{1-2s} w_\varepsilon '(z_0 + t {\bar{z}}) \int _{B_{{\ell }/{\left| {\bar{z}}\right| }}^{d-1}} \frac{1}{(1 +\left| {\bar{y}}\right| ^2)^{\frac{d+2s-1}{2}}} \, d {\bar{y}} \, d {\bar{z}} \, dt \\  &\le \left| z_0\right| \int _0^1 \frac{1}{t^{2-2s}} \int _{-\ell t}^{\ell t} \frac{w_\varepsilon '({\bar{z}} + z_0) }{\left| {\bar{z}}\right| ^{2s-1}}\, d {\bar{z}} \, dt. \end{aligned}$$

Since $$2-2s <1 $$, we have

$$\begin{aligned} \left| I_{4,2}\right| \lesssim \left| z_0\right| \int _{-\ell }^{\ell } \frac{w_\varepsilon '({\bar{z}} + z_0) }{\left| {\bar{z}}\right| ^{2s-1}}\, d {\bar{z}} \lesssim \ell ^{2-2s} \end{aligned}$$

as for the term *A* in the estimate of $I_{2,2}$ in Lemma 4.4.

Estimate of $I_{4,3}$ . Since $\left| w_\varepsilon \right| \le 1$ and $d+2\,s-2 < d$, we have that

$$\begin{aligned} I_{4,3}&\lesssim \int _{B_\ell ^{d-1}} \int _{-\ell }^\ell \frac{1}{\rho ^{d+2s-2}} \, d {\bar{z}} \, d {\bar{y}} \lesssim \ell ^{2-2s}. \end{aligned}$$

$$\square $$

## On the finiteness of a constant

In this section, we show that the constant $\kappa _\star$ in (1.9) is finite. Motivated by Theorem 4.1, we introduce the following notation.

### Definition 5.1

Fix $s\in ({1}/{2},1)$ and let *w* be the optimal profile of Theorem 2.2. For $\ell , \varepsilon >0$, set $w_\varepsilon (z) = w \left( {z}/{\varepsilon }\right)$ and define

5.1 $$\begin{aligned} \eta _{\varepsilon ,\ell }(z_0) = \int _{-\ell }^\ell \frac{w_\varepsilon '(z_0+z)}{\left| z\right| ^{2s-1}} \, dz. \end{aligned}$$

### Remark 5.2

Let $\eta _{\varepsilon ,\ell }$ be given by (5.1). Since *w* is strictly increasing and $w'$ is even and globally integrable, then $\eta _{\varepsilon , \ell }$ is nonnegative, even and globally bounded. Moreover, by the change of variable $z= {{\bar{z}}}/{\varepsilon }$ it holds

5.2 $$\begin{aligned} \eta _{\varepsilon ,\ell }(z_0) = \varepsilon ^{1-2s} \eta _{1,{\ell }/{\varepsilon }}\left( {z_0 }/{\varepsilon } \right) . \end{aligned}$$

Finally, we show the following result.

### Proposition 5.3

Fix $\ell , \ell '>0$. Let $\eta _{\varepsilon ,\ell }$ be defined by (5.1). For any $s\in ({3}/{4},1)$, it holds

5.3 $$\begin{aligned} \lim _{\varepsilon \rightarrow 0^+} \int _{-{\ell '}/{\varepsilon }}^{{\ell '}/{\varepsilon }} \eta _{1, {\ell }/{\varepsilon }}(z)^2 \, dz =: \mu _w \in (0,\infty ). \end{aligned}$$

The constant $\mu _w$ depends only on *w* and it is independent of $\ell , \ell '$. For $s = {3}/{4}$, it holds

5.4 $$\begin{aligned} \lim _{\varepsilon \rightarrow 0^+} \frac{1}{\left| \log (\varepsilon )\right| } \int _{-{\ell '}/{\varepsilon }}^{{\ell '}/{\varepsilon }} \eta _{1, {\ell }/{\varepsilon }}(z)^2 \, dz = 8. \end{aligned}$$

### Proof

Fix $\ell , \ell ' >0$. Given $\varepsilon >0$ we estimate the decay of the inner integral uniformly with respect to $\varepsilon$. From now on, unless otherwise specified, we neglect constants depending on $s,W, \ell , \ell '$. By the decay of $w'$ (see Theorem 2.2) we estimate

5.5 $$\begin{aligned} \sup _{\varepsilon >0} \sup _{\left| z\right| \le 1} \int _{-{\ell }/{\varepsilon }}^{{\ell }/{\varepsilon }} \frac{w'(z+t)}{\left| t\right| ^{2s-1}} \, dt \lesssim \int _{\left| t\right| \le 2} \frac{1}{\left| t\right| ^{2s-1}} \, dt + \sup _{\left| z\right| \le 1} \int _{ \left| t\right| \ge 2 } \frac{1}{1 + \left| t+z\right| ^{1+2s} } \, dt < +\infty . \nonumber \\ \end{aligned}$$

To estimate the inner integral for $\left| z\right| \ge 1$, we fix a constant $\nu >1$ and we define

$$\begin{aligned} I_{z, \nu }:= \int _{\left| t\right| \le {\left| z\right| }/{\nu }} \frac{w'(t+z)}{\left| t\right| ^{2s-1}}\, dt, \quad II_{z, \nu } =: \int _{{\left| z\right| }/{\nu } \le \left| t\right| \le \nu \left| z\right| } \frac{w'(t+z)}{\left| t\right| ^{2s-1}} \, dt, \quad III_{z,\nu }:= \int _{ \left| t\right| \ge \nu \left| z\right| } \frac{w'(t+z)}{\left| t\right| ^{2s-1}} \, dt. \end{aligned}$$

For the second term we have

$$\begin{aligned} \nu ^{1-2s} \left| z\right| ^{1-2s} \int _{{\left| z\right| }/{\nu } \le \left| t\right| \le \nu \left| z\right| } w'(t+z) \, dt \le II_{z,\nu } \le \nu ^{2s-1} \left| z\right| ^{1-2s} \int _{{\left| z\right| }/{\nu } \le \left| t\right| \le \nu \left| z\right| } w'(t+z) \, dt. \end{aligned}$$

Moreover, if $z\ge 1$ by direct computation and (2.5), we estimate

$$\begin{aligned}&\int _{{\left| z\right| }/{\nu } \le \left| t\right| \le \nu \left| z\right| } w'(t+z) \, dt \\&= w(z(1+\nu )) - w\left( z \left( 1+ \frac{1}{\nu }\right) \right) + w\left( z \left( 1- \frac{1}{\nu }\right) \right) - w(z(1-\nu )) \\  &= 2 + O(\left| z\right| ^{-2s}), \end{aligned}$$

where the reminder depends also on $\nu$. The same estimate can be proved for $z<-1$. Hence, for $\left| z\right| \ge 1$ we have that

5.6 $$\begin{aligned} 2 \nu ^{1-2s} \left| z\right| ^{1-2s} + O(\left| z\right| ^{1-4s}) \le II_{z,\nu } \le 2 \nu ^{2s-1} \left| z\right| ^{1-2s} + O(\left| z\right| ^{1-4s}). \end{aligned}$$

By Theorem 2.2, for $\left| z\right| \ge 1$ we have the following estimate for the first term

5.7 $$\begin{aligned}  &   I_{z,\nu } \lesssim \left| z\right| ^{-1-2s} \left( 1- \frac{1}{\nu }\right) ^{-1-2s} \int _{\left| t\right| \le {\left| z\right| }/{\nu }} \frac{1}{\left| t\right| ^{2s-1}}\, dt\nonumber \\  &   \quad \le O(\left| z\right| ^{1-4s}), \end{aligned}$$

where the reminder depends on $\nu$. Similarly, for the third term, for $\left| z\right| \ge 1$ we have

5.8 $$\begin{aligned}  &   III_{z, \nu } \le \nu ^{1-2s} \left| z\right| ^{1-2s} \int _{\left| t\right| \ge \nu \left| z\right| } w'(t+z) \, dt \le \nu ^{1-2s} \left| z\right| ^{1-2s} \int _{\left| t'\right| \ge \left| z\right| (\nu -1)} w'(t')\, dt' \nonumber \\  &   \quad \le O(\left| z\right| ^{1-4s}), \end{aligned}$$

where the reminder depends on $\nu$. Then, if $\left| z\right| \in \left( 1, {\ell }/{\varepsilon \nu }\right)$, we have that

$$\begin{aligned} II_{z, \nu } \le \int _{-{\ell }/{\varepsilon }}^{{\ell }/{\varepsilon }} \frac{w'(t+z)}{\left| t\right| ^{2s-1}} \, dt \le I_{z,\nu } + II_{z,\nu } +III_{z,\nu } \end{aligned}$$

and by (5.6)–(5.8) we estimate

5.9 $$\begin{aligned} 2\nu ^{1-2s} \left| z\right| ^{1-2s} + O(\left| z\right| ^{1-4s}) \le \int _{-{\ell }/{\varepsilon }}^{{\ell }/{\varepsilon }} \frac{w'(z+t)}{\left| t\right| ^{2s-1}} \, dt \le 2 \nu ^{2s-1} \left| z\right| ^{1-2s} + O(\left| z\right| ^{1-4s}).\nonumber \\ \end{aligned}$$

Here the reminders depend on $\nu$, but they are independent of $\varepsilon$. With the same technique, for $\left| z\right| \in \left( {\ell }/{\varepsilon \nu }, {\ell \nu }/{\varepsilon } \right)$ we have

5.10 $$\begin{aligned} \int _{-{\ell }/{\varepsilon }}^{{\ell }/{\varepsilon }} \frac{w'(z+t)}{\left| t\right| ^{2s-1}} \, dt \le I_{z,\nu } + II_{z,\nu } \le O(\left| z\right| ^{1-2s}). \nonumber \\ \end{aligned}$$

Similarly, for $\left| z\right| \ge {\ell \nu }/{\varepsilon }$ we have

5.11 $$\begin{aligned} \int _{-{\ell }/{\varepsilon }}^{{\ell }/{\varepsilon }} \frac{w'(z+t)}{\left| t\right| ^{2s-1}} \, dt \le I_{z,\nu } \le O(\left| z\right| ^{1-4s}). \end{aligned}$$

Here the reminders depend on $\nu$ but not on $\varepsilon$. In conclusion, if $s \in \left( {3}/{4}, 1\right)$ it is trivial to see that

$$\begin{aligned} \mu _w = \int _{\mathbb {R}} \left( \int _{\mathbb {R}} \frac{w'(t+z)}{\left| t\right| ^{2s-1}} \, dt \right) ^2 \, d z \in (0,+\infty ) \end{aligned}$$

and it is independent of $\ell , \ell '$. To show that $\mu _w$ is finite, using (5.5), (5.9)–(5.11) with $\nu =2$, we have

$$\begin{aligned} \sup _{\varepsilon> 0} \int _{-{\ell '}/{\varepsilon }}^{{\ell '}/{\varepsilon }} \left( \int _{-{\ell }/{\varepsilon }}^{{\ell }/{\varepsilon } } \frac{w'(t+z)}{\left| t\right| ^{2s-1}} \, dt \right) ^2 \, dz&\lesssim 1+ \int _{ \left| z\right| >1 } \left| z\right| ^{2-4s}\, dz < +\infty . \end{aligned}$$

To conclude, we consider the case $s = {3}/{4}$. Then, using (5.5), (5.9)–(5.11) with $\nu >1$ (to be chosen later), we have

5.12 $$\begin{aligned}&\limsup _{\varepsilon \rightarrow 0^+} \frac{1}{\left| \log (\varepsilon )\right| }\int _{-{\ell '}/{\varepsilon }}^{{\ell '}/{\varepsilon }} \left( \int _{-{\ell }/{\varepsilon }}^{{\ell }/{\varepsilon } } \frac{w'(t+z)}{\left| t\right| ^{\frac{1}{2}}} \, dt \right) ^2 \, dz \le \limsup _{\varepsilon \rightarrow 0^+} \frac{1}{\left| \log (\varepsilon )\right| }\int _{\mathbb {R}} \left( \int _{-{\ell }/{\varepsilon }}^{{\ell }/{\varepsilon } } \frac{w'(t+z)}{\left| t\right| ^{\frac{1}{2}}} \, dt \right) ^2 \, dz \ \nonumber \\&\quad \le \limsup _{\varepsilon \rightarrow 0^+} \frac{1}{\left| \log (\varepsilon )\right| } \left( \int _{\left| z\right| \le 1} + \int _{1 \le \left| z\right| \le {\ell }/{\varepsilon \nu }} + \int _{{\ell }/{\varepsilon \nu } \le \left| z\right| \le {\ell \nu }/{\varepsilon }} + \int _{ \left| z\right| \ge {\ell \nu }/{\varepsilon } } \right) \left( \int _{-{\ell }/{\varepsilon }}^{{\ell }/{\varepsilon } } \frac{w'(t+z)}{\left| t\right| ^{\frac{1}{2}}} \, dt \right) ^2 \, dz\nonumber \\&\quad \le \limsup _{\varepsilon \rightarrow 0^+} \frac{1}{\left| \log (\varepsilon )\right| } \bigg ( O(1) + \int _{1 \le \left| z\right| \le {\ell }/{\varepsilon \nu } } \left( \frac{4\nu }{\left| z\right| } + O(\left| z\right| ^{-4}) \right) \, dz + \int _{ {\ell }/{\varepsilon \nu } \le \left| z\right| \le {\ell \nu }/{ \varepsilon } } O(\left| z\right| ^{-1}) \, dz\nonumber \\&\qquad + \int _{ \left| z\right| \ge {\ell \nu }/{\varepsilon } } O(\left| z\right| ^{-4}) \, dz \bigg ) = 8 \nu . \end{aligned}$$

Using the lower bound in (5.9) and setting ${\bar{\ell }}:= \min \{\ell , \ell '\}$, we estimate

5.13 $$\begin{aligned} \liminf _{\varepsilon \rightarrow 0^+} \frac{1}{\left| \log (\varepsilon )\right| }&\int _{-{\ell '}/{\varepsilon }}^{{\ell '}/{\varepsilon }} \left( \int _{-{\ell }/{\varepsilon }}^{{\ell }/{\varepsilon } } \frac{w'(t+z)}{\left| t\right| ^{\frac{1}{2}}} \, dt \right) ^2 \, dz\nonumber \\  &\ge \liminf _{\varepsilon \rightarrow 0^+} \frac{1}{\left| \log (\varepsilon )\right| }\int _{ 1 \le \left| z\right| \le {{\bar{\ell }}}/{\varepsilon \nu } } \left( \int _{-{\ell }/{\varepsilon }}^{{\ell }/{\varepsilon } } \frac{w'(t+z)}{\left| t\right| ^{\frac{1}{2}}} \, dt \right) ^2 \, dz \nonumber \\  &\ge \liminf _{\varepsilon \rightarrow 0^+} \frac{1}{\left| \log (\varepsilon )\right| } \int _{1 \le \left| z\right| \le {{\bar{\ell }}}/{\varepsilon \nu } } \left( \frac{4 \nu ^{-1}}{\left| z\right| } + O(\left| z\right| ^{-4}) \right) \, dz \ge 8 \nu ^{-1}. \end{aligned}$$

Combining (5.12) and (5.13) and letting $\nu \rightarrow 1^+$, we prove (5.4). $\square$

## Proof of the main result

This section is entirely devoted to the proof of Theorem 1.1. Our analysis is based on the following heuristic argument which allows to guess the right scaling of the energy for different values of the parameter $s \in \left( {1}/{2},1\right)$. Given a smooth set *E*, let $u_\varepsilon (x) = w_\varepsilon (\beta _\Sigma (x))$, where $w_\varepsilon$ is the scaled one-dimensional optimal profile and $\beta _\Sigma$ is the modified signed distance function (see Definition 2.10). We split the energy in the contribution around the interface and the contribution far from the interface. By Theorem 4.1, we exploit the cancellations in the energy around the interface due to the structure of the recovery sequence and we use the decay property of the optimal profile to estimate separately the two terms in the integral away from the interface. More precisely, it is not difficult to see that

$$\begin{aligned}&\int _{\Omega } \left( \varepsilon ^{2s-1} (-\Delta )^s u_\varepsilon + \frac{W'(u_\varepsilon )}{\varepsilon } \right) ^2 \, dx\\&\simeq \, \varepsilon ^{4s-2} \mathcal {W}(\Sigma , \Omega ) \int _{-\delta }^\delta \left( \int _{-\delta }^\delta \frac{w_\varepsilon '(z+t)}{\left| t\right| ^{2s-1}} \, dt \right) ^2 \, dz\\&\quad + \varepsilon ^{4s-2} \left( \Vert {R_\varepsilon } \Vert _{L^\infty (\Sigma _\delta )}^2 + \Vert {(-\Delta )^s u_\varepsilon } \Vert ^2_{L^\infty (\Omega \setminus \Sigma _\delta )}\right) \\&\quad + \varepsilon ^{-2} \Vert {W'(u_\varepsilon )} \Vert _{L^\infty (\Omega \setminus \Sigma _\delta )}^2\\&\simeq \, \varepsilon \mathcal {W}(\Sigma , \Omega ) \int _{-{\delta }/{\varepsilon }}^{{\delta }/{\varepsilon }} \left( \int _{-{\delta }/{\varepsilon }}^{{\delta }/{\varepsilon }} \frac{w'(z+t)}{\left| t\right| ^{2s-1}} \, dt \right) ^2 \, dz + O(\varepsilon ^{4s-2}). \end{aligned}$$

Moreover, we have

$$\begin{aligned} \int _{-{\delta }/{\varepsilon }}^{{\delta }/{\varepsilon }} \left( \int _{-{\delta }/{\varepsilon }}^{{\delta }/{\varepsilon }} \frac{w'(z+t)}{\left| t\right| ^{2s-1}} \, dt \right) ^2 \, dz \simeq {\left\{ \begin{array}{ll} 1 & \text { if } s \in \left( {3}/{4},1 \right) , \\[0.5ex] \left| \log (\varepsilon )\right| &  \text { if } s = {3}/{4}, \\[0.5ex] \varepsilon ^{4s-3} &  \text { if } s \in ({1}/{2},{3}/{4}), \end{array}\right. } \end{aligned}$$

where we proved the first two estimates in Lemma 5.3, while the last one can be obtained with an simple modification of the argument. Therefore, we conclude that

$$\begin{aligned} \int _{\Omega } \left( \varepsilon ^{2s-1} (-\Delta )^s u_\varepsilon + \frac{W'(u_\varepsilon )}{\varepsilon } \right) ^2 \, dx \simeq {\left\{ \begin{array}{ll} \varepsilon \mathcal {W}(\Sigma , \Omega ) + O(\varepsilon ^{4s-2}) &  s \in \left( {3}/{4}, 1 \right) , \\[1ex] \varepsilon \left| \log \varepsilon \right| \mathcal {W}(\Sigma , \Omega ) + O(\varepsilon ^{4s-2}) &  s = {3}/{4}, \\[1ex] \varepsilon ^{4s-2} \mathcal {W}(\Sigma , \Omega ) + O(\varepsilon ^{4s-2}) &  s \in \left( {1}/{2}, {3}/{4} \right) . \end{array}\right. } \end{aligned}$$

This argument shows different behaviours according to the value of the parameter *s*. Indeed, if $s > {3}/{4}$ then $\varepsilon \mathcal {W}(\Sigma , \Omega )$ is the leading order term and, after dividing the energy by $\varepsilon$, we see the Willmore functional in the limit. The situation is similar for $s = {3}/{4}$, provided that we divide the energy by $\varepsilon \left| \log (\varepsilon )\right|$. Roughly speaking, if $s \in \left[ {3}/{4}, 1\right)$, it turns out that the energy around the interface is much larger than the energy away from the interface and, after introducing an appropriate scaling, the limit has a purely local behaviour. In the proof of Theorem 1.1, we make this argument rigorous. This heuristic breaks down when $s \in \left( {1}/{2}, {3}/{4}\right)$. Our analysis suggests that all terms have the same order of magnitude and, after dividing the energy by $\varepsilon ^{4s-2}$, a local/nonlocal behaviour might persist in the limit. To conclude, it seems that a finer analysis of both the error term in the expansion of the fractional Laplacian around the interface and the energy away from the interface would be needed to understand the limiting behaviour of our energy in the case $s \in \left( {1}/{2}, {3}/{4}\right)$. From now on, we focus on the case $s \in \left[ {3}/{4},1\right)$.

### Proof of Theorem 1.1

We divide the proof in some steps. We recall that $c_\star$ is the constant in (1.6) and we define

6.1 $$\begin{aligned} \kappa _\star := {\left\{ \begin{array}{ll} \displaystyle \frac{\gamma _{1,s}^2}{4(2s-1)^2} \int _{\mathbb {R}} \left( \int _{\mathbb {R}} \frac{w'(t+z)}{\left| t\right| ^{2s-1}} \, dt \right) ^2 \, dz &  s \in \left( {3}/{4}, 1\right) , \\[0.5ex] 8 \gamma _{1, {3}/{4}}^2 &  s = {3}/{4}. \end{array}\right. } \end{aligned}$$

Step 1. Let $s \in \left( {3}/{4}, 1 \right)$ and let $E \subset \mathbb {R}^d$ be a bounded open set with $\partial E \in C^3$. Define

6.2 $$\begin{aligned} u_\varepsilon (x) = w_\varepsilon (\beta _\Sigma (x)), \end{aligned}$$

where $w_\varepsilon (z) = w \left( {z}/{\varepsilon } \right)$, *w* is the one-dimensional optimal profile and $\beta _\Sigma$ is the modified distance function according to Definition 2.10. We claim that for any bounded open set $\Omega \subset \mathbb {R}^d$ we have

6.3 $$\begin{aligned} \limsup _{\varepsilon \rightarrow 0^+} \left( \mathcal {F}_{s, \varepsilon } + \mathcal {G}_{s,\varepsilon } \right) (u_\varepsilon , \Omega ) \le c_\star \mathcal {H}^{d-1}(\partial E \cap {\overline{\Omega }}) + \kappa _{\star } \int _{\partial E \cap {\overline{\Omega }}} H_{\partial E}(y)^2 \, d \mathcal {H}^{d-1}(y). \end{aligned}$$

The same conclusions holds for $s = {3}/{4}$ adding an extra $\left| \log (\varepsilon )\right| ^{-1}$ in front of $\mathcal {G}_{s,\varepsilon }$.

For $s \in \left[ {3}/{4}, 1\right)$, by a simple modification of the proof of [49, Proposition 4.7] due to the fact that $\beta _\Sigma$ is the proper distance function in a tubular neighbourhood of $\Sigma = \partial E$, it is readily checked that

$$\begin{aligned} \limsup _{\varepsilon \rightarrow 0^+} \mathcal {F}_{\varepsilon ,s}(u_\varepsilon , \Omega ) \le c_{\star } \mathcal {H}^{d-1}( \Sigma \cap {\overline{\Omega }}). \end{aligned}$$

We point out that no regularity on $\partial \Omega$ is needed. Therefore, if $s \in \left( {3}/{4}, 1 \right)$ it remains to check that

6.4 $$\begin{aligned} \limsup _{\varepsilon \rightarrow 0^+} \mathcal {G}_{s,\varepsilon }(u_\varepsilon , \Omega ) \le \kappa _\star \int _{\Sigma \cap {\overline{\Omega }}} H_{\Sigma }(y)^2 \, d \mathcal {H}^{d-1}(y). \end{aligned}$$

If $s = {3}/{4}$, (6.4) holds adding an extra $\left| \log (\varepsilon )\right| ^{-1}$ in front of $\mathcal {G}_{s,\varepsilon }$.

The case $s \in \left( {3}/{4}, 1 \right)$. Given $\delta >0$ as in Definition 2.10 and $\Lambda _0 \ge 1$ as in Theorem 4.1, for $\Lambda \ge \Lambda _0$ we split

$$\begin{aligned} \mathcal {G}_{s,\varepsilon }(u_\varepsilon , \Omega )&= \left( \frac{1}{\varepsilon } \int _{\Omega \cap \Sigma _{{\delta }/{10 \Lambda }} } + \frac{1}{\varepsilon } \int _{\Omega \setminus \Sigma _{{\delta }/{10 \Lambda }}} \right) \left( \varepsilon ^{2s-1} (-\Delta )^s u_\varepsilon (x) + \frac{W'(u_\varepsilon (x))}{\varepsilon } \right) ^2 dx\,\\&= I_{\varepsilon ,\Lambda } + II_{\varepsilon ,\Lambda }. \end{aligned}$$

We claim that

6.5 $$\begin{aligned} \lim _{\varepsilon \rightarrow 0^+} II_{\varepsilon ,\Lambda } = 0 \qquad \forall \Lambda \ge \Lambda _0. \end{aligned}$$

To deal with the energy away from the interface, we neglect constants independent of $\varepsilon$. Thus, for $\Lambda \ge \Lambda _0$, we have

$$\begin{aligned} II_{\varepsilon ,\Lambda }&\lesssim \varepsilon ^{4s-3} \int _{\Omega \setminus \Sigma _{{\delta }/{10 \Lambda }}} \left( (-\Delta )^s u_\varepsilon (x) \right) ^2 \, dx \\&\quad + \varepsilon ^{-3} \int _{\Omega \setminus \Sigma _{{\delta }/{10 \Lambda }}} \left( W'(u_\varepsilon (x)) \right) ^2 \, dx = II_{1, \varepsilon ,\Lambda } + II_{2, \varepsilon , \Lambda }. \end{aligned}$$

By Lemma 3.3, we conclude that $II_{1, \varepsilon , \Lambda } \rightarrow 0$ as $\varepsilon \rightarrow 0$ for $s>{3}/{4}$. Regarding $I_{2, \varepsilon , \Lambda }$, since $W'$ is Lipschitz and odd, using (2.5), the monotonicity of *w* and the fact that $\left| \beta _{\Sigma }(x)\right| > {\delta }/{10 \Lambda }$ in the domain of integration (see Definition 2.10), we have that

$$\begin{aligned} II_{2, \varepsilon ,\Lambda }&= \varepsilon ^{-3} \int _{ E \setminus \Sigma _{{\delta }/{10 \Lambda }}} (W'(u_\varepsilon (x)) - W'(1))^2\, dx + \varepsilon ^{-3} \int _{(\Omega \setminus E) \setminus \Sigma _{{\delta }/{10 \Lambda }} } (W'(u_\varepsilon (x)) - W'(-1) )^2 \, dx \\  &\lesssim \varepsilon ^{-3} \int _{E \setminus \Sigma _{{\delta }/{10 \Lambda }}} \left| u_\varepsilon (x) - 1\right| ^2\, dx + \varepsilon ^{-3} \int _{(\Omega \setminus E) \setminus \Sigma _{{\delta }/{10 \Lambda }} } \left| u_\varepsilon (x) + 1 \right| ^2 \, dx \\  &\lesssim \varepsilon ^{-3} \int _{E \setminus \Sigma _{{\delta }/{10 \Lambda }} } \left| 1- w\left( \frac{\delta }{10 \Lambda \varepsilon }\right) \right| ^2\, dx + \varepsilon ^{-3} \int _{(\Omega \setminus E) \setminus \Sigma _{{\delta }/{10 \Lambda }} } \left| 1+ w\left( -\frac{\delta }{10 \Lambda \varepsilon }\right) \right| ^2 \, dx \lesssim \varepsilon ^{4s-3}. \end{aligned}$$

Hence, we have that $II_{2,\varepsilon ,\Lambda } \rightarrow 0$ as $\varepsilon \rightarrow 0^+$, since $s >{3}/{4}$.

Letting be $\kappa _\star$ as in (6.1), it remains to check that

6.6 $$\begin{aligned} \inf _{\Lambda \ge \Lambda _0} \limsup _{\varepsilon \rightarrow 0^+} I_{\varepsilon ,\Lambda } \le \kappa _\star \int _{\Sigma \cap {\overline{\Omega }}} H_{\Sigma }(x) ^2 \, d \mathcal {H}^{d-1}(x). \end{aligned}$$

Then, (6.4) follows by (6.5) and (6.6). The proof of (6.6) is based on the expansion of the fractional Laplacian (4.2). Fix $\Lambda \ge \Lambda _0$. Since $\beta _\Sigma$ is the proper signed distance in $\Sigma _{{\delta }/{10 \Lambda }}$ (see Definition 2.10), we have

6.7 $$\begin{aligned}  &   I_{\varepsilon ,\Lambda } = \frac{1}{\varepsilon }\nonumber \\  &   \quad \int _{\Sigma _{{\delta }/{10 \Lambda }} \cap \Omega } \left( \frac{W'(u_\varepsilon (x))}{\varepsilon } + \varepsilon ^{2s-1} \left( (-\partial _{zz})^s w_\varepsilon (z) + \frac{\gamma _{1,s}}{4s-2} H_{\Sigma }(x') \eta _{\varepsilon ,{\delta }/{10 \Lambda }} (z) + \mathcal {R}_{\varepsilon , \Lambda }(x) \right) \right) ^2 \, dx, \nonumber \\ \end{aligned}$$

where we set $x'= \pi _{\Sigma }(x)$, $z = \textrm{dist}_{\Sigma }(x)$, $\eta _{\varepsilon , {\delta }/{10 \Lambda }}$ is given by (5.1) and $\mathcal {R}_{\varepsilon , \Lambda }$ is uniformly bounded with respect to $\varepsilon$ in $L^\infty (\Sigma _{{\delta }/{10 \Lambda }})$ (see Theorem 4.1). By the scaling properties of the fractional Laplacian and the fact that *w* solves (2.3), it results that

$$\begin{aligned} (-\partial _{zz})^s w_\varepsilon (z) = \varepsilon ^{-2s} (-\partial _{zz})^s w\left( {z}/{\varepsilon }\right) = -\varepsilon ^{-2s} W'\left( w\left( {z}/{\varepsilon }\right) \right) = - \varepsilon ^{-2s} W'(u_\varepsilon (x)). \end{aligned}$$

Therefore, (6.7) reads as

$$\begin{aligned} I_{\varepsilon , \Lambda } = \varepsilon ^{4s-3} \int _{\Sigma _{{\delta }/{10 \Lambda }} \cap \Omega } \left( \frac{\gamma _{1,s}}{4s-2} H_{\Sigma }(x') \eta _{\varepsilon ,{\delta }/{10 \Lambda }}(z) + \mathcal {R}_{\varepsilon , \Lambda }(x) \right) ^2 \, dx. \end{aligned}$$

Furthermore, using (5.2) and changing variables, we have that

$$\begin{aligned} \varepsilon ^{4s-3} \int _{-{\delta }/{10 \Lambda }}^{{\delta }/{10 \Lambda }} \eta _{\varepsilon ,{\delta }/{10 \Lambda }} (z)^2 \, dz&= \varepsilon ^{-1} \int _{-{\delta }/{10 \Lambda }}^{{\delta }/{10 \Lambda }} \eta _{1,{\delta }/{10 \varepsilon \Lambda }}\left( \frac{z}{\varepsilon }\right) ^2 \, dz = \int _{-{\delta }/{10 \varepsilon \Lambda }}^{{\delta }/{10 \varepsilon \Lambda }} \eta _{1,{\delta }/{10 \varepsilon \Lambda }}(z)^2 \, dz. \end{aligned}$$

Thus, using Lemma 5.3 we have that

6.8 $$\begin{aligned} \lim _{\varepsilon \rightarrow 0^+} \varepsilon ^{4s-3} \int _{-{\delta }/{10 \Lambda }}^{{\delta }/{10 \Lambda }} \eta _{\varepsilon , {\delta }/{10 \Lambda }}(z)^2 \, dz = \int _{\mathbb {R}} \left( \int _{\mathbb {R}} \frac{w'(t+z)}{\left| t\right| ^{2s-1}} \, dt \right) ^2 \, dz = \mu _w. \end{aligned}$$

Thus, since $s >{3}/{4}$ and $\mathcal {R}_{\varepsilon , \Lambda }$ is uniformly bounded with respect to $\varepsilon$ in $L^{\infty }(\Sigma _{{\delta }/{10 \Lambda }})$, we have that

6.9 $$\begin{aligned} \limsup _{\varepsilon \rightarrow 0^+} I_{\varepsilon ,\Lambda } = \frac{\gamma _{1,s}^2}{4(2s-1)^2} \limsup _{\varepsilon \rightarrow 0^+} \varepsilon ^{4s-3} \int _{\Omega \cap \Sigma _{{\delta }/{10 \Lambda }}} \left| H_{\Sigma }(x') \eta _{\varepsilon ,{\delta }/{10 \Lambda }}(z)\right| ^2 \, dx. \end{aligned}$$

We remark that

$$\begin{aligned} \Sigma _{{\delta }/{10 \Lambda }} \cap \Omega \subset \left\{ x'+z N_{\Sigma }(x') :x' \in \pi _{\Sigma }\left( \Sigma _{{\delta }/{10 \Lambda }} \cap \Omega \right) , \ z \in \left( -{\delta }/{10 \Lambda }, {\delta }/{10 \Lambda } \right) \right\} . \end{aligned}$$

Thus, by (6.8), (6.9) and the Coarea formula, we write

$$\begin{aligned} \limsup _{\varepsilon \rightarrow 0^+} I_{\varepsilon , \Lambda }&= \limsup _{\varepsilon \rightarrow 0^+} \varepsilon ^{4s-3} \int _{-{\delta }/{10 \Lambda }}^{{\delta }/{10 \Lambda }} \left| \eta _{\varepsilon ,{\delta }/{10 \Lambda }} (z)\right| ^2 \left( \int _{ \pi _{\Sigma } (\Sigma _{{\delta }/{10 \Lambda }} \cap \Omega ) } \left| H_{\Sigma }(x') \right| ^2 \, d \mathcal {H}^{d-1}(x') \right) \, dz \\  &= \mu _w \int _{ \pi _{\Sigma } (\Sigma _{{\delta }/{10 \Lambda }} \cap \Omega ) } \left| H_{\Sigma }(x') \right| ^2 \, d \mathcal {H}^{d-1}(x'), \end{aligned}$$

Hence, (6.6) follows by the computation above and the fact $\bigcap _{\Lambda \ge \Lambda _0} \pi _{\Sigma } (\Sigma _{{\delta }/{10 \Lambda }} \cap \Omega ) \subset \Sigma \cap {\bar{\Omega }}$.

The case $s = {3}/{4}$. Here, we have $4s-3 = 0$, and hence $\varepsilon ^{4s-3} =1$, for any $\varepsilon > 0$. However, there is an extra factor $\left| \log (\varepsilon )\right| ^{-1}$ in the definition of $\mathcal {G}_{s, \varepsilon }$ and Lemma 5.3 is modified accordingly. Therefore, the proof is similar to the previous case and we leave the straightforward modifications to the reader.

Step 2. Let $\Omega , E \subset \mathbb {R}^d$ be bounded open sets with $\partial \Omega \in C^1$ and $\partial E \in C^2$. By Lemma 2.18, we find a sequence of smooth sets $\{E_j\}_{j \in \mathbb {N}}$ satisfying (*A*1), (*A*2), (*A*3), (*A*4), (*A*5). By the argument shown in the previous step, for any $j \in \mathbb {N}$ we find a sequence $u_\varepsilon ^j \rightarrow \chi _{E_j}$ in $L^1_{\textrm{loc}}(\mathbb {R}^d)$ such that (6.3) holds for $E_j$. By (*A*2) and (*A*4), we have that $\mathcal {H}^{d-1}(\partial E_j \cap {\overline{\Omega }}) \rightarrow \text {Per}(E, \Omega )$. Then, by (*A*3), (*A*4), (*A*5), it is immediate to check that

$$\begin{aligned} \lim _{j \rightarrow \infty } \int _{\partial E_j \cap {\overline{\Omega }}} H_{\partial E_j}(y)^2 \, d \mathcal {H}^{d-1}(y) = \mathcal {W}(\Sigma , \Omega ). \end{aligned}$$

Then, by a diagonal argument we build a sequence $u_\varepsilon \rightarrow \chi _E$ in $L^1_{\textrm{loc}}(\mathbb {R}^d)$ such that (6.3) holds. $\square$

### Remark 6.1

We point out that by a simple modification of the proof of Theorem 1.1 it is possible to check that

$$\begin{aligned} \lim _{\varepsilon \rightarrow 0^+} \left( \mathcal {F}_{s, \varepsilon } + \mathcal {G}_{s,\varepsilon } \right) (u_\varepsilon , \Omega ) = c_\star \mathcal {H}^{d-1}(\partial E \cap \Omega ) + \kappa _{\star } \int _{\partial E \cap \Omega } H_{\partial E}(y)^2 \, d \mathcal {H}^{d-1}(y), \end{aligned}$$

where $s \in \left( {3}/{4},1 \right)$, *E* is a bounded open set of class $C^3$, $u_\varepsilon$ is defined by (6.2) and $\Omega$ is any bounded open set whose boundary intersects transversally $\partial E$, that is $\mathcal {H}^{d-1}(\partial E \cap \partial \Omega ) = 0$. Here, no regularity on $\partial \Omega$ is needed. The same conclusions holds for $s = {3}/{4}$ adding an extra $\left| \log (\varepsilon )\right| ^{-1}$ in front of $\mathcal {G}_{s,\varepsilon }$. We leave the details to the interested reader.

### Remark 6.2

Once Theorem 1.1 is established, we can prove (6.3) for bounded open sets *E* of class $C^2$. Indeed, given any bounded open set $\Omega$ (without further assumptions), we find a sequence of smooth bounded open sets $\Omega _j$ such that $\bigcap _j \Omega _j = \Omega$. Then, using Theorem 1.1 for *E* in $\Omega _j$ and recalling that

$$\begin{aligned} \lim _{j \rightarrow \infty } c_\star \textrm{Per}(E, \Omega _j) + \kappa _\star \mathcal {W}(E, \Omega _j) = c_\star \mathcal {H}^{d-1}(\partial E\cap {\overline{\Omega }}) + \kappa _\star \int _{\partial E \cap {\overline{\Omega }}} H_{\partial E}(y)^2 \, d \mathcal {H}^{d-1}(y), \end{aligned}$$

we build the sequence $\{u_\varepsilon \}$ by a standard diagonal argument.

## Appendix: The exact constants

We collect some useful results on the exact value of some constants involved in our computations. We recall that the Euler Gamma function and Beta function are defined respectively by

$$\begin{aligned} \Gamma (x) = \int _0^{+\infty } t^{x-1} e^{-t} \, dt, \qquad \textrm{B}(x,y) = \int _0^1(1-t)^{x-1} t^{y-1} \, dt \qquad x,y >0. \end{aligned}$$

We recall that

6.10 $$\begin{aligned}  &   \Gamma (x+1) = x \Gamma (x), \qquad \Gamma ({1}/{2}) = \sqrt{\pi },\nonumber \\  &   \qquad \mathcal {H}^{d-1} (\mathbb {S}^{d-1}) = \frac{2 \pi ^{\frac{d}{2}}}{\Gamma \left( \frac{d}{2}\right) }, \qquad \textrm{B}(x,y) = \frac{\Gamma (x) \Gamma (y)}{\Gamma (x+y)}. \end{aligned}$$

### Lemma 6.3

Given $a>-1, b > a+1$, it holds

6.11 $$\begin{aligned} 2\int _0^\infty \frac{ r^a }{ (r^2+1)^{\frac{b}{2}} }\,dr = \frac{ \Gamma \left( \frac{a+1}{2}\right) \Gamma \left( \frac{b-(a+1)}{2}\right) }{ \Gamma \left( \frac{b}{2}\right) }. \end{aligned}$$

### Proof

The computation is straightforward. After the change of variable $t= (r^2+1)^{-1}$, by standard manipulations, we find that

$$\begin{aligned} 2 \int _0^\infty \frac{ r^a }{ (r^2+1)^{\frac{b}{2}} } \, dr&= \int _0^1 t^{\frac{b}{2}} \left( \frac{1-t}{t} \right) ^{\frac{a-1}{2}} \frac{dt}{t^2} = \textrm{B}\left( \frac{b-a-1}{2}, \frac{a+1}{2} \right) . \end{aligned}$$

Hence, (6.11) follows by the above computation and (6.10). $\square$

### Corollary 6.4

Fix $\delta >0$ and $\alpha , \beta \ge 0$ such that $2s+\beta +1 >\alpha$. There exists an explicit constant $M(d,s,\alpha , \beta ) >0$ such that for any $z \in (-\delta , \delta )$ it holds

6.12 $$\begin{aligned} \int _{B_\delta ^{d-1}} \frac{\left| y\right| ^\alpha }{(\left| z\right| ^2 + \left| y\right| ^2)^{\frac{d+2s + \beta }{2}}} \, d y = \mathcal {M}(d,s,\alpha ,\beta ) \left| z\right| ^{\alpha -1-\beta -2s} + O(\delta ^{\alpha -1 -\beta -2s }). \end{aligned}$$

More precisely, it holds

6.13 $$\begin{aligned}  &   \int _{B_\delta ^{d-1}} \frac{1}{(\left| z\right| ^2 + \left| y\right| ^2)^{\frac{d+2s}{2}}} \, d y = \frac{\gamma _{1,s}}{\gamma _{d,s}} \left| z\right| ^{-1-2s} + O(\delta ^{-1-2s}), \end{aligned}$$

6.14 $$\begin{aligned}  &   \int _{B_\delta ^{d-1}} \frac{\left| y\right| ^2}{(\left| z\right| ^2 + \left| y\right| ^2)^{\frac{d+2s}{2}}} \, d y = \frac{(d-1) \gamma _{1,s}}{(2s-1) \gamma _{d,s} } \left| z\right| ^{1-2s} + O(\delta ^{1-2s}), \end{aligned}$$

6.15 $$\begin{aligned}  &   \int _{B_\delta ^{d-1}} \frac{y_i^2}{(\left| z\right| ^2 + \left| y\right| ^2)^{\frac{d+2s}{2}}} \, d y = \frac{1}{2s-1} \frac{ \gamma _{1,s}}{ \gamma _{d,s} } \left| z\right| ^{1-2s} + O(\delta ^{1-2s}) \qquad i = 1, \dots , d-1.\nonumber \\ \end{aligned}$$

The reminders are uniform with respect to $z \in (-\delta , \delta )$.

### Proof

We set

$$\begin{aligned} \mathcal {M}(d,s,\alpha , \beta ):= \int _{\mathbb {R}^{d-1}} \frac{\left| y\right| ^{\alpha }}{(1+\left| y\right| ^2)^{\frac{d+2s+\beta }{2}}} \, dy \end{aligned}$$

and we remark that $M(d,s,\alpha , \beta )$ is finite since $d+2s+\beta - \alpha > d-1$. Then, to check (6.12), we change variables $y= \left| z\right| {\tilde{y}}$ and we compute

$$\begin{aligned}&\int _{B_\delta ^{d-1}} \frac{\left| y\right| ^{\alpha }}{(\left| z\right| ^2 + \left| y\right| ^2)^{\frac{d+2s+\beta }{2}}} \, d y = \left| z\right| ^{\alpha -1-\beta -2s} \int _{B^{d-1}_{{\delta }/{\left| z\right| } }} \frac{\left| {\tilde{y}}\right| ^{\alpha }}{ (1+ \left| {\tilde{y}}\right| ^2) ^{\frac{d+2s +\beta }{2}}} \, d {\tilde{y}} \\  &= \left| z\right| ^{\alpha -1-\beta -2s} \left( \mathcal {M}(d,s,\alpha ,\beta ) - \int _{\left| {\tilde{y}}\right| \ge \frac{\delta }{\left| z\right| } } \frac{\left| {\tilde{y}}\right| ^{\alpha }}{ (1+ \left| {\tilde{y}}\right| ^2) ^{\frac{d+2s+\beta }{2}}} \, d {\tilde{y}} \right) \\  &= \left| z\right| ^{\alpha -1-\beta -2s} \left( \mathcal {M}(d,s,\alpha ,\beta ) - O \left( \left( \frac{\delta }{\left| z\right| } \right) ^{\alpha -1-\beta -2s} \right) \right) . \end{aligned}$$

To prove (6.13) and (6.14), we compute the explicit value of $\mathcal {M}(d,s,\alpha , \beta )$. When $(\alpha , \beta ) = (0,0)$, by Lemmas 6.3, (6.10) and (2.2) we have

$$\begin{aligned} \mathcal {M}(d,s,0,0)&= \int _{\mathbb {R}^{d-1}} \frac{1}{(1+ \left| y\right| ^2)^{\frac{d+2s}{2}}} \, dy \\&= \mathcal {H}^{d-2}(\mathbb {S}^{d-2}) \int _0^{+\infty } \frac{r^{d-2}}{(1+r^2)^{\frac{d+2s}{2}}} \, dr = \pi ^{\frac{d-1}{2}} \frac{\Gamma (\frac{2s+1}{2})}{\Gamma (\frac{d+2s}{2})} = \frac{\gamma _{1,s}}{\gamma _{d,s}}, \end{aligned}$$

Similarly, it is immediate to check that

$$\begin{aligned} \mathcal {M}(d,s, 2,0) = \frac{d-1}{2s-1} \cdot \frac{\gamma _{1,s}}{\gamma _{d,s}}. \end{aligned}$$

Lastly, we observe that (6.15) follows by (6.14), since by symmetry we have that

$$\begin{aligned} \int _{B_\delta ^{d-1}} \frac{y_i^2}{(\left| z\right| ^2 + \left| y\right| ^2)^{\frac{d+2s}{2}}} \, d y = \frac{1}{d-1} \int _{B_\delta ^{d-1}} \frac{\left| y\right| ^2}{(\left| z\right| ^2 + \left| y\right| ^2)^{\frac{d+2s}{2}}} \, d y \qquad i =1, \dots , d-1. \end{aligned}$$

$$\square $$

## Data Availability

Data sharing is not applicable, since no data were used for this research.
